# Guideline-based quality indicators—a systematic comparison of German and international clinical practice guidelines

**DOI:** 10.1186/s13012-019-0918-y

**Published:** 2019-07-09

**Authors:** Monika Becker, Jessica Breuing, Monika Nothacker, Stefanie Deckert, Marie Brombach, Jochen Schmitt, Edmund Neugebauer, Dawid Pieper

**Affiliations:** 1 Institute for Research in Operative Medicine (IFOM), Department Evidence-based health services research, Faculty of Health, Department of Medicine, Witten/Herdecke University, Ostmerheimer Str. 200, Building 38, 51109 Cologne, Germany; 2 AWMF-Institute for Medical Knowledge Management c/o Philipps-University Marburg, Karl-von-Frisch-Straße 1, 35043 Marburg, Germany; 3 Center for Evidence-based Healthcare, University Hospital and Medical Faculty Carl Gustav Carus, TU Dresden, Fetscherstraße 74, 01309 Dresden, Germany; 4 Brandenburg Medical School – Theodor Fontane, Fehrbelliner Str.38, 16816 Neuruppin, Germany

**Keywords:** Guidelines, Quality indicator, Performance measures, Systematic review

## Abstract

**Background:**

Evidence-based clinical practice guidelines (CPGs) are relevant sources for generating quality indicators (QIs). The objective of this study was to compare guideline-based QIs of German and international CPGs and their underlying methodological approaches.

**Methods:**

We conducted systematic searches in the guideline databases of G-I-N (Guidelines International Network) and NGC (National Guideline Clearinghouse) between February and June 2017 to identify international CPGs matching the topics of German evidence-based CPGs (*n* = 35) that report QIs, which were identified in a preceding study. Additionally, we searched the websites of the particular CPG providers for separate documents with regard to QIs. We included evidence-based CPGs which report QIs. Reported QIs, the underlying guideline recommendations, and information on methods of development were extracted. The selection and extraction of CPGs were conducted by one reviewer and checked by another. For each matched pair of CPGs, we assessed whether the suggested QIs matched or were not directly comparable.

**Results:**

Twenty-five international CPGs, originating from seven CPG providers in total, met the criteria for inclusion. They matched the topics of 18 German CPGs. This resulted in 30 CPG pairs for the comparison of QIs (some of the international CPGs matched the topic of more than one German CPG). We found 27 QI pairs with QIs “not different or slightly different”, corresponding to 13% (27 of 212) of the QIs in German CPGs and 16% (27 of 166) in international CPGs. Only two QI pairs were judged to be “different/inconsistent”. For 183 of 212 (86%) QIs from German CPGs and 137 of 166 (83%) QIs from international CPGs, no direct comparison could be made. An explicit link to one or more guideline recommendations was found for 136 of 152 (89%) QIs from German CPGs and 82 of 166 (49%) QIs from international CPGs. Some information on methods for the development of QIs existed for 12 of 18 (67%) German CPGs and 8 of 25 (32%) international CPGs.

**Conclusions:**

The majority of QIs in German and international CPGs were not comparable. Various reasons for this are conceivable. More transparent reporting of the underlying methods for generating guideline-based QIs is needed.

**Electronic supplementary material:**

The online version of this article (10.1186/s13012-019-0918-y) contains supplementary material, which is available to authorized users.

## Background

Quality measurement and improvement play an important role in the provision of healthcare. For this purpose, quality indicators (QIs) can be used. There is no clear-cut definition of a QI. According to Lawrence and Frede, a QI is a “measurable element of practice performance for which there is evidence or consensus that it can be used to assess the quality, and hence change in the quality, of care provided” [1]. The Joint Commission on Accreditation of Healthcare Organizations (JCAHO) defines QIs as “[…] quantitative measures that can be used to monitor and evaluate the quality of important governance, management, clinical, and support functions that affect patient outcomes” [2]. To be deemed as trustworthy and useful, QIs have to satisfy different criteria, such as relevance, validity, reliability, feasibility, and target group orientation [3–6]. To meet the high methodological requirements on QIs, they should be based on scientific evidence and developed in a systematic and transparent way wherever possible [7, 8].

As evidence-based clinical practice guidelines (CPGs) are designed to reflect current best practice, they are relevant sources for generating QIs [7, 9]. The term “guideline-based QIs” specifically indicates QIs that are either generated from already available CPGs or coupled with the process of CPG development [10]. Besides assessing the quality of healthcare, these are important tools to assess the implementation of guideline recommendations [11–13]. However, the methodological approaches to the development of guideline-based QIs vary considerably [10].

In Germany, the AWMF (German Association of the Scientific Medical Societies) provides a methodological framework for the development of CPGs by the scientific medical societies. The guideline classification scheme of the AWMF differentiates between S1-, S2k-, S2e-, and S3-CPGs depending on the methodological approach [14]. Thus, S1-CPGs are based on informal consensus-building. In S2k-CPGs, a formal consensus method is applied in a representative panel, and S2e-CPGs include a systematic approach to literature-searching as well as the selection and appraisal of evidence. S3-CPGs comprise the requirements for both S2k-CPGs and S2e-CPGs and thus have the highest methodological standard in Germany. An analysis of the status quo of reported QIs in German S3-CPGs, performed in 2013, identified 34 S3-CPGs which report 394 different QIs (including measures of quality labeled as “quality criteria” or “quality measure”) [15]. For example, the German S3-CPG “Diagnostics, treatment and follow-up care of malignant ovarian tumors” comprises 12 QIs, one of them concerning counselling by social services (numerator: number of patients with counselling by social services; denominator: all patients with an initial diagnoses of ovarian cancer and treatment in a clinical institution) [16]. A recent update of this analysis with a search up to 2016 (Deckert S, et al: (Wie) erfolgt die Ableitung von Qualitätsindikatoren zur Messung und Bewertung der Versorgungsqualität im Rahmen von S3-Leitlinien? Eine Übersichtsarbeit, submitted) found 35 current German S3-CPGs which report 372 different QIs. Four German S3-CPGs were developed by the National Program for Disease Management Guidelines (NDMG), 15 by the German Guideline Program in Oncology (GGPO), and 16 by various scientific medical societies. Particularly, the CPGs of the NDMG and GGPO have a broad scope and cover various areas of medical care. For these CPGs, the development of guideline-based QIs is obligatory; the methodology is outlined in the corresponding manuals [11–13].

Although a working group of the Guidelines International Network (G-I-N) recently proposed a set of reporting standards for guideline-based performance measures [17], there is currently no gold standard for the development of guideline-based QIs [10, 18]. Moreover, there is a lack of research into the consistency of guideline-based QIs from different CPGs. Our hypothesis is that in many cases, QIs from German S3-CPGs do not correspond with QIs of international CPGs on related topics.

This study was part of the project “Systematic analysis of the translation of guideline recommendations into quality indicators and development of an evidence- and consensus-based standard”, supported by the German Research Association (DFG). Our analysis provided information for another part of the research project, a qualitative study which consisted of structured interviews with developers, methodologists, and users of international guidelines (Bolster M, et al: International experiences in the development of guideline-based quality indicators- a qualitative study, submitted). The intention of both studies was to add information to existing research on methods for the guideline-based development of QIs [10, 17]. The results contribute to a consensus study on standards of the translation of guideline recommendations into quality indicators in Germany.

The objective of this study was to compare guideline-based QIs of the 35 previously identified German S3-CPGs, as well as their underlying methodological approaches, with those of international CPGs on related topics.

## Methods

The study was aligned with the PRISMA guidelines [19], although it did not fulfil all requirements related to a systematic review. The methods were in accordance with those set out in a previously published protocol [20], with the exception of one eligibility criterion that we added later (see below).

### Data sources and the selection of CPGs

#### Eligibility criteria

International CPGs that met the following criteria were included in the study:

- QIs are reported.
- The CPG is an evidence-based CPG.
- The topic and recommendations are comparable with those of at least one of the 35 previously identified German S3-CPGs (see Additional file 1).
- The country of CPG development belongs to WHO-Stratum A [21].
- Date of publication between 2012 and 2017.
- Published in German, English, French, Spanish, Dutch, Norwegian, or Swedish.
- The current full-text version is available at no charge.
- The validity date of the CPG, indicated by the CPG developer, is not exceeded.

In addition to the criteria already mentioned in the protocol, we defined as a basic prerequisite that the document is a CPG with clearly identifiable recommendations.

Whenever QIs were solely reported in a separate document which is not a supplement to the CPG (e.g. evidence or methodological report), they had to be linked explicitly with the particular CPG.

An example for such a separate document containing guideline-based QIs is a document from the website of the National Institute for Health and Care Excellence (NICE): “NICE menu of general practice and clinical commissioning group indicators” [22]. The mentioned NICE-QIs are usually linked with specific CPGs. For example, the NICE indicator NM59 (the percentage of patients with diabetes who have a record of an albumin: creatinine ratio (ACR) test in the preceding 15 months) is linked with the NICE-CPGs NG17 (type 1 diabetes in adults) [23] and NG28 (type 2 diabetes in adults) [24]. Otherwise, we assumed that these QIs are not guideline-based and excluded the CPG.

Evidence-based CPGs were defined in this analysis as CPGs whose recommendations

- Are based on a systematic literature search
- Are clearly identifiable and assigned with a grade of recommendation (GoR) and/or a level of evidence (LoE)
- Are linked to the references of the underlying evidence.

#### Literature search

We conducted systematic searches in the guideline databases of G-I-N and NGC (National Guideline Clearinghouse) between February and June 2017 to identify international CPGs matching the topics of the previously identified German S3-CPGs which report QIs. The search strategies included keywords related to the clinical topics, both as full terms and with appropriate truncations, connected with Boolean operators. For six of the CPGs from the CPG program oncology and for all German S3-CPGs on diabetes, we conducted one combined search each (*carcinoma OR *cancer OR oncolog*; diabet*); for the remaining German S3-CPGs, separate searches were performed (see Additional file 2 for details on search strategies). Furthermore, we crosschecked the reference lists of the German S3-CPGs and the international CPGs eligible for inclusion in the analysis.

In cases we identified international CPGs with eligible topics that comprised neither QIs nor links to QIs, we searched the websites of the particular CPG providers for separate documents describing QIs that were explicitly linked with the particular CPG.

#### Selection process

One reviewer screened the titles of records. The full texts of those deemed eligible for inclusion were retrieved. Subsequently, full texts were screened by one reviewer and checked by another. The reasons for exclusion were documented, and any disagreements were resolved through discussion and consensus.

In cases where no eligible international CPG matching the topic of a German S3-CPG could be found, we excluded that German S3-CPG from the analysis.

### Data extraction

A standardized data extraction form was developed based on the items used in a previous project on the evaluation of QIs reported in German S3-CPGs [15] and then piloted. For each included matched CPG pair, we extracted only QIs on clinical topics (e.g. screening, diagnostics, therapy, or rehabilitation) that were addressed in both CPGs. For example, if only one of the matched CPGs dealt with the clinical topic “diagnostics”, we did not consider QIs on that topic. Furthermore, we collected the following information:

- Number of members and expertise of the QI development group (such as methodologists, clinicians, patient representatives)
- Label of the quality measure, e.g. QI, quality criteria and performance measure
- Categorization of QI into structure, process, or outcome indicators according to the definition of Donabedian [25] (in case of missing assignment by the guideline authors, our own assignment was made)
- Underlying recommendations and whether the QIs were based explicitly or implicitly on those
- Rationale reported for the QI
- Scientific measurement properties reported for the QI, e.g. reliability and validity [26]
- Intended purpose reported for the QI, e.g. quality reporting, quality management systems, and evaluation of CPGs
- Quality objectives reported
- Methods used for QI development, e.g. search for existing QIs, consensus methods, and assessment tools

Data were extracted by one reviewer and checked by another, and any disagreements were resolved through discussion and consensus.

### Quality appraisal

As trustworthy guideline-based QIs should be based on high-quality CPGs [10, 17], we appraised the methodological quality of all included German S3- and international CPGs using the domain “Methodological Rigor of Guideline Development” of the German Instrument for Methodological Guideline Appraisal (DELBI) [27]. Seven items were rated on a 4-point scale (wherein one = “strongly disagree”, two = “disagree”, three = “agree”, and four = “strongly agree”):

- Systematic methods were used to search for evidence.
- The criteria for selecting the evidence are clearly described.
- The methods used for formulating the recommendations are clearly described.
- Health benefits, side effects, and risks have been considered in formulating the recommendations.
- There is an explicit link between the recommendations and the supporting evidence.
- The guideline has been externally reviewed by experts prior to its publication.
- A procedure for updating the guideline is provided.

Two reviewers performed quality assessment independently. In case the appraisal of the two reviewers differed by two or more points, disagreements were resolved through discussion and consensus. The domain score was calculated by summing up the scores of individual items and by standardizing the total as a percentage of the maximum possible score for the domain (4 (strongly agree) × 7 (items) × 2 (appraisers)) [27].

In case reviewers had been involved in the development of an included CPG, they did not participate in their quality assessment.

### Data synthesis

Data synthesis involved a descriptive analysis and a tabular comparison of the QIs of the international and German S3-CPGs for each clinical topic and, where applicable, for each underlying recommendation. We collected the number of CPGs that provided information on the QI development group, methods of QI development, as well as the rationale and intended purpose of QIs. On the basis of reported QIs, we collected the number of QIs for which quality objectives and measurement properties were reported as well as the number of QIs that were explicitly or implicitly based on guideline recommendations.

For each matched pair of CPGs, we compared the suggested QIs and assessed whether the QIs matched or not.

Our definition of QI-matching was that both QIs on the same clinical topic either agreed or disagreed in content and definitions regarding a specific clinical issue, e.g. a specific intervention or diagnostic procedure and either addressed or did not address the same population. Then, we assigned QIs either to the category “not different/slightly different” or “different/inconsistent”. QIs were considered not to match whenever no direct comparison could be made because the QIs differed fundamentally in contents and definitions. Thus, the QIs either addressed different specific issues within a clinical topic, or were reported in only one of the matched CPGs, even though both CPGs addressed that particular clinical topic. For example, the topic “screening” was addressed by both CPGs of a matched pair, but only one had defined QIs for that topic. Those QIs were extracted under the category “QI only defined in the international or the German S3-CPG”, respectively. For each of the categories described above, we collected the number of QIs or QI pairs. The assignment of the QIs to the categories was conducted by one reviewer and checked by another. Disagreements were resolved through discussion and consensus. Furthermore, the methods described for QI development were presented as a narrative summary.

## Results

### Results of the literature search and characteristics of included CPGs

**Fig. 1:**
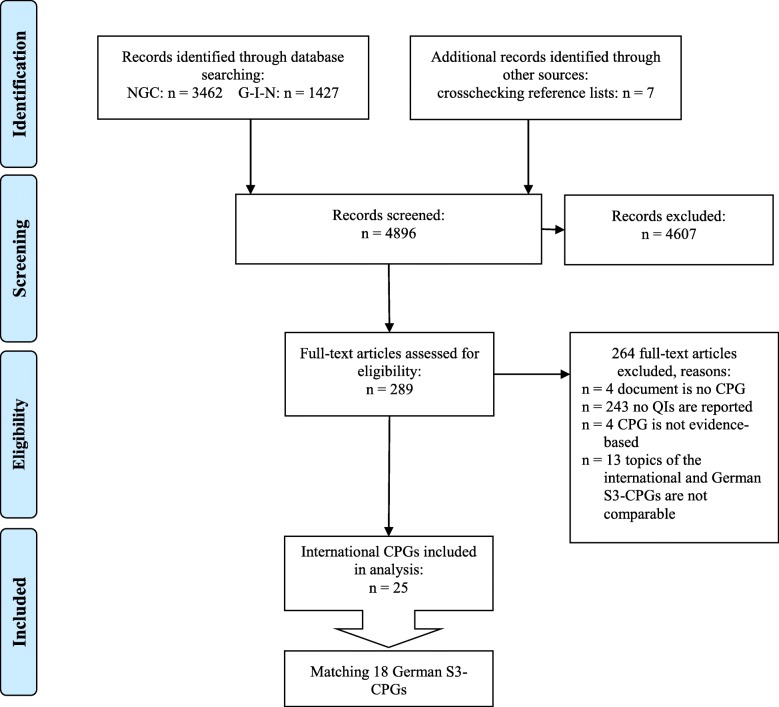
Flow diagram for the search and selection of international CPGs

The searches in the CPG databases identified 4889 records. We found seven additional potential eligible international CPGs by crosschecking the reference lists of included CPGs. After the initial screening of the titles, 289 full texts were reviewed, out of which 264 were excluded (see Additional file 3). The most common reason for exclusion was that no QIs were reported. The remaining 25 international CPGs [23, 24, 28–50], originating from seven CPG providers, met the criteria for inclusion. The screening process is summarized in a flow chart (Fig. 1).

The 25 included international CPGs matched the topics of 18 of the 35 German S3-CPGs [16, 51–67]. Eight and three of the German S3-CPGs were developed by the GGPO and NDMG, respectively. Seven German S3-CPGs originated from other German medical societies. We excluded those 17 German S3-CPGs from the analysis for which we found no eligible international CPG with matching topics. This resulted in 30 CPG pairs for the comparison of Qis, as some of the international CPGs matched the topic of more than one German S3-CPG. Table 1 gives an overview of the CPG pairs analysed.

**Table 1:** CPG pairs identified

CPG pair no.	Topic	German S3-CPG (abbreviation)	CPG pair (*n*)	International CPG (abbreviation)
1	Breast cancer	032/045OL 2012 [61]	1	SIGN breast 2013 [42]
2	Ovarian cancer	032/035OL 2013 [16]	1	SIGN ovar 2013 [44]
3	Prostate cancer	043/022OL 2014 [51]	1	CTFPHC prostate 2014 [49]
4	Colorectal cancer	021/007OL 2013 [58]	2	CTFPHC colorectal 2016 [48]
5				SIGN colorectal 2016 [33]
6	Oesophagus cancer	021/023OL 2014 [64]	1	KCE gastrointest 2012 [28]
7	Gastric cancer	032/009OL 2012 [60]	1	KCE gastrointest 2012 [28]
8	Palliative medicine	128/001OL 2015 [65]	1	ICSI palliative 2013 [45]
9	Melanoma	032/024OL 2016 [62]	1	SIGN melanoma 2017 [43]
10	Low-back pain	nvl/007 2015 [52]	1	ICSI backpain 2012 [39]
11	Kidney disease in diabetes	nvl/001d 2015 [63]	3	NICE diabtypeI 2015 [23]
12				NICE diabtypeII 2016 [24]
13				SNS diabtypeI 2012 [35]
14	Diabetes training	nvl/001f 2012 [53]	4	NICE diabtypeI 2015 [23]
15				NICE diabtypeII 2016 [24]
16				SNS diabtypeI 2012 [35]
17				ICSI diabtypeII 2014 [36]
18	Obesity	050/001 2014*^)^ [54]	4	CTFPHC obesity 2015 [50]
19				NICE obesity 2014 [29]
20				NICE weight 2014 [30]
21				ICSI obesity 2013 [31]
22	Diabetes and pregnancy	057 – 023 2014*^)^ [56]	2	NICE diabpreg 2015 [34]
23				SNS diabtypeI 2012 [35]
24	Bipolar disorder	038 – 019 2012*^)^ [55]	1	NICE bipolar 2016 [32]
25	Hysterectomy for benign diseases	015 – 070 2014*^)^ [57]	1	NICE menstrual bleeding 2016 [38]
26	Long-term opioid-use in non-cancer pain	145 – 003 2014*^)^ [59]	2	ICSI pain 2016 [40]
27				SIGN pain 2013 [41]
28	Venous thromboembolism	003 – 001 2015*^)^ [66]	2	SIGN VTEPrev 2014 [47]
29				CCHMC VTE 2014 [46]
30	Perioperative hypothermia	001 – 018 2013*^)^ [67]	1	ICSI hypo 2014 [37]

*CCHMC* Cincinnati Children's Hospital Medical Center, *CTFPHC* Canadian Task Force on Preventive Health Care, *ICSI* Institute for Clinical Systems Improvement, *KCE* Belgian Healthcare Knowledge Centre, *NICE* National Institute for Health and Care Excellence, *SIGN* Scottish Intercollegiate Guidelines Network, *SNS* (Spanish) National Health System, *OL* CPG of the German Guideline Program in Oncology, *nvl* CPG of the National Program for Disease Management Guidelines

*^)^CPG of the scientific medical societies

Our assessment of methodological quality of the included CPGs gave a mean standardized score of 69% (standard deviation 7.8) for the domain “Methodological Rigor of Development” for the German S3-CPGs and 62% (standard deviation 12.7) for the international CPGs. For the individually rated items and resulting scores for each CPG, see Additional file 4.

### Characteristics of guideline-based QIs

Overall, the German S3-CPGs and international CPGs contained 152 and 166 QIs on related topics, respectively. The median number of QIs per CPG was 8 (range 0–37) in the German S3-CPGs and 4.5 (range 1–15) in the international CPGs. With regard to the 30 CPG pairs, we compared 212 QIs from German S3-CPGs to 166 QIs from international CPGs (some of the QIs from German S3-CPGs were counted more than once as we found more than one international CPG related to some German S3-CPGs). The QIs in 85% of German S3-CPGs (129 of 152) and 84% of international CPGs (139 of 166) were presented as ratios or proportions (defining numerator and denominator or quoting percentages).

In 17% (3 of 18) of German S3-CPGs and 28% (7 of 25) of international CPGs, a categorization of QIs into structure, process, or outcome indicators was made by the CPG authors themselves. According to our own assignment, we found mainly process indicators: 123 of 152 (81%) in the German S3-CPGs and 133 of 166 (80%) in the international CPGs. However, for 12 of 64 (19%) QIs, we disagreed with the categorisation made by the authors of the international CPGs and therefore changed the category. For all nine QIs that were categorised by the authors of the German S3-CPGs, we agreed with the assignment.

The intended purpose of the QIs was reported in 13 of 18 (72%) German S3-CPGs and in 21 of 25 (84%) international CPGs. The rationale for the QIs was stated in only one of 18 (6%) German S3-CPGs and in one of 25 (4%) international CPGs.

An explicit link to one or more guideline recommendations was found for 136 of 152 (89%) and 82 of 166 (49%) QIs from 15 German S3 and 12 international CPGs, respectively.

Among these, 77% (104 of 136) of QIs from German S3-CPGs and 93% (76 of 82) from international CPGs were based on strong recommendations. Of these strong recommendations, 43% (45 of 104) in the German S3-CPGs were consensus-based. This means they were based on the expert opinion of the CPG group, given that none or insufficient evidence exists for generating an evidence-based recommendation (in some CPGs those recommendations referred also to as “good clinical practice”). No recommendation in international CPGs was explicitly stated to be consensus-based, but all were evidence-based. However, the quality of the underlying evidence in five international CPGs from KCE and ICSI is mostly designated as “low” or “moderate”. The underlying evidence of the strong recommendations in the seven included CPGs by NICE was mostly not clearly stated. For one of 152 (0.7%) QIs in the German S3-CPGs and 23 of 166 (14%) in the international CPGs, we found an implicit connection, as we identified one or more corresponding recommendation(s) in the particular CPG.

Quality objectives were stated for 39 of 152 (26%) QIs in the German S3-CPGs and for 39 of 166 (23%) QIs in the international CPGs. Properties were not reported for any QI measurement.

**Fig. 2:**
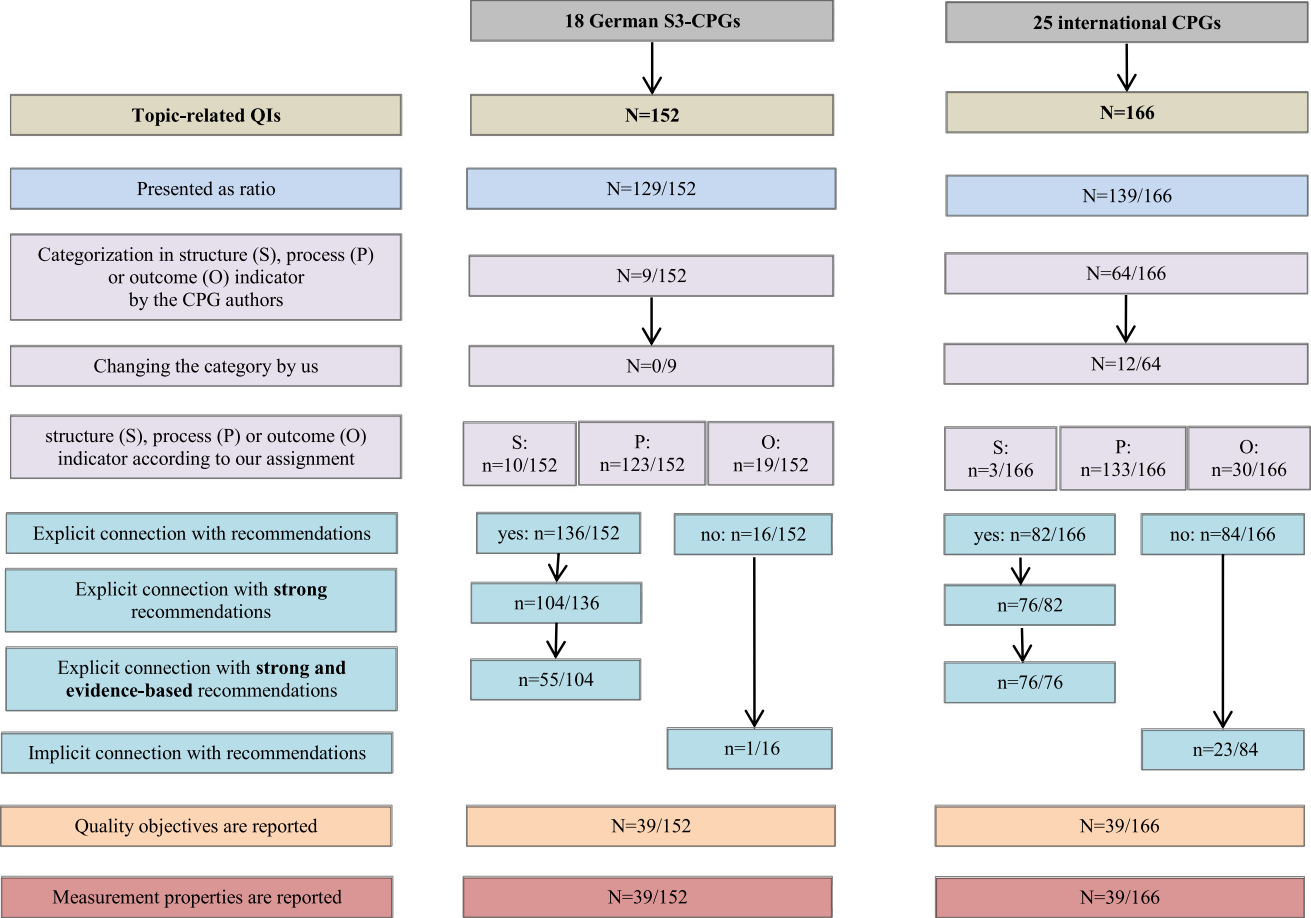
Overview of QIs

An overview of the QIs is presented in Fig. 2. Table 2 differentiates between responsible organisations within the German S3-CPGs.

**Table 2:** Information on QIs with differentiation among German S3-CPGs

	German S3-CPG			German S3-CPG (*n* = 18)	International CPG (*n* = 25)
	Scientific medical societies (*n* = 7)	NDMG (*n* = 3)	GGPO (*n* = 8)		
Categorization of QI into structure (S), process (P), or outcome (O) indicator (according to own assignment)					
S(*n*)/P(*n*)/O(*n*)	10/48/5	0/14/0	0/61/14	10/123/19	3/133/30
Intended purpose of QI is reported					
Yes (*n*)	3	2	8	13	21
No (*n*)	4	1	0	5	4
Rationale for QI is reported					
Yes (*n*)	1	0	0	1	1
No (*n*)	6	3	8	17	24
QI is presented as ratio/proportion					
*n*/*N*	40/63	14/14	75/75	129/152 (85%)	139/166 (84%)
QI is based explicitly on one or more recommendations					
*n*/*N* of that at least one strong recommendation/statement	53/63 45%	14/14 100%	69/75 97%	136/152 (89%) 77%	82/166 (49%) 93%
Measurement properties are reported					
*n*/*N*	0/63	0/14	0/75	0/152	0/166
Quality objectives are reported					
*n*/*N*	7/63	7/14	25/75	39/152 (26%)	39/166 (23%)

### Comparison of QIs

Twelve of the 30 CPG pairs comprised 27 QI pairs that were “not different or slightly different”. This corresponds to 13% (27 of 212) of the QIs in German S3-CPGs and 16% (27 of 166) in international CPGs. Only two QI pairs were judged to be “different/inconsistent”. For the majority of Qis, no direct comparison could be made, i.e. those QIs were found only in either the international or the German S3-CPGs (Table 3). Examples for all categories are presented in Table 4. All extracted QIs and corresponding recommendations can be found in Additional file 5 (QIs and recommendations out of German S3-CPGs were extracted only in German). Furthermore, a detailed comparison of all QIs on related topics is shown in Additional file 6 (the number of the QIs correspond to those stated in Additional file 5).

**Table 3:** Comparison of QIs

30 CPG pairs	QI match (QI pair)		No match	
	QI “not different or slightly different”	QI “different/inconsistent”	QI only in international CPG	QI only in German S3-CPG
	*n* = 27	*n* = 2	*n* = 137	*n* = 183

**Table 4:** QIs on related topics in international and German S3-CPGs with corresponding recommendations (examples)

Acronym international CPG	QI_int_[# (S/P/O); reference range rr; title]	Corresponding recommendation(s) (GoR, LoE) (explicit/implicit connection)	Acronym corresponding German S3-CPG	QI_S3_* [# (S/P/O); reference range rr; title]	Corresponding recommendation*) (GoR, LoE) (explicit/implicit connection)
QIs “different/inconsistent”					
SIGN melanoma 2017	#_int_3 (P); rr: 95% Multi-Disciplinary Team Meeting (MDT) Numerator: Number of patients with cutaneous melanoma discussed at the MDT before definitive treatment (wide local excision, chemotherapy/SACT, supportive care and radiotherapy). Denominator: All patients with cutaneous melanoma. (Exclusions: Patients who died before first treatment)	All patients with a diagnosis of melanoma should be discussed at a specialist multidisciplinary team (MDT) meeting (GPP). (implicit connection)	032/024OL 2016	#_S3_10 (P); rr: N.R. Presentation melanoma team meeting Numerator: Patients with stage IV melanoma, who are presented in an interdisciplinary team meeting Denominator: Patients with stage IV melanoma	3.146 Patients with metastatic melanoma (as of stage III) should be presented in an in an interdisciplinary team meeting to discuss further diagnostic and therapy. […] (strong rec., consensus-based) (explicit connection)
SIGN ovar 2013	#_int_9 (P); rr: 90% First-line Chemotherapy Numerator: Number of epithelial ovarian cancer patients who receive chemotherapy treatment involving either paclitaxel in combination with a platinum-based compound or carboplatin only Denominator: All epithelial ovarian cancer patients (Exclusions: • Patients with low-grade serous disease. • Patients with FIGO stage 1a or 1b, low grade (G1) disease. • Patients with Stage 1a clear cell tumours. • Patients who decline chemotherapy treatment.)	#_int_9: First line chemotherapy treatment of epithelial ovarian cancer should include a platinum agent either in combination or as a single agent, unless specifically contraindicated (GoR: A, LoE: 1^++^). Carboplatin is the platinum drug of choice in both single and combination therapy (GoR: A, LoE: 1^++^). (implicit connection)	032/035OL 2013	#_S3_10 (P); rr: N.R. Combination therapy for platinum sensitive relapse Numerator: Number of patients with a platinum-based combination therapy Denominator: All patients with platinum-sensitive relapse of an ovarian carcinoma and chemotherapy, outside of clinical studies	9.5 Patients with platinum-sensitive relapse of an ovarian carcinoma should receive with a platinum-based combination therapy if there is the indication for chemotherapy (strong rec., consensus-based). […] (explicit connection)
QIs “not different/slightly different”					
KCE gastrointest 2012	#_int_1 (P); rr: N.R. Staging Numerator: All patients diagnosed with oesophageal cancer in a given year discussed at the multidisciplinary team (MDT) meeting within 1 month after incidence date. Denominator: All patients diagnosed with oesophageal cancer in a given year.	All patients diagnosed with oesophageal cancer should be discussed at a multidisciplinary meeting (GoR: strong, LoE: low). (explicit connection)	021/023OL 2014	#_S3_4 (P); rr: N.R. Therapy recommendation from multidisciplinary tumour conference Numerator: Number of patients with therapy recommendation from multidisciplinary tumour conference before therapy (staging completed) Denominator: All patients with oesophageal cancer	Therapy recommendations should be made in a multidisciplinary tumour conference. […] (strong rec., consensus-based). (explicit connection)
NICE diabtypeI 2015 and NICE diabtypeII 2016	#_int_1 (P); rr: N.R. NM27 The percentage of patients newly diagnosed with diabetes, on the register, in the preceding 1 April to 31 March who have a record of being referred to a structured education programme within 9 months after entry on to the diabetes register.	NICE diabtypeI 2015: rec 1.3.1 Offer all adults with type 1 diabetes a structured education programme of proven benefit, for example the DAFNE (dose-adjustment for normal eating) programme. Offer this programme 6–12 months after diagnosis (strong rec). NICE diabtypeII 2016: rec 1.2.1 Offer structured education to adults with type 2 diabetes and/or their family members or carers (as appropriate) at and around the time of diagnosis, with Type 2 diabetes in adults: management annual reinforcement and review. Explain to people and their carers that structured education is an integral part of diabetes care (strong rec). (explicit connections) NICE diabtypeII 2016: rec 1.2.2 […]	nvl/001f 2012	#_S3_1 (P); rr: N.R. Numerator: Number of patients, for which the offer of a structured education program is documented directly after the diagnosis is being made Denominator: All people with newly diagnosed diabetes mellitus	2-1 Each human with diabetes mellitus and if necessary important reference persons (e.g. relatives) should be offered a structured education program as an indispensable component of the diabetes management directly after the diagnosis is made and regularly in the course of the disease (GoR ⇑⇑). (explicit connection)
ICSI backpain 2012	#_int_3 (P); rr: N.R. Numerator: Number of patients for whom the clinician ordered imaging studies during the six weeks after pain onset, in the absence of "red flags." Denominator: Number of patients with non-specific back pain diagnosis.	Annotation #11 • […] • Clinicians should not recommend imaging (including computed tomography (CT), magnetic resonance imaging (MRI) and x-ray) for patients with non-specific low back pain (strong rec, moderate quality evidence). […] (explicit connection)	nvl/007 2011	#_S3_2 (P); rr: N.R. Imaging techniques for acute back pain Numerator: Number of patients for which imaging diagnostics is conducted without reason Denominator: All patients with acute back pain and without “red flags” after anamnesis and clinical examination.	3-5 Imaging diagnostics is not recommended in case of acute back pain after exclusion of dangerous conditions by anamnesis and clinical examination (GoR ⇓⇓). (explicit connection)
NICE bipolar 2016	#_int_2 (P); rr: N.R. NM16 The percentage of patients with schizophrenia, bipolar affective disorder and other psychoses who have a record of BMI in the preceding 15 months	Rec 1.2.12 Ensure that the physical health check for people with bipolar disorder, performed at least annually, includes: • weight or BMI, diet, nutritional status and level of physical activity • […] (explicit connection)	038/019 2012	#_S3_18 (P); rr: N.R. General principle Numerator: Percentage of patients, for whom weight data are documented repeatedly. Denominator: All patients	Therapy-general principle 4 Regular weight controls should be conducted because of possible weight gain, especially during therapy with mirtazapine, tricyclic antidepressants, lithium, valproic acid, clozapine, olanzapine, quetiapine, risperidone, and zotepine. (moderate rec., consensus-based) (explicit connection)
QIs not comparable (“QI only in international respectively S3-CPG”)					
ICSI diabtypeII 2014	#_int_1 (P); rr: N.R. Numerator: Number of patients who are advised about lifestyle modification and nutrition therapy within one year of diagnosis. Denominator: Number of patients ages 18–75 years old who have T2DM.	Nutrition therapy A qualified health professional (which may include a clinician, dietitian, nursing staff and pharmacist) should provide nutrition therapy to a patient diagnosed with T2DM as part of a global treatment plan (GoR: strong, quality of evidence: moderate). (explicit connection)	nvl/001f 2012	#_S3_1 (P); rr: N.R. Numerator: Number of patients, for which the offer of a structured education program is documented directly after the diagnosis is being made Denominator: All people with newly diagnosed diabetes mellitus	2-1 Each human with diabetes mellitus and if necessary important reference persons (e.g. relatives) should be offered a structured education program as an indispensable component of the diabetes management directly after the diagnosis is made and regularly in the course of the disease (GoR ⇑⇑). (explicit connection)
SIGN VTEPrev 2014	#_int_1 (P); rr: N.R. Compliance with and recording of risk assessment in all patients admitted to or presenting acutely at hospital.	All patients admitted to hospital or presenting acutely to hospital should be individually assessed for risk of VTE and bleeding. The risks and benefits of prophylaxis should be discussed with the patient (GoR: D). (implicit connection)	003/001 2015	_S3_2 (P); rr: ≥ 95 % Proportion of patients with documented information about benefits, risks and alternatives of prophylactic interventions in relation to all patients receiving VTE prophylaxis.	3.8 The conducted risk assessment of a VTE and the resulting interventions of a VTE prophylaxis have to be discussed with the patient regarding benefits, risks and alternatives (according to legal requirements) (GoR ⇑⇑) (explicit connection)
ICSI pain 2016	_int_4 (P); rr: N.R. Numerator: Number of patients with new opioid prescriptions that are <= 20 pills or 3 days’ supply of short-acting opioid. Denominator: Number of patients with chronic pain diagnosis with a new opioid prescription (no opioid prescription for at least 90 days). Exclude patients with an opioid prescription for cancer, migraine and end-of-life care.	Acute or acute on chronic pain • The first opioid prescription for acute pain should be no more than 20 low-dose, short-acting opioids or three days of medication, whichever is less. The total dose for acute pain should not exceed 100 MME. • For patients presenting in acute pain, already on chronic opioids, opioid tolerant or on methadone, use the same pill and dose limits as for opioid-naïve patients (strength of rec. N.R.). (explicit connection)	145/003 2014	# _S3_1 (P); rr: N.R. Number of patients with somatoform pain disorders, which receive opioid analgesics.	Pain associated with functional/somatoform disorders should not be treated with opioid analgesics (consensus-based). (explicit connection)

### Methods for the development of QIs

Information on how QIs were developed was provided in 12 of 18 (67%) German S3-CPGs and eight of 25 (32%) international CPGs. Nine of the German S3-CPGs and one of the international CPGs [28] searched for and reported external data sources for QIs already in existence. Three international CPGs [37, 43, 44] referred to QIs that were developed by an institution that was not involved in the development of the particular CPG, such as the Scottish Cancer Taskforce. The application of formal methods for adopting existing QIs is reported in 12 of 18 (67%) German S3-CPGs and in one of 25 (4%) international CPGs. The use of formal criteria or tools to assess QIs is reported in 12 of 18 (67%) German S3-CPGs and in eight of 25 (32%) international CPGs.

Regarding the underlying evidence for QIs in the German S3-CPGs of NDMG and GGPO, it is stated that QIs should be derived from strong recommendations. This methodological approach was implemented in 11 of the 18 (61%) German S3-CPGs. None of the CPGs of the scientific medical societies gave information on underlying evidence. Among the international CPGs, eight of the 25 (32%) CPGs originating from KCE and NICE provided information on which recommendation or grade of recommendation should be considered. For the KCE-CPG, it was explicitly stated that only strong recommendations were considered for the derivation of QIs. The NICE-CPGs required proposed QIs to be linked by evidence to improved outcomes. For the remaining 17 international CPGs, no information was given.

None of the QIs from German S3-CPGs were reported to be piloted or evaluated, whereas eight international CPGs included a report on pilot testing during the development of QIs. Those international CPGs originated from only two CPG providers (KCE and NICE).

An overview on methodological aspects is presented in Table 5.

**Table 5:** Information on methodological aspects for development of guideline-based QIs

	German S3-CPG			German S3-CPG (*n* = 18)	International CPG (*n* = 25)
	Scientific medical societies (*n* = 7)	NDMG (*n* = 3)	GGPO (*n* = 8)		
Searches for existing QI					
Yes (*n*)	1	3	5	9	1
No/not reported (*n*)	6	0	3	9	24
External data sources (reference to published QI)					
Yes (*n*)	3	0	6	9	4
No/not reported (*n*)	4	3	2	9	25
Formal consensus procedures for adopting QI					
Yes (*n*)	1	3	8	12	1
No/not reported (*n*)	6	0	0	6	24
Use of formal criteria/tools for assessment of QI					
Yes (*n*)	1	3	8	12	8
No/not reported (*n*)	6	0	0	6	17
Piloting/ evaluation of QI					
Yes (*n*)	0	0	0	0	8
No/not reported (*n*)	7	2	7	16	17
Planned (*n*)	0	1	1	2	0

### Information on the composition of the QI development group

Information on the composition of the QI development group was given in 14 of 18 (78%) German S3-CPGs and in 12 of 25 (48%) international CPGs. In the international CPGs, this information originated from three CPG providers (KCE, NICE, and SIGN). In four German S3-CPGs and 13 international CPGs, no information on the QI development group was given.

Clinicians, methodologists, and representatives of cancer registries were involved in the development of QIs of the KCE-CPG. According to the process guide of NICE, the QI development groups were multidisciplinary (e.g. clinicians, methodologists, public health and social care practitioners, patient representatives). However, there was no information on the actual composition of QI development groups for each individual included NICE-CPG. In one SIGN-CPG, it is stated that the QIs were defined by the CPG group.

Among the German S3-CPGs, all CPGs of the NDMG and GGPO and three CPGs developed by scientific medical societies gave information on the QI development group. For seven of the included German S3-CPGs, the QI development group comprised clinicians of different medical specialties, methodologists, and patient representatives, and in another seven German S3-CPGs, a participation of patient representatives was not reported.

An overview on the information on QIs, methods of development, and composition of QI development groups is given in an additional file for each included CPG (Additional file 7).

## Discussion

Our analysis found that the majority of QIs in different CPGs on the same clinical topic was not comparable, but that they vary greatly in content and definitions. This result confirms our hypothesis that in many cases, QIs from German S3-CPGs do not correspond with QIs of international CPGs on related topics. However, only two QI pairs were rated as substantively “different/inconsistent”. Although we suggested a hypothesis, we decided not to perform statistical testing due to the heterogeneous nature of the CPGs. They varied greatly for example in time period of literature searches, publication dates, developing organisation, and health care context as well as in the scope.

Detailed information on the methodological approach to generating QIs was lacking. Only two CPG providers of included international CPGs (NICE and KCE) reported information on the methods used to develop QIs. However, information was missing in these cases as well, such as reporting of the selection and extraction of CPG recommendations and their translation into QIs. Among the German S3-CPGs, all CPGs of the NDMG and the GGPO provided information on methods, whereas almost none of the CPGs of the medical societies contained methodological information. The quality appraisal score for the domain “Methodological Rigor of Development” ranged from 50 to 83% and from 48 to 83% in the German S3-CPGs and international CPGs, respectively. High scores were not inevitably related to better description of the methods of developing the QIs or better reporting of QIs. Although it is assumed that the degree of credibility of QIs is associated with the methodological quality of CPGs, the evidence for this is lacking so far.

### Reasons for differences in QIs

Various reasons are conceivable that would explain that QIs of different CPGs on the same clinical topic often did not cover the same quality aspect of care. One factor could be the different methodological approaches, e.g. to defining selection criteria for recommendations, to appraising the relevance of a QI for health care improvement, or to assessing feasibility of measurement. However, because it was rarely reported how QIs were generated (especially in the included international CPGs), we were unable to analyse this point in further detail. Therefore, for a better understanding of how guideline-based QIs are generated, a better reporting of the underlying processes is necessary. A proposal for reporting standards for guideline-based performance measures has been developed by a working group of G-I-N [17].

Furthermore, although we compared only QIs on clinical topics that were addressed in both CPGs of a CPG pair, several recommendations of the German S3-CPGs and the related international CPGs varied to some extent in content and definitions. Most of the recommendations reported in international and German S3-CPGs were not inconsistent but had a different focus or depth of detail. For example, the German S3-CPG “Type 2 diabetes training” recommended to offer a structured education program, whereas the international CPG conducted by ICSI on “Diagnosis and Management of Type 2 Diabetes Mellitus in Adults” comprised a specific recommendation of nutrition therapy. Nutrition therapy was also considered in the particular German S3-CPG within the explanatory text. However, no specific recommendation on nutrition was made. Further, there were other cases where both CPGs of a CPG pair comprised comparable recommendations, but only in one of the CPGs, a QI was derived from the recommendation(s).

Also, different definitions of QIs may result from an inconsistent composition of the QI development groups with methodologists, relevant health care professionals, stakeholders, and patients. A study about the consistency of QI selection for cardiovascular risk management across different consensus methods and panels found, in part, considerable variation, but could not explain the underlying factors [68]. Further reasons may relate to contextual differences between countries and different health care problems. Regarding guideline-based QIs, another factor could be the up-to-dateness of the CPGs. Many CPGs become out-of-date after about five years [69]. However, in fast-evolving medical fields, recommendations could become out-of-date even earlier.

The analyses of the two inconsistent QI pairs found that the underlying recommendations are also inconsistent, even though the link between QI and recommendation in the international CPG is only implicit. For example, the SIGN-CPG on ovarian cancer recommends that first-line chemotherapy should include a platinum agent either in combination or as a single agent [44], whereas the German S3-CPG recommends solely platinum-based combination therapy [16]. For inconsistent recommendations, various reasons are conceivable likewise, such as differences in the underlying evidence that was used, in the assessment of the evidence, in the composition of the CPG development group, and in value judgements as well the health care context.

### Studies comparing QIs from different countries

Studies on the transferability of non-guideline-based QIs between the USA and the UK and between the USA and the Netherlands found that about 56% and 67%, respectively, were “exactly or nearly equivalent” or “(nearly) identical” [70, 71]. According to the authors, the main reasons for differences seemed to be related to differences in clinical practice or variation in professional culture and expert opinion. The consistency between QIs in our analysis is considerably smaller: only 13% of the QIs in the German S3-CPGs had international equivalents. This discrepancy may be explained by the fact that our analysis focused solely on guideline-based QIs, whereas the QIs in the other studies were derived from a broader literature basis. Furthermore, disparities may be explained by different categorisation of QIs as “nearly equivalent/identical” and “slightly different”. However, this aspect is difficult to assess as definitions for “nearly equivalent/identical” are missing in the studies. In a recent study, Petzold et al. (2018) compared QIs from German S3-CPG with quality measures in NICE quality standards [72]. NICE quality standards consist of statements designed for quality improvements within a particular area of health, each statement being related to quality measures which support their implementation [73]. They are based on NICE guidelines and other NICE-accredited guidance [73]. NICE indicators also measure outcomes considered to reflect the quality of care or processes [73]. In contrast to the quality measures in NICE quality standards, the latter are generally linked directly to specific NICE CPG recommendations. Petzold et al. found that only 34 of 128 (27%) German QIs and 34 of 468 (7%) NICE quality measures they analysed related to the same medical problem [72]. As in our analysis, the consistency between QIs is considerably smaller than in the studies on the transferability of non-guideline-based QIs from different countries. However, the results in the study of Petzold et al. correspond only modestly with the results of our analysis, even if we would separate the NICE-CPGs in our analysis. This could be explained by the fact that we only considered QIs that are directly linked with a CPG as reported, for example, in the “NICE indicator menu” [22]. Petzold et al. exclusively considered quality measures in NICE quality standards that are relevant to NICE-CPGs. We did not consider those because the connection between quality measure and CPG is only indirect. As a result, the two analyses included different German S3-CPGs and NICE-CPGs and, accordingly, different QIs.

### QIs and the underlying evidence

Our analysis found that over 40% of the strong recommendations in the German S3-CPGs are based exclusively on the expert opinion of the CPG group. Furthermore, the quality of the underlying evidence of many strong recommendations in the international CPGs was designated as “low” or “moderate”. This appears to contradict the methodological requirement that QIs should be based on scientific evidence, where possible [7, 8]. However, it might seem reasonable to derive QIs from expert opinion in cases where none or limited evidence exists and a great potential for quality improvement is seen nevertheless by the CPG group. In cases of strong evidence-based recommendations with low or moderate quality of the evidence, it should be noted that various criteria other than the underlying evidence influence the decision about the grade of recommendation, such as clinical relevance, practical experience, risk-benefit ratio, and applicability to clinical practice. The Grading of Recommendations Assessment, Development, and Evaluation (GRADE) system of rating the quality of evidence and grading the strength of recommendations in CPGs, for example, offers a transparent and structured process for developing recommendations [74, 75]. Thus, the application of GRADE or related systems is seen to increase.

### QI development group

Especially in the international CPGs, information on the composition and responsibilities of the QI development group is lacking. Further understanding of the interaction between the QI and CPG development groups are needed, if they work independently. In this context, cooperation and mutual feedback between these stakeholders are reasonable. For example, the QI development group might need further background information regarding recommendations, or the results of the QI development could lead to a revision of recommendations.

### Piloting and evaluation of QI

None of the QIs from German S3-CPGs were piloted or evaluated. However, this step ought to be seen as an essential element in the process of developing QIs [73, 76]. To assess the usefulness of potential QIs, information on criteria including technical feasibility, reliability, and validity is necessary. Such data can be generated only by testing the QIs in routine care [77]. Accordingly, several literature and protocols regarding the piloting and evaluation of QIs in general (not only guideline-based) are available [77–80].

### Strength and limitations of the review

The strength of our analyses is the systematic methodological approach which followed a pre-defined protocol. However, although we conducted systematic literature searches in the two main guideline databases, we may have missed CPGs not included in the databases. A further limitation of our analysis is that we probably missed information on methodological issues from further CPG providers, as we only included CPGs that matched substantively with a German S3-CPG. Furthermore, potential limitations arise from the fact that both the selection of CPGs and data extraction were performed by only one reviewer and checked by another. This pragmatic approach was chosen because of the large number of hits obtained by the diverse searches, as well as the low level of complexity regarding inclusion criteria in our study. Moreover, the data extraction is in agreement with a recent methodological guide on systematic reviews of CPGs [81].

We did not analyse the aspect of evidence underlying the QIs in the German S3- and international CPGs in depth, as we found various systems rating the quality of evidence and grading the strength of recommendations in the CPGs.

Finally, the interpretability of our results might be limited as we compared the QIs on clinical topics that were addressed in both CPGs of a CPG pair directly, rather than at the recommendation level. As noted above, although the CPG pairs addressed the same clinical topics, the recommendations varied to some extent and, in some cases, resulted in QIs that were not comparable. However, it should be noted that only about half of the QIs reported in international CPGs were based explicitly on guideline recommendations. The underlying approaches for generating such QIs were not reported in sufficient detail.

## Conclusion

The majority of QIs in German and international CPGs were not comparable. Various reasons for this are conceivable, such as methodological issues or contextual differences between countries. However, no clear reason could be deduced from the available data. Detailed information on the methodological approaches of generating QIs is lacking. More transparent reporting of the underlying methods for generating guideline-based QIs is recommended.

## Additional files

Additional file 1: German S3-CPGs which report QIs. (DOCX 27 kb)

Additional file 2: Search strategies. (DOCX 17 kb)

Additional file 3: Excluded articles. (DOCX 55 kb)

Additional file 4: Quality appraisal of included CPGs. (DOCX 35 kb)

Additional file 5: Topic-related QIs in international CPGs and German S3-CPGs and if applicable corresponding recommendations. (DOCX 238 kb)

Additional file 6: Comparison of topic-related QIs in international CPGs and German S3-CPGs. (DOCX 37 kb)

Additional file 7: **Table a.** Data-Extraction of German S3-CPGs - general and methodical aspects and **Table b.** Data-Extraction of international CPGs - general and methodical aspects. (DOCX 60 kb)

## Data Availability

All data generated or analysed during this study are included in this published article and its supplementary information files.

## Abbreviations

AWMF: German Association of the Scientific Medical societies
CPG: Clinical practice guideline
DELBI: German Instrument for Methodological Guideline Appraisal
GGPO: German Guideline Program in Oncology
G-I-N: Guidelines International Network
GoR: Grade of Recommendation
LoE: Level of Evidence
NDMG: National Program for Disease Management Guidelines
NGC: National Guideline Clearinghouse
NICE: National Institute for Health and Care Excellence
QI: Quality indicator
