# Correction to: Guideline-based quality indicators—a systematic comparison of German and international clinical practice guidelines

**DOI:** 10.1186/s13012-020-01000-3

**Published:** 2020-05-20

**Authors:** Monika Becker, Jessica Breuing, Monika Nothacker, Stefanie Deckert, Marie Brombach, Jochen Schmitt, Edmund Neugebauer, Dawid Pieper

**Affiliations:** 1 Institute for Research in Operative Medicine (IFOM), Department Evidence-based health services research, Faculty of Health, Department of Medicine, Witten/Herdecke University, Ostmerheimer Str. 200, Building 38, 51109 Cologne, Germany; 2 AWMF-Institute for Medical Knowledge, Management c/o Philipps-University Marburg, Karl-von-Frisch-Straße 1, 35043 Marburg, Germany; 3 Center for Evidence-based Healthcare, University Hospital and Medical Faculty Carl Gustav Carus, TU Dresden, Fetscherstraße 74, 01309 Dresden, Germany; 4 Brandenburg Medical School – Theodor Fontane, Fehrbelliner Str.38, 16816 Neuruppin, Germany

**Correction to: Implementation Sci (2019) 14:71**

**https://doi.org/10.1186/s13012-019-0918-y**

After publication of this article [1], the authors noticed an error with Fig. 2:

“Measurement properties are reported” should read *N* = 0/152 for the German S3 CPGs and *N* = 0/166 for the international CPGs instead of *N* = 39/152 and *N* = 39/166 respectively.

The correct Fig. 2 is show below:

**Fig. 2:**
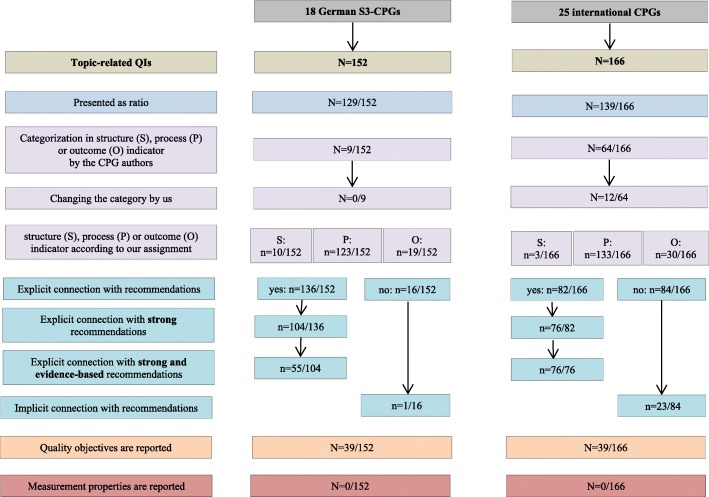
Overview of QIs
