# Supplementary material for: Guideline-based quality indicators—a systematic comparison of German and international clinical practice guidelines
Source: Implement Sci. 2019 Jul 9;14:71. doi: 10.1186/s13012-019-0918-y (PMC6617919; doi:10.1186/s13012-019-0918-y)
Supplement: Supplementary file 2 — Search strategies. (DOCX 17 kb) [file 13012_2019_918_MOESM2_ESM.docx]

Additional file 2: Search strategies

| **Topic** | **Search terms** |
| --- | --- |
| Oncology | *carcinoma OR *cancer OR oncolog* |
| Hodgkin lymphoma | hodgkin lymphoma |
| Melanoma | melanoma |
| Diabetes | diabet* |
| Back pain | back pain OR dorsalgia OR backage |
| Adiposity | obesity OR overweight OR adiposity |
| Tiredness | tiredness OR fatigue OR lassitude OR weariness |
| Carpal tunnel syndrome | carpal tunnel syndrome |
| Bipolar disorder | bipolar disorder |
| Community-acquired pneumonia | pneumonia |
| Epidural spinal cord stimulation | spinal cord stimulation |
| Long-term opioid-use in non-cancer pain | opioid* OR chronic pain OR non-cancer pain |
| Perioperative hypothermia | hypothermia |
| Peripheral nerve injuries | nerve lesion or nerve injury |
| Hysterectomy for benigne diseases | cervi* OR endometri* OR hysterectom* or uter* OR myom* OR adenomyosis OR descensus OR menstru* |
| Rational antibiotic use in hospitals | antibiotic* |
| Venous thromboembolism | thrombotic embolism OR thromboembolism OR VTE |
| Delirium, analgesia and sedation in intensiv care medicine | intensive care OR critical* ill* |
| Alcohol related disorders | alcohol* or substance |
