# Supplementary material for: Guideline-based quality indicators—a systematic comparison of German and international clinical practice guidelines
Source: Implement Sci. 2019 Jul 9;14:71. doi: 10.1186/s13012-019-0918-y (PMC6617919; doi:10.1186/s13012-019-0918-y)
Supplement: Supplementary file 3 — Excluded articles. (DOCX 55 kb) [file 13012_2019_918_MOESM3_ESM.docx]

**Additional file 3: Excluded articles**

**The document is no guideline (n=4)**

1. Aldasoro Unamuno E, Mahtani Chugani V, Sáenz de Ormijana Hernández A, Fernández Vega E, González Castro I, Martín Fernández R, Garagalza Arrizabalaga A, Esnaola Suquía S, Rico Iturrioz R. Necesidades en cuidados paliativos de las enfermedades no oncológicas. Un estudio cualitativo desde la perspectiva de profesionales, pacientes y personas cuidadoras. Madrid: Plan Nacional para el SNS del MSC. Servicio de Evaluación de Tecnologías Sanitarias del País Vasco (Osteba). 2012. URL: www.osakidetza.euskadi.eus/contenidos/informacion/2012_osteba_publicacion/es_def/adjuntos/e_12_02%20Cuidados%20paliativos.pdf. Assessed 13 June 2017.

2. Haute Autorité De Santé (HAS). Dépistage et prévention du cancer colorectal. 2013. URL: https://www.has-sante.fr/portail/upload/docs/application/pdf/2013-08/referentieleps_format2clic_kc_colon-vfinale_2013-08-30_vf_mel_2013-08-30_12-18-6_653.pdf. Assessed 13 June 2017.

3. Haute Autorité De Santé (HAS). Dépistage et prévention du cancer du sein. 2015. URL: https://www.has-sante.fr/portail/jcms/c_2024559/fr/depistage-et-prevention-du-cancer-du-sein. Assessed 13 June 2017.

4. Sarriugarte G, Sanz-Guinea A, Mar J, Antoñanzas F, Nuño R, Orue-Etxebarria B, Rueda JR. Estudio de costes del programa de detección precoz del cáncer de mama de la Comunidad Autónoma del País Vasco. Revisión sistemática de estudios de evaluación económica del cribado de cáncer de mama. Investigación Comisionada. Vitoria-Gasteiz. Departamento de Sanidad y Consumo, Gobierno Vasco. 2012. URL: http://www.osakidetza.euskadi.eus/contenidos/informacion/2012_osteba_publicacion/es_def/adjuntos/D-12-03_WEB%20_estudio%20de%20costes%20PDPCM.pdf. Assessed 13 June 2017.

**No qualityindicators are reported (n=243)**

5. Massad LS, Einstein MH, Huh WK, Katki HA, Kinney WK, Schiffman M, Solomon D, Wentzensen N, Lawson HW: 2012 updated consensus guidelines for the management of abnormal cervical cancer screening tests and cancer precursors. *Journal of lower genital tract disease* 2013, 17(5 Suppl 1):S1-s27.

6. Jensen MD, Ryan DH, Apovian CM, Ard JD, Comuzzie AG, Donato KA, Hu FB, Hubbard VS, Jakicic JM, Kushner RF *et al*: 2013 AHA/ACC/TOS guideline for the management of overweight and obesity in adults: a report of the American College of Cardiology/American Heart Association Task Force on Practice Guidelines and The Obesity Society. *Journal of the American College of Cardiology* 2014, 63(25 Pt B):2985-3023.

7. Singh S, Best C, Dunn S, Leyland N, Wolfman WL: Abnormal uterine bleeding in pre-menopausal women. *Journal of obstetrics and gynaecology Canada : JOGC = Journal d'obstetrique et gynecologie du Canada : JOGC* 2013, 35(5):473-475.

8. Academy of Nutrition and Diatetics. Adult Weight Management. 2014. URL: https://www.andeal.org/topic.cfm?cat=2798. Assessed 22 March 2017. In*.*

9. Academy of Nutrition and Diatetics. Pediatric weight management evidence-based nutrition practice guideline. 2015. URL: https://www.andeal.org/topic.cfm?menu=5296&cat=5632. Assessed 22 Macrh 2017. In*.*

10. Academy of Nutrition and Dietetics. Critical Illness (CI) Evidence-Based Nutrition Practice Guideline. 2012. URL: https://www.andeal.org/topic.cfm?menu=2799&cat=4800. Assessed 19 Sep 2017. In*.*

11. Correa C, Harris EE, Leonardi MC, Smith BD, Taghian AG, Thompson AM, White J, Harris JR: Accelerated Partial Breast Irradiation: Executive summary for the update of an ASTRO Evidence-Based Consensus Statement. *Practical radiation oncology* 2017, 7(2):73-79.

12. Chen RC, Rumble RB, Loblaw DA, Finelli A, Ehdaie B, Cooperberg MR, Morgan SC, Tyldesley S, Haluschak JJ, Tan W *et al*: Active Surveillance for the Management of Localized Prostate Cancer (Cancer Care Ontario Guideline): American Society of Clinical Oncology Clinical Practice Guideline Endorsement. *Journal of clinical oncology : official journal of the American Society of Clinical Oncology* 2016, 34(18):2182-2190.

13. Freedland SJ, Rumble RB, Finelli A, Chen RC, Slovin S, Stein MN, Mendelson DS, Wackett C, Sandler HM: Adjuvant and salvage radiotherapy after prostatectomy: American Society of Clinical Oncology clinical practice guideline endorsement. *Journal of clinical oncology : official journal of the American Society of Clinical Oncology* 2014, 32(34):3892-3898.

14. Thompson IM, Valicenti RK, Albertsen P, Davis BJ, Goldenberg SL, Hahn C, Klein E, Michalski J, Roach M, Sartor O *et al*: Adjuvant and salvage radiotherapy after prostatectomy: AUA/ASTRO Guideline. *The Journal of urology* 2013, 190(2):441-449.

15. Alberta Health Services. Adjuvant interferon for malignant melanoma. 2014. URL: http://www.albertahealthservices.ca/assets/info/hp/cancer/if-hp-cancer-guide-cu002-adjuvant-interferon.pdf. Assessed 15 March 2017.

16. Alberta Health Services. Adjuvant radiation therapy for invasive breast cancer. 2015. URL: http://www.albertahealthservices.ca/assets/info/hp/cancer/if-hp-cancer-guide-br005-adjuvant-rt-invasive-breast.pdf. Assessed 03 April 2017.

17. Alberta Health Services. Bone health in patients with breast cancer. 2012. URL: http://www.albertahealthservices.ca/assets/info/hp/cancer/if-hp-cancer-guide-br010-bone-modifying-agents.pdf. Assessed 24 March 2017.

18. Alberta Health Services. Epithelial ovarian, fallopian tube, and primary peritoneal cancer. 2013. URL: http://www.albertahealthservices.ca/assets/info/hp/cancer/if-hp-cancer-guide-gyne005-epithelialovarian.pdf. Assessed 24 April 2017.

19. Alberta Health Services. Follow-up care for early-stage breast cancer. 2015. URL: http://www.albertahealthservices.ca/assets/info/hp/cancer/if-hp-cancer-guide-br013-early-stage-follow-up.pdf. Assessed 24 March 2017.

20. Alberta Health Services. Management of patients with early esophageal cancer, dysplastic and non-dysplastic Barrett's esophagus. 2014. URL: http://www.albertahealthservices.ca/assets/info/hp/cancer/if-hp-cancer-guide-gi011-barretts-esophagus.pdf. Assessed 21 March 2017.

21. Alberta Health Services. Management of resectable stage IV primary cutaneous melanoma without nodal disease. 2013. URL: http://www.albertahealthservices.ca/assets/info/hp/cancer/if-hp-cancer-guide-cu009-resectable-stage-IV-disease.pdf. Assessed 15 March 2017.

22. Alberta Health Services. Optimal excision margins for primary cutaneous melanoma. 2013. URL: http://www.albertahealthservices.ca/assets/info/hp/cancer/if-hp-cancer-guide-cu010-optimal-margins.pdf. Assessed 15 March 2017.

23. Alberta Health Services. Optimal use of taxanes in metastatic breast cancer (MBC). 2013. URL: http://www.albertahealthservices.ca/assets/info/hp/cancer/if-hp-cancer-guide-br001-opt-use-taxanes.pdf. Assessed 24 March 2017.

24. Alberta Health Services. Ovarian germ cell tumours. 2013. URL: http://www.albertahealthservices.ca/assets/info/hp/cancer/if-hp-cancer-guide-gyne001-germ-cell.pdf. Assessed 20 March 2017.

25. Alberta Health Services. Preoperative and pretreatment investigations for malignant melanoma. 2013. URL: http://www.albertahealthservices.ca/assets/info/hp/cancer/if-hp-cancer-guide-cu007-preop-pretreat-invest.pdf. Assessed 15 March 2017.

26. Alberta Health Services. Prophylaxis and treatment of venous thromboembolism in patients undergoing treatment for solid tumours. 2014. URL: http://www.albertahealthservices.ca/info/cancerguidelines.aspx. Assessed 17 March 2017.

27. Alberta Health Services. Referral and follow-up surveillance of cutaneous melanoma. 2013. URL: http://www.albertahealthservices.ca/assets/info/hp/cancer/if-hp-cancer-guide-cu001-followup-surveillance.pdf. Assessed 15 March 2017.

28. Alberta Health Services. Staging investigations for asymptomatic and newly diagnosed breast cancer. 2012. URL: http://www.albertahealthservices.ca/assets/info/hp/cancer/if-hp-cancer-guide-br012-staging-investigations.pdf. Assessed 24 March 2017.

29. Alberta Health Services. Systemic therapy for unresectable stage III or metastatic cutaneous melanoma. 2015. URL: http://www.albertahealthservices.ca/assets/info/hp/cancer/if-hp-cancer-guide-cu012-systemic-therapy.pdf. Assessed 15 March 2017.

30. American Academy of Orthopaedic Surgeons. Management of Carpal Tunnel Syndrome Evidence-Based Clinical Practice Guideline. Feb 2016. www.aaos.org/ctsguideline. Assessed 14 March 2017.

31. Saslow D, Solomon D, Lawson HW, Killackey M, Kulasingam SL, Cain J, Garcia FA, Moriarty AT, Waxman AG, Wilbur DC *et al*: American Cancer Society, American Society for Colposcopy and Cervical Pathology, and American Society for Clinical Pathology screening guidelines for the prevention and early detection of cervical cancer. *CA: a cancer journal for clinicians* 2012, 62(3):147-172.

32. Runowicz CD, Leach CR, Henry NL, Henry KS, Mackey HT, Cowens-Alvarado RL, Cannady RS, Pratt-Chapman ML, Edge SB, Jacobs LA *et al*: American Cancer Society/American Society of Clinical Oncology Breast Cancer Survivorship Care Guideline. *Journal of clinical oncology : official journal of the American Society of Clinical Oncology* 2016, 34(6):611-635.

33. American College of Radiology. ACR Appropriateness Criteria: pretreatment planning of invasive cancer of the cervix. 2015. URL: https://acsearch.acr.org/docs/69461/Narrative/. Assessed 21 March 2017.

34. American College of Radiology. ACR Appropriateness Criteria: advanced cervical cancer. 2012. https://acsearch.acr.org/docs/70544/Narrative/. Assessed 22 march 2017.

35. American College of Radiology. ACR Appropriateness Criteria: breast cancer screening. 2016. URL: https://acsearch.acr.org/docs/70910/Narrative/. Assessed 24 March 2017.

36. American College of Radiology. ACR Appropriateness Criteria: chronic back pain: suspected sacroiliitis/spondyloarthropathy. 2016. URL: https://acsearch.acr.org/docs/3094107/Narrative/. Assessed 03 March 2017.

37. American College of Radiology. ACR Appropriateness Criteria: colorectal cancer screening. 2013. URL: https://acsearch.acr.org/docs/69469/Narrative/. Assessed 23 March 2017.

38. American College of Radiology. ACR Appropriateness Criteria: definitive therapy for early stage cervical cancer. 2012. URL: https://acsearch.acr.org/docs/70908/Narrative/. Assessed 22 March 2017.

39. American College of Radiology. ACR Appropriateness Criteria: ductal carcinoma in situ. 2014. URL: https://acsearch.acr.org/docs/69386/Narrative/. Assessed 03 March 2017.

40. American College of Radiology. ACR Appropriateness Criteria: efinitive external-beam irradiation in stage T1 and T2 prostate cancer. 2013. URL: https://acsearch.acr.org/docs/69350/Narrative/. Assessed 22 March 2017.

41. American College of Radiology. ACR Appropriateness Criteria: follow-up of Hodgkin lymphoma. 2014. URL: https://acsearch.acr.org/docs/69388/Narrative/. Assessed 22 March 2017. In*.*

42. American College of Radiology. ACR Appropriateness Criteria: high-dose-rate brachytherapy for prostate cancer. 2013. URL: https://acsearch.acr.org/docs/3082863/Narrative/. Assessed 22 March 2017.

43. American College of Radiology. ACR Appropriateness Criteria: Hodgkin lymphoma—unfavorable clinical stage I and II. 2015. URL: https://acsearch.acr.org/docs/69389/Narrative/. Assessed 22 March 2017.

44. American College of Radiology. ACR Appropriateness Criteria: local-regional recurrence (LRR) and salvage surgery—breast cancer. 2013. URL: https://acsearch.acr.org/docs/69387/Narrative/. Assessed 24 March 2017.

45. American College of Radiology. ACR Appropriateness Criteria: Locoregional Therapy for Resectable Oropharyngeal Squamous Cell Carcinomas. 2015. URL: https://acsearch.acr.org/docs/69505/Narrative/. Assessed 16 March 2017.

46. American College of Radiology. ACR Appropriateness Criteria: low back pain. 2015. URL: https://acsearch.acr.org/docs/69483/Narrative/. Assessed 03 March 2017.

47. American College of Radiology. ACR Appropriateness Criteria: ovarian cancer screening. 2012. URL: https://acsearch.acr.org/docs/69463/Narrative/. Assessed 17 March 2017.

48. American College of Radiology. ACR Appropriateness Criteria: Postmastectomy radiotherapy. 2012. URL: https://acsearch.acr.org/docs/69347/Narrative/. Assessed 03 March 2017.

49. American College of Radiology. ACR Appropriateness Criteria: postradical prostatectomy irradiation in prostate cancer. 2014. URL: https://acsearch.acr.org/docs/69400/Narrative/. Assessed 23 March 2017.

50. American College of Radiology. ACR Appropriateness Criteria: post-treatment follow-up of renal cell carcinoma. 2013. URL: https://acsearch.acr.org/docs/69365/Narrative/. Assessed 16 March 2017.

51. American College of Radiology. ACR Appropriateness Criteria: pretreatment staging of colorectal cancer. 2016. URL: https://acsearch.acr.org/docs/69339/Narrative/. Assessed 23 March 2017.

52. American College of Radiology. ACR Appropriateness Criteria: prostate cancer—pretreatment detection, staging and surveillance. 2012. URL: https://acsearch.acr.org/docs/69371/Narrative/. Assessed 23 March 2017.

53. American College of Radiology. ACR Appropriateness Criteria: radiologic management of hepatic malignancy. 2015. URL: https://acsearch.acr.org/docs/69379/Narrative/. Assessed 16 March 2017.

54. American College of Radiology. ACR Appropriateness Criteria: Renal Cell Carcinoma Staging. 2015: URL: https://acsearch.acr.org/docs/69372/Narrative/. Assessed 16 March 2017.

55. American College of Radiology. ACR Appropriateness Criteria: resectable stomach cancer. 2014. URL: https://acsearch.acr.org/docs/3091668/Narrative/. Assessed 16 March 2017.

56. American College of Radiology. ACR Appropriateness Criteria: role of adjuvant therapy in the management of early stage cervical cancer. 2014. URL: https://acsearch.acr.org/docs/70543/Narrative/. Assessed 22 March 2017.

57. American College of Radiology. ACR Appropriateness Criteria: stage I breast cancer: initial workup and surveillance for local recurrence and distant metastases in asymptomatic women. 2016. URL: https://acsearch.acr.org/docs/69496/Narrative/. Assessed 24 March 2017.

58. American College of Radiology. ACR Appropriateness Criteria: staging and follow-up of ovarian cancer. 2012. URL: https://acsearch.acr.org/docs/69378/Narrative/. Assessed 20 March 2017.

59. American College of Radiology. ACR Appropriateness Criteria: Suspected Lower-Extremity Deep Vein Thrombosis. 2013. URL: https://acsearch.acr.org/docs/69416/Narrative/. Assessed 17 March 2017.

60. American Geriatrics Society abstracted clinical practice guideline for postoperative delirium in older adults. *Journal of the American Geriatrics Society* 2015, 63(1):142-150.

61. American Osteopathic Association Guidelines for Osteopathic Manipulative Treatment (OMT) for Patients With Low Back Pain. *The Journal of the American Osteopathic Association* 2016, 116(8):536-549.

62. Manchikanti L, Abdi S, Atluri S, Balog CC, Benyamin RM, Boswell MV, Brown KR, Bruel BM, Bryce DA, Burks PA *et al*: American Society of Interventional Pain Physicians (ASIPP) guidelines for responsible opioid prescribing in chronic non-cancer pain: Part 2--guidance. *Pain physician* 2012, 15(3 Suppl):S67-116.

63. Lansberg MG, O'Donnell MJ, Khatri P, Lang ES, Nguyen-Huynh MN, Schwartz NE, Sonnenberg FA, Schulman S, Vandvik PO, Spencer FA *et al*: Antithrombotic and thrombolytic therapy for ischemic stroke: Antithrombotic Therapy and Prevention of Thrombosis, 9th ed: American College of Chest Physicians Evidence-Based Clinical Practice Guidelines. *Chest* 2012, 141(2 Suppl):e601S-e636S.

64. Whitlock RP, Sun JC, Fremes SE, Rubens FD, Teoh KH: Antithrombotic and thrombolytic therapy for valvular disease: Antithrombotic Therapy and Prevention of Thrombosis, 9th ed: American College of Chest Physicians Evidence-Based Clinical Practice Guidelines. *Chest* 2012, 141(2 Suppl):e576S-e600S.

65. You JJ, Singer DE, Howard PA, Lane DA, Eckman MH, Fang MC, Hylek EM, Schulman S, Go AS, Hughes M *et al*: Antithrombotic therapy for atrial fibrillation: Antithrombotic Therapy and Prevention of Thrombosis, 9th ed: American College of Chest Physicians Evidence-Based Clinical Practice Guidelines. *Chest* 2012, 141(2 Suppl):e531S-e575S.

66. Kearon C, Akl EA, Comerota AJ, Prandoni P, Bounameaux H, Goldhaber SZ, Nelson ME, Wells PS, Gould MK, Dentali F *et al*: Antithrombotic therapy for VTE disease: Antithrombotic Therapy and Prevention of Thrombosis, 9th ed: American College of Chest Physicians Evidence-Based Clinical Practice Guidelines. *Chest* 2012, 141(2 Suppl):e419S-e496S.

67. Monagle P, Chan AKC, Goldenberg NA, Ichord RN, Journeycake JM, Nowak-Gottl U, Vesely SK: Antithrombotic therapy in neonates and children: Antithrombotic Therapy and Prevention of Thrombosis, 9th ed: American College of Chest Physicians Evidence-Based Clinical Practice Guidelines. *Chest* 2012, 141(2 Suppl):e737S-e801S.

68. Alonso-Coello P, Bellmunt S, McGorrian C, Anand SS, Guzman R, Criqui MH, Akl EA, Vandvik PO, Lansberg MG, Guyatt GH *et al*: Antithrombotic therapy in peripheral artery disease: Antithrombotic Therapy and Prevention of Thrombosis, 9th ed: American College of Chest Physicians Evidence-Based Clinical Practice Guidelines. *Chest* 2012, 141(2 Suppl):e669S-e690S.

69. Whiteman DC, Appleyard M, Bahin FF, Bobryshev YV, Bourke MJ, Brown I, Chung A, Clouston A, Dickins E, Emery J *et al*: Australian clinical practice guidelines for the diagnosis and management of Barrett's esophagus and early esophageal adenocarcinoma. *Journal of gastroenterology and hepatology* 2015, 30(5):804-820.

70. Benahmed N, Robays J, Stordeur S, Gil T, Joniau S, Lumen N, Renard L, Rorive S, Schrijvers D, Tombal B, Van den Eynden B, Villeirs G, Rottey S. Renal cancer in adults: diagnosis, treatment and follow-up – Summary. Good Clinical Practice (GCP) Brussels: Belgian Health Care Knowledge Centre (KCE). KCE Reports 253Cs. D/2015/10.273/85. 2016. URL: https://kce.fgov.be/fr/node/420. Assessed 26 March 2017.

71. Brackstone M, Fletcher GG, Dayes IS, Madarnas Y, SenGupta SK, Verma S. Locoregional therapy of locally advanced breast cancer (LABC). Toronto (ON): Cancer Care Ontario; Program in Evidence-Based Care Evidence-Based Series No.: 1-19. 2014. URL: https://www.cancercare.on.ca/common/pages/UserFile.aspx?fileId=334821. Assessed 24 March 2017.

72. Khatcheressian JL, Hurley P, Bantug E, Esserman LJ, Grunfeld E, Halberg F, Hantel A, Henry NL, Muss HB, Smith TJ *et al*: Breast cancer follow-up and management after primary treatment: American Society of Clinical Oncology clinical practice guideline update. *Journal of clinical oncology : official journal of the American Society of Clinical Oncology* 2013, 31(7):961-965.

73. Oeffinger KC, Fontham ET, Etzioni R, Herzig A, Michaelson JS, Shih YC, Walter LC, Church TR, Flowers CR, LaMonte SJ *et al*: Breast Cancer Screening for Women at Average Risk: 2015 Guideline Update From the American Cancer Society. *Jama* 2015, 314(15):1599-1614.

74. Shea-Budgell M, Quan ML, Mehling B, Temple-Oberle C: Breast reconstruction following prophylactic or therapeutic mastectomy for breast cancer: Recommendations from an evidence-based provincial guideline. *Plastic surgery (Oakville, Ont)* 2014, 22(2):103-111.

75. British Columbia Medical Association. Follow-up of colorectal polyps or cancer. 2013. URL: http://www2.gov.bc.ca/assets/gov/health/practitioner-pro/bc-guidelines/colorectal_followup.pdf. Assessed 23 March 2017.

76. Cancer Australia. Clinical guidance for responding to suffering in adults with cancer. 2014. URL: https://canceraustralia.gov.au/publications-and-resources/clinical-practice-guidelines. Assessed 26 March 2017.

77. Cancer Australia. Clinical guidance for the management of lobular carcinoma in situ. Clinical guidance for the management of lobular carcinoma in situ. 2016. URL: https://canceraustralia.gov.au/publications-and-resources/clinical-practice-guidelines. Assessed 03 April 2017.

78. Cancer Australia. CNS metastases in women with secondary breast cancer. Recommendations for the management of central nervous system (CNS) metastases in women with secondary breast cancer. 2014. URL: https://canceraustralia.gov.au/publications-and-resources/clinical-practice-guidelines. Assessed 03 April 2017.

79. Cancer Australia. Follow up of women with epithelial ovarian cancer. 2012. URL: https://canceraustralia.gov.au/publications-and-resources/clinical-practice-guidelines. Assessed 26 March 2017*.*

80. Cancer Australia. Hypofractionated radiotherapy for early (operable) breast cancer. Recommendations for use of Hypofractionated radiotherapy for early (operable) breast cancer. 2015. URL: https://canceraustralia.gov.au/publications-and-resources/clinical-practice-guidelines. Assessed 03 April 2017.

81. Cancer Australia. Recommendations for the management of early breast cancer in women with an identified BRCA1 or BRCA2 gene mutation or at high risk of a gene mutation. 2014. URL: https://canceraustralia.gov.au/publications-and-resources/clinical-practice-guidelines. Assessed 03 April 2017.

82. Cancer Australia. Recommendations for the use of first-line chemotherapy for the treatment of women with epithelial ovarian cancer. 2014. URL: https://canceraustralia.gov.au/publications-and-resources/clinical-practice-guidelines. Assessed 26 March 2017.

83. Cookson MS, Lowrance WT, Murad MH, Kibel AS: Castration-resistant prostate cancer: AUA guideline amendment. *The Journal of urology* 2015, 193(2):491-499.

84. Centers for Disease Control and Prevention. CDC Guideline for Prescribing Opioids for Chronic Pain — United States, 2016. MMWR Recomm Rep 2016. URL: https://www.cdc.gov/drugoverdose/prescribing/guideline.html. Assessed 13 June 2017.

85. Chang MC, Souter LH, Kamel-Reid S, Rutherford M, Bedard P, Trudeau M, et al. Clinical utility of multigene profiling assays in early-stage breast cancer. Toronto (ON): Cancer Care Ontario; Year Month Day. Program in Evidence-Based Care Recommendation Report No.: MOAC-4. 2016. URL: https://www.cancercare.on.ca/common/pages/UserFile.aspx?fileId=362126. Assessed 24 March 2017.

86. Partridge AH, Rumble RB, Carey LA, Come SE, Davidson NE, Di Leo A, Gralow J, Hortobagyi GN, Moy B, Yee D *et al*: Chemotherapy and targeted therapy for women with human epidermal growth factor receptor 2-negative (or unknown) advanced breast cancer: American Society of Clinical Oncology Clinical Practice Guideline. *Journal of clinical oncology : official journal of the American Society of Clinical Oncology* 2014, 32(29):3307-3329.

87. Godwin SA, Burton JH, Gerardo CJ, Hatten BW, Mace SE, Silvers SM, Fesmire FM: Clinical policy: procedural sedation and analgesia in the emergency department. *Annals of emergency medicine* 2014, 63(2):247-258.e218.

88. Huang ET, Mansouri J, Murad MH, Joseph WS, Strauss MB, Tettelbach W, Worth ER: A clinical practice guideline for the use of hyperbaric oxygen therapy in the treatment of diabetic foot ulcers. *Undersea & hyperbaric medicine : journal of the Undersea and Hyperbaric Medical Society, Inc* 2015, 42(3):205-247.

89. Clinical Practice Guideline on management of patients with diabetes and chronic kidney disease stage 3b or higher (eGFR <45 mL/min). *Nephrology, dialysis, transplantation : official publication of the European Dialysis and Transplant Association - European Renal Association* 2015, 30 Suppl 2:ii1-142.

90. Barr J, Fraser GL, Puntillo K, Ely EW, Gelinas C, Dasta JF, Davidson JE, Devlin JW, Kress JP, Joffe AM *et al*: Clinical practice guidelines for the management of pain, agitation, and delirium in adult patients in the intensive care unit. *Critical care medicine* 2013, 41(1):263-306.

91. Greenlee H, Balneaves LG, Carlson LE, Cohen M, Deng G, Hershman D, Mumber M, Perlmutter J, Seely D, Sen A *et al*: Clinical practice guidelines on the use of integrative therapies as supportive care in patients treated for breast cancer. *Journal of the National Cancer Institute Monographs* 2014, 2014(50):346-358.

92. The College of American Pathologists and American Society for Colposcopy and Cervical Pathology. The Lower Anogenital Squamous Terminology Standardization Project for HPV-associated lesions: background and consensus recommendations from the College of American Pathologists and the American Society for Colposcopy and Cervical Pathology. 2012. URL: http://download.lww.com/wolterskluwer_vitalstream_com/PermaLink/LGT/A/LGT_2012_06_11_DARRAGH_200570_SDC1.pdf. Assessed 20 March 2017.

93. Kahi CJ, Boland CR, Dominitz JA, Giardiello FM, Johnson DA, Kaltenbach T, Lieberman D, Levin TR, Robertson DJ, Rex DK: Colonoscopy surveillance after colorectal cancer resection: recommendations of the US multi-society task force on colorectal cancer. *Gastrointestinal endoscopy* 2016, 83(3):489-498.e410.

94. Colorado Division of Workers' Compensation. Low Back Pain Medical Treatment Guidelines. 2014. URL: https://www.colorado.gov/pacific/cdle/medical-treatment-guidelines. Assessed 08 Feb 2017. In*.*

95. Bentley J: Colposcopic management of abnormal cervical cytology and histology. *Journal of obstetrics and gynaecology Canada : JOGC = Journal d'obstetrique et gynecologie du Canada : JOGC* 2012, 34(12):1188-1202.

96. Small W, Jr., Thomas TO, Alvarado M, Baum M, Bulsara M, Diaz R, Donnelly E, Feldman S, Grobmyer S, Hoefer R *et al*: Commentary on "Accelerated partial breast irradiation consensus statement: Update of an ASTRO Evidence-Based Consensus Statement". *Practical radiation oncology* 2017, 7(3):e159-e163.

97. Dhall SS, Hadley MN, Aarabi B, Gelb DE, Hurlbert RJ, Rozzelle CJ, Ryken TC, Theodore N, Walters BC: Deep venous thrombosis and thromboembolism in patients with cervical spinal cord injuries. *Neurosurgery* 2013, 72 Suppl 2:244-254.

98. Department of Veterans Affairs Department of Defense. Clinical Practice Guideline for Screening and Management of Overweight and Obesity. 2014. URL: https://www.healthquality.va.gov/guidelines/cd/obesity/. Assessed 22 Feb 2017. In*.*

99. Department of Veterans Affairs Department of Defense. VA/DoD Clinical Practice Guideline for Assessment and Management of Patients at Risk for Suicide. 2013. URL: https://www.healthquality.va.gov/guidelines/mh/srb/. Assessed 14 March 2017. In*.*

100. Department of Veterans Affairs Department of Defense. VA/DoD Clinical Practice Guideline for the Management of Substance Use Disorders. 2015. URL: https://www.healthquality.va.gov/guidelines/mh/sud/. Assessed 20 March 2017. In*.*

101. Blumer I, Hadar E, Hadden DR, Jovanovic L, Mestman JH, Murad MH, Yogev Y: Diabetes and pregnancy: an endocrine society clinical practice guideline. *The Journal of clinical endocrinology and metabolism* 2013, 98(11):4227-4249.

102. Diabetes management at camps for children with diabetes. *Diabetes care* 2012, 35 Suppl 1:S72-75.

103. Legro RS, Arslanian SA, Ehrmann DA, Hoeger KM, Murad MH, Pasquali R, Welt CK: Diagnosis and treatment of polycystic ovary syndrome: an Endocrine Society clinical practice guideline. *The Journal of clinical endocrinology and metabolism* 2013, 98(12):4565-4592.

104. Bates SM, Jaeschke R, Stevens SM, Goodacre S, Wells PS, Stevenson MD, Kearon C, Schunemann HJ, Crowther M, Pauker SG *et al*: Diagnosis of DVT: Antithrombotic Therapy and Prevention of Thrombosis, 9th ed: American College of Chest Physicians Evidence-Based Clinical Practice Guidelines. *Chest* 2012, 141(2 Suppl):e351S-e418S.

105. Carter HB, Albertsen PC, Barry MJ, Etzioni R, Freedland SJ, Greene KL, Holmberg L, Kantoff P, Konety BR, Murad MH *et al*: Early detection of prostate cancer: AUA Guideline. *The Journal of urology* 2013, 190(2):419-426.

106. Meissner MH, Gloviczki P, Comerota AJ, Dalsing MC, Eklof BG, Gillespie DL, Lohr JM, McLafferty RB, Murad MH, Padberg F *et al*: Early thrombus removal strategies for acute deep venous thrombosis: clinical practice guidelines of the Society for Vascular Surgery and the American Venous Forum. *Journal of vascular surgery* 2012, 55(5):1449-1462.

107. Eisen A, Fletcher GG, Gandhi S, Mates M, Freedman OC, Dent SF, et al. Optimal systematic therapy for early female breast cancer. Toronto (ON): Cancer Care Ontario; Program in Evidence-based Care Evidence-Based Series No.: 1−21. 2014. URL: https://www.cancercare.on.ca/common/pages/UserFile.aspx?fileId=334825. Assessed 24 March 2017

108. Rugo HS, Rumble RB, Macrae E, Barton DL, Connolly HK, Dickler MN, Fallowfield L, Fowble B, Ingle JN, Jahanzeb M *et al*: Endocrine Therapy for Hormone Receptor-Positive Metastatic Breast Cancer: American Society of Clinical Oncology Guideline. *Journal of clinical oncology : official journal of the American Society of Clinical Oncology* 2016, 34(25):3069-3103.

109. Cartwright MS, Hobson-Webb LD, Boon AJ, Alter KE, Hunt CH, Flores VH, Werner RA, Shook SJ, Thomas TD, Primack SJ *et al*: Evidence-based guideline: neuromuscular ultrasound for the diagnosis of carpal tunnel syndrome. *Muscle & nerve* 2012, 46(2):287-293.

110. Holbrook A, Schulman S, Witt DM, Vandvik PO, Fish J, Kovacs MJ, Svensson PJ, Veenstra DL, Crowther M, Guyatt GH: Evidence-based management of anticoagulant therapy: Antithrombotic Therapy and Prevention of Thrombosis, 9th ed: American College of Chest Physicians Evidence-Based Clinical Practice Guidelines. *Chest* 2012, 141(2 Suppl):e152S-e184S.

111. Allegra CJ, Rumble RB, Hamilton SR, Mangu PB, Roach N, Hantel A, Schilsky RL: Extended RAS Gene Mutation Testing in Metastatic Colorectal Carcinoma to Predict Response to Anti-Epidermal Growth Factor Receptor Monoclonal Antibody Therapy: American Society of Clinical Oncology Provisional Clinical Opinion Update 2015. *Journal of clinical oncology : official journal of the American Society of Clinical Oncology* 2016, 34(2):179-185.

112. Meyerhardt JA, Mangu PB, Flynn PJ, Korde L, Loprinzi CL, Minsky BD, Petrelli NJ, Ryan K, Schrag DH, Wong SL *et al*: Follow-up care, surveillance protocol, and secondary prevention measures for survivors of colorectal cancer: American Society of Clinical Oncology clinical practice guideline endorsement. *Journal of clinical oncology : official journal of the American Society of Clinical Oncology* 2013, 31(35):4465-4470.

113. Gallinger S, Biagi JJ, Fletcher GG, Nhan, C, Ruo L, McLeod RS; Expert G. The role of liver resection in colorectal cancer metastases. Toronto (ON): Cancer Care Ontario; Program in Evidence-based Care Evidence-based Series No.: 17-7. 2012. URL: https://www.cancercare.on.ca/common/pages/UserFile.aspx?fileId=154968. Assessed 23 March 2017.

114. Grégoire V, Leroy R, Heus P, van de Wetering F, Hooft L, Scholten R, Verleye L, Carp L, Clement P, Deron P, Goffin K, Hamoir M, Hauben E, Hendrickx K, Hermans R, Kunz S, Lenssen O, Nuyts S, Van Laer C, Vermorken J, Appermont E, De Prins A, Hebbelinck E, Hommez G, Vandenbruaene C, Vanhalewyck E, Vlayen J. Oral cavity cancer: diagnosis, treatment and follow-up. Good Clinical Practice (GCP) Brussels: Belgian Health Care Knowledge Centre (KCE). KCE Reports 227. D/2013/10.273/58. 2014. URL: https://kce.fgov.be/sites/default/files/atoms/files/KCE_227_oral%20cavity%20cancer_Report_2.pdf. Assessed 26 March 2017.

115. Collins GP, Parker AN, Pocock C, Kayani I, Sureda A, Illidge T, Ardeshna K, Linch DC, Peggs KS: Guideline on the management of primary resistant and relapsed classical Hodgkin lymphoma. *British journal of haematology* 2014, 164(1):39-52.

116. Lieberman DA, Rex DK, Winawer SJ, Giardiello FM, Johnson DA, Levin TR: Guidelines for colonoscopy surveillance after screening and polypectomy: a consensus update by the US Multi-Society Task Force on Colorectal Cancer. *Gastroenterology* 2012, 143(3):844-857.

117. Follows GA, Ardeshna KM, Barrington SF, Culligan DJ, Hoskin PJ, Linch D, Sadullah S, Williams MV, Wimperis JZ: Guidelines for the first line management of classical Hodgkin lymphoma. *British journal of haematology* 2014, 166(1):34-49.

118. Watson H, Davidson S, Keeling D: Guidelines on the diagnosis and management of heparin-induced thrombocytopenia: second edition. *British journal of haematology* 2012, 159(5):528-540.

119. Hartford Institute for Geriatric Nursing. Substance misuse and alcohol use disorders. In: Evidence-based geriatric nursing protocols for best practice. 2012. URL: https://consultgeri.org/geriatric-topics/substance-abuse#Guidelines. Assessed 20 March 2017.

120. Haute Autorité De Santé (HAS). Actualisation de la revue de la littérature d’une recommandation en santé publique sur la « Détection précoce du mélanome cutané ». 2013. URL: https://www.has-sante.fr/portail/upload/docs/application/pdf/2013-01/recommandation_actualisation_de_la_revue_de_la_litterature_dune_recommandation_en_sante_publique_sur_la_detection_precoce_du_melanome_cutane.pdf. Assessed 13 June 2017.

121. Haute Autorité De Santé (HAS). Lombalgie chronique de l’adulte et chirurgie. 2015. URL: https://www.has-sante.fr/portail/jcms/c_2615316/fr/lombalgie-chronique-de-l-adulte-et-chirurgie. Assessed 13 June 2017.

122. Haute Autorité De Santé (HAS). Lymphome de Hodgkin classique de l'adulte. Guide du parcours de soins. 2013. URL: https://www.has-sante.fr/portail/upload/docs/application/pdf/2013-10/guide_lymphome_hodgkin_web.pdf. Assessed 13 June 2017.

123. Haute Autorité De Santé (HAS). Patient avec un trouble bipolaire : repérage et prise en charge initiale en premier recours. 2015. URL: https://www.has-sante.fr/portail/jcms/c_1747465/fr/patient-avec-un-trouble-bipolaire-reperage-et-prise-en-charge-initiale-en-premier-recours. Assessed 13 June 2017. In*.*

124. Haute Autorité De Santé (HAS). Stratégie médicamenteuse du contrôle glycémique du diabète de type 2. Recommandation pour la pratique clinique. 2013. URL: https://www.has-sante.fr/portail/upload/docs/application/pdf/2013-02/10irp04_reco_diabete_type_2.pdf. Assessed 13 June 2017.

125. Bartley AN, Washington MK, Ventura CB, Ismaila N, Colasacco C, Benson AB, 3rd, Carrato A, Gulley ML, Jain D, Kakar S *et al*: HER2 Testing and Clinical Decision Making in Gastroesophageal Adenocarcinoma: Guideline From the College of American Pathologists, American Society for Clinical Pathology, and American Society of Clinical Oncology. *American journal of clinical pathology* 2016, 146(6):647-669.

126. Stoffel EM, Mangu PB, Gruber SB, Hamilton SR, Kalady MF, Lau MW, Lu KH, Roach N, Limburg PJ: Hereditary colorectal cancer syndromes: American Society of Clinical Oncology Clinical Practice Guideline endorsement of the familial risk-colorectal cancer: European Society for Medical Oncology Clinical Practice Guidelines. *Journal of clinical oncology : official journal of the American Society of Clinical Oncology* 2015, 33(2):209-217.

127. Herst J, Crump M, Baldassarre FG, MacEachern J, Sussman J, Hodgson D,et al. Management of early-stage Hodgkin Lymphoma: Guideline recommendations. Toronto (ON): Cancer Care Ontario; Program in Evidence-based Care Guideline No.: 6-20. 2015. URL: https://www.cancercare.on.ca/common/pages/UserFile.aspx?fileId=350543. Assessed 22 March 2017.

128. IKNL (Comprehensive Cancer Centre for the Netherlands). Breast cancer. 2012. URL: https://richtlijnendatabase.nl/en/richtlijn/breast_cancer/breast_cancer.html. Assessed 31 March 2017*.*

129. IKNL (Integraal Kankercentrum Nederland). Cervixcarcinoom. 2012. URL: http://www.oncoline.nl/cervixcarcinoom. Assessed 12 June 2017.

130. IKNL (Integraal Kankercentrum Nederland). Colorectaalcarcinoom. 2014. URL: http://www.oncoline.nl/colorectaalcarcinoom. Assessed 12 June 2017.

131. IKNL (Integraal Kankercentrum Nederland). Hepatocellulair carcinoom. 2013. URL: http://www.oncoline.nl/hepatocellulair-carcinoom. Assessed 12 June 2017.

132. IKNL (Integraal Kankercentrum Nederland). Mammacarcinoom. 2012. URL: http://oncoline.nl/mammacarcinoom. Assessed 12 June 2017.

133. IKNL (Integraal Kankercentrum Nederland). Melanoma Guideline 2012. URL: http://www.oncoline.nl/uploaded/docs/melanoom/201208_vertaling%20Richtlijn%20melanoom%20def.pdf. Assessed 26 March 2017.

134. IKNL (Integraal Kankercentrum Nederland). Prostaatcarcinoom. 2016. URL: http://oncoline.nl/prostaatcarcinoom. Assessed 12 June 2017.

135. Ferrell BR, Temel JS, Temin S, Alesi ER, Balboni TA, Basch EM, Firn JI, Paice JA, Peppercorn JM, Phillips T *et al*: Integration of Palliative Care Into Standard Oncology Care: American Society of Clinical Oncology Clinical Practice Guideline Update. *Journal of clinical oncology : official journal of the American Society of Clinical Oncology* 2017, 35(1):96-112.

136. International Working Group on the Diabetic Foot (IWGDF). The 2015 IWGDF Guidance documents on prevention and management of foot problems in diabetes: development of an evidence-based global consensus. 2015. URL: http://iwgdf.org/guidelines/. Assessed 08.02.2017.

137. Joanna Briggs Institute. Effectiveness of parent-centered interventions for the prevention and treatment of childhood overweight and obesity in community settings Best Practice: evidence-based information sheets for health professionals. 2014. URL: www.joannabriggs.org. Assessed 01 March 2017.

138. Joanna Briggs Institute. Interventions to meet family needs of critically ill patients in an adult intensive care unit Best Practice: evidence-based information sheets for health professionals. 2012. URL: www.joannabriggs.org. Assessed 03 March 2017.

139. Joanna Briggs Institute. The experiences of, and meaning for, women living and coping with type 2 diabetes. Best Practice: evidence-based information sheets for health professionals. 2012. URL: www.joannabriggs.org. Assessed 01 March 2017.

140. Joanna Briggs Institute. Women's Experience of Diabetes and Diabetes Management in Pregnancy, Best Practice: evidence-based information sheets for health professionals. 2014. URL: www.joannabriggs.org. Assessed 01 March 2017.

141. KDIGO 2012 Clinical Practice Guideline for the Evaluation and Management of Chronic Kidney Disease. Kidney International Supplements (2013) vol 3, issue 1. URL: http://www.kidney-international.org. Assessed 02 May 2017.

142. Le T, Kennedy EB, Dodge J, Elit L. Follow-up of patients who are clinically disease-free after treatment for fallopian tube, primary peritoneal, and epithelial ovarian cancer. Toronto (ON): Cancer Care Ontario; Program in Evidence-Based Care Guideline No.: 4-22. 2015. URL: https://www.cancercare.on.ca/common/pages/UserFile.aspx?fileId=349512. Assessed 20 March 2017.

143. Li M, Kennedy EB, Byrne N, Gerin-Lajoie C, Green E, Katz MR, et al. The management of depression in patients with cancer. Toronto (ON): Cancer Care Ontario; Program in Evidence-based Care Guideline No.: 19-4. 2015. URL: https://www.cancercare.on.ca/common/pages/UserFile.aspx?fileId=340750. Assessed 20 March 2017.

144. Balaban EP, Mangu PB, Khorana AA, Shah MA, Mukherjee S, Crane CH, Javle MM, Eads JR, Allen P, Ko AH *et al*: Locally Advanced, Unresectable Pancreatic Cancer: American Society of Clinical Oncology Clinical Practice Guideline. *Journal of clinical oncology : official journal of the American Society of Clinical Oncology* 2016, 34(22):2654-2668.

145. Delitto A, George SZ, Van Dillen LR, Whitman JM, Sowa G, Shekelle P, Denninger TR, Godges JJ: Low back pain. *The Journal of orthopaedic and sports physical therapy* 2012, 42(4):A1-57.

146. Mambourg F, Jonckheer P, Piérart J, Van Brabandt H. A national clinical practice guideline on the management of localised prostate cancer. Good Clinical Practice (GCP). Brussels: Belgian Health Care Knowledge Centre (KCE). KCE Reports 194C. D/2012/10.273/101. 2012. URL: https://kce.login.kanooh.be/en/a-national-clinical-practice-guideline-on-the-management-of-localised-prostate-cancer-part-1. Assessed 26 March 2017.

147. Mambourg F, Robays J, Gerkens S. Opsporing van borstkanker tussen 70 en 74 jaar. Good Clinical Practice (CPG). Brussel: Federaal Kenniscentrum voor de Gesundheitszorg (KCE). KCE Report 176 A. D/2012/10.273/18. 2012. URL: https://kce.fgov.be/en/breast-cancer-screening-amongst-women-aged-70-74-years-of-age. Assessed 26 March 2017.

148. Chuang LT, Temin S, Camacho R, Duenas-Gonzalez A, Feldman S, Gultekin M, Gupta V, Horton S, Jacob G, Kidd EA *et al*: Management and Care of Women With Invasive Cervical Cancer: American Society of Clinical Oncology Resource-Stratified Clinical Practice Guideline. *Journal of global oncology* 2016, 2(5):311-340.

149. Kalil AC, Metersky ML, Klompas M, Muscedere J, Sweeney DA, Palmer LB, Napolitano LM, O'Grady NP, Bartlett JG, Carratala J *et al*: Management of Adults With Hospital-acquired and Ventilator-associated Pneumonia: 2016 Clinical Practice Guidelines by the Infectious Diseases Society of America and the American Thoracic Society. *Clinical infectious diseases : an official publication of the Infectious Diseases Society of America* 2016, 63(5):e61-e111.

150. Hingorani A, LaMuraglia GM, Henke P, Meissner MH, Loretz L, Zinszer KM, Driver VR, Frykberg R, Carman TL, Marston W *et al*: The management of diabetic foot: A clinical practice guideline by the Society for Vascular Surgery in collaboration with the American Podiatric Medical Association and the Society for Vascular Medicine. *Journal of vascular surgery* 2016, 63(2 Suppl):3s-21s.

151. Copeland KC, Silverstein J, Moore KR, Prazar GE, Raymer T, Shiffman RN, Springer SC, Thaker VV, Anderson M, Spann SJ *et al*: Management of newly diagnosed type 2 Diabetes Mellitus (T2DM) in children and adolescents. *Pediatrics* 2013, 131(2):364-382.

152. Management of symptomatic vulvovaginal atrophy: 2013 position statement of The North American Menopause Society. *Menopause (New York, NY)* 2013, 20(9):888-902; quiz 903-884.

153. Moyer VA: Medications to decrease the risk for breast cancer in women: recommendations from the U.S. Preventive Services Task Force recommendation statement. *Annals of internal medicine* 2013, 159(10):698-708.

154. Members of the Colorectal Cancer Survivorship Group. Follow-up care, surveillance protocol, and secondary prevention measures for survivors of colorectal cancer. Toronto (ON): Cancer Care Ontario; Program in Evidence-based Care Evidence-Based Series No.: 26-2 Version 2. 2012. URL: https://cancercare.on.ca/common/pages/UserFile.aspx?fileId=124839. Assessed 23 March 2017.

155. Sohal DP, Mangu PB, Khorana AA, Shah MA, Philip PA, O'Reilly EM, Uronis HE, Ramanathan RK, Crane CH, Engebretson A *et al*: Metastatic Pancreatic Cancer: American Society of Clinical Oncology Clinical Practice Guideline. *Journal of clinical oncology : official journal of the American Society of Clinical Oncology* 2016, 34(23):2784-2796.

156. Sepulveda AR, Hamilton SR, Allegra CJ, Grody W, Cushman-Vokoun AM, Funkhouser WK, Kopetz SE, Lieu C, Lindor NM, Minsky BD *et al*: Molecular Biomarkers for the Evaluation of Colorectal Cancer: Guideline From the American Society for Clinical Pathology, College of American Pathologists, Association for Molecular Pathology, and the American Society of Clinical Oncology. *Journal of clinical oncology : official journal of the American Society of Clinical Oncology* 2017, 35(13):1453-1486.

157. National Health and Medical Research Council. Clinical practice guidelines for the management of overweight and obesity in adults, adolescents and children in Australia. Melbourne: National Health and Medical Research Council. 2013. URL: https://www.nhmrc.gov.au/guidelines-publications/n57. Assessed 01 March 2017.

158. National Institute for Health and Clinical Excellence. Advanced breast cancer: diagnosis and treatment. Update 2014. URL: http://nice.org.uk/guidance/cg81. Assessed 03 April 2017.

159. National Institute for Health and Clinical Excellence. Antimicrobial stewardship: changing risk-Antimicrobial stewardship: changing risk-related beharelated behaviours in the generviours in the general population. 2017. URL: www.nice.org.uk/guidance/ng63. Assessed 26 March 2017.

160. National Institute for Health and Clinical Excellence. Behaviour change: individual approaches. 2014. URL: http://nice.org.uk/guidance/ph49. 20 March 2017.

161. National Institute for Health and Clinical Excellence. Care of dying adults in the last days of life. 2015. URL: http://nice.org.uk/guidance/ng31. Assessed 16 March 2017.

162. National Institute for Health and Clinical Excellence. Colorectal cancer: diagnosis and management. Update 2014. URL: http://nice.org.uk/guidance/cg131. Assessed 23 March 2017.

163. National Institute for Health and Clinical Excellence. Diabetes (type 1 and type 2) in children and young people: diagnosis and management. Update 2016. URL: www.nice.org.uk/guidance/ng18. Assessed 21 March 2017.

164. National Institute for Health and Clinical Excellence. Diagnosing prostate cancer: PROGENSA PCA3 assay and Prostate Health Index. 2015. URL: www.nice.org.uk/guidance/dg17. Assessed 23 March 2017.

165. National Institute for Health and Clinical Excellence. Early and locally advanced breast cancer: diagnosis and treatment. Update 2017. URL: http://nice.org.uk/guidance/cg80. Assessed 03 April 2017.

166. National Institute for Health and Clinical Excellence. Familial breast cancer: classification and care of people at risk of familial breast cancer and management of breast cancer and related risks in people with a family history of breast cancer. Update 2017. URL: www.nice.org.uk/guidance/cg164. Assessed 24 March 2017.

167. National Institute for Health and Clinical Excellence. Gene expression profiling and expanded immunohistochemistry tests for guiding adjuvant chemotherapy decisions in early breast cancer management: MammaPrint, Oncotype DX, IHC4 and Mammostrat. 2013. URL: www.nice.org.uk/guidance/dg10. Assessed 24 March 2017.

168. National Institute for Health and Clinical Excellence. Low back pain and sciatica in over 16s: assessment and management. 2016. URL: http://nice.org.uk/guidance/ng59. Assessed 01 March 2017.

169. National Institute for Health and Clinical Excellence. Melanoma: assessment and management. 2015. URL: http://nice.org.uk/guidance/ng14. Assessed 31 March 2017.

170. National Institute for Health and Clinical Excellence. Neuropathic pain in adults: pharmacological management in nonspecialist settings. 2017. URL: www.nice.org.uk/guidance/cg173. Assessed 02 May 2017.

171. National Institute for Health and Clinical Excellence. Obesity prevention. Update 2015. URL: http://nice.org.uk/guidance/cg43. Assessed 01 March 2017.

172. National Institute for Health and Clinical Excellence. Obesity: working with local communities. 2012. URL: http://nice.org.uk/guidance/ph42. Assessed 01 March 2017.

173. National Institute for Health and Clinical Excellence. Pneumonia in adults: diagnosis and management. 2014 URL: http://nice.org.uk/guidance/cg191. Assessed 10 Feb 2017.

174. National Institute for Health and Clinical Excellence. Preventing excess weight gain. 2015. URL: http://nice.org.uk/guidance/ng7. Assessed 14 March 2017.

175. National Institute for Health and Clinical Excellence. Prostate cancer: diagnosis and treatment. 2014. URL: www.nice.org.uk/guidance/cg175. Assessed 23 March 2017.

176. National Institute for Health and Clinical Excellence. Venous thromboembolism: reducing the risk for patients in hospital. Update 2015. URL: http://nice.org.uk/guidance/cg92. Assessed 03 March 2017.

177. National Institute for Health and Clinical Excellence. Weight management: lifestyle services for overweight or obese children and young people. 2013. URL: http://nice.org.uk/guidance/ph47. Assessed 01 March 2017.

178. Wright AA, Bohlke K, Armstrong DK, Bookman MA, Cliby WA, Coleman RL, Dizon DS, Kash JJ, Meyer LA, Moore KN *et al*: Neoadjuvant Chemotherapy for Newly Diagnosed, Advanced Ovarian Cancer: Society of Gynecologic Oncology and American Society of Clinical Oncology Clinical Practice Guideline. *Journal of clinical oncology : official journal of the American Society of Clinical Oncology* 2016, 34(28):3460-3473.

179. Qaseem A, Wilt TJ, McLean RM, Forciea MA: Noninvasive Treatments for Acute, Subacute, and Chronic Low Back Pain: A Clinical Practice Guideline From the American College of Physicians. *Annals of internal medicine* 2017, 166(7):514-530.

180. Peeters M, Leroy R, Robays J, Veereman G, Bielen D, Ceelen W, Danse E, De Man M, Demetter P, Flamen P, Hendlisz A, Sinapi I, Vanbeckevoort D, Van Cutsem E, Ysebaert D, van Gils P, Veerbeek L, Smit Y, Verleye L. Colon Cancer: Diagnosis, Treatment and Follow-up –Summary. Good Clinical Practice (GCP) Brussels: Belgian Health Care Knowledge Centre (KCE). KCE Reports 218Cs. D/2014/10.273/14. 2014. URL: https://kce.fgov.be/sites/default/files/atoms/files/KCE_218_Colon_cancer.pdf. Assessed 26 March 2017.

181. Douketis JD, Spyropoulos AC, Spencer FA, Mayr M, Jaffer AK, Eckman MH, Dunn AS, Kunz R: Perioperative management of antithrombotic therapy: Antithrombotic Therapy and Prevention of Thrombosis, 9th ed: American College of Chest Physicians Evidence-Based Clinical Practice Guidelines. *Chest* 2012, 141(2 Suppl):e326S-e350S.

182. Apovian CM, Aronne LJ, Bessesen DH, McDonnell ME, Murad MH, Pagotto U, Ryan DH, Still CD: Pharmacological management of obesity: an endocrine Society clinical practice guideline. *The Journal of clinical endocrinology and metabolism* 2015, 100(2):342-362.

183. Recht A, Comen EA, Fine RE, Fleming GF, Hardenbergh PH, Ho AY, Hudis CA, Hwang ES, Kirshner JJ, Morrow M *et al*: Postmastectomy Radiotherapy: An American Society of Clinical Oncology, American Society for Radiation Oncology, and Society of Surgical Oncology Focused Guideline Update. *Practical radiation oncology* 2016, 6(6):e219-e234.

184. Khorana AA, Mangu PB, Berlin J, Engebretson A, Hong TS, Maitra A, Mohile SG, Mumber M, Schulick R, Shapiro M *et al*: Potentially Curable Pancreatic Cancer: American Society of Clinical Oncology Clinical Practice Guideline. *Journal of clinical oncology : official journal of the American Society of Clinical Oncology* 2016, 34(21):2541-2556.

185. Practice bulletin no. 136: management of abnormal uterine bleeding associated with ovulatory dysfunction. *Obstetrics and gynecology* 2013, 122(1):176-185.

186. Practice guidelines for acute pain management in the perioperative setting: an updated report by the American Society of Anesthesiologists Task Force on Acute Pain Management. *Anesthesiology* 2012, 116(2):248-273.

187. Apfelbaum JL, Silverstein JH, Chung FF, Connis RT, Fillmore RB, Hunt SE, Nickinovich DG, Schreiner MS, Silverstein JH, Apfelbaum JL *et al*: Practice guidelines for postanesthetic care: an updated report by the American Society of Anesthesiologists Task Force on Postanesthetic Care. *Anesthesiology* 2013, 118(2):291-307.

188. Chang GJ, Kaiser AM, Mills S, Rafferty JF, Buie WD: Practice parameters for the management of colon cancer. *Diseases of the colon and rectum* 2012, 55(8):831-843.

189. Gould MK, Garcia DA, Wren SM, Karanicolas PJ, Arcelus JI, Heit JA, Samama CM: Prevention of VTE in nonorthopedic surgical patients: Antithrombotic Therapy and Prevention of Thrombosis, 9th ed: American College of Chest Physicians Evidence-Based Clinical Practice Guidelines. *Chest* 2012, 141(2 Suppl):e227S-e277S.

190. Kahn SR, Lim W, Dunn AS, Cushman M, Dentali F, Akl EA, Cook DJ, Balekian AA, Klein RC, Le H *et al*: Prevention of VTE in nonsurgical patients: Antithrombotic Therapy and Prevention of Thrombosis, 9th ed: American College of Chest Physicians Evidence-Based Clinical Practice Guidelines. *Chest* 2012, 141(2 Suppl):e195S-e226S.

191. Falck-Ytter Y, Francis CW, Johanson NA, Curley C, Dahl OE, Schulman S, Ortel TL, Pauker SG, Colwell CW, Jr.: Prevention of VTE in orthopedic surgery patients: Antithrombotic Therapy and Prevention of Thrombosis, 9th ed: American College of Chest Physicians Evidence-Based Clinical Practice Guidelines. *Chest* 2012, 141(2 Suppl):e278S-e325S.

192. de Sanjose S, Temin S, Garland S, Eckert LO, Arrossi S: Primary Prevention of Cervical Cancer: American Society of Clinical Oncology Resource-Stratified Guideline Summary. *Journal of oncology practice* 2017, 13(7):452-457.

193. Prostate Cancer Foundation of Australia and Cancer Council Australia. Clinical Practice Guidelines for PSA Testing and Early Management of Test-Detected Prostate Cancer. 2016. URL: http://www.prostate.org.au/media/611493/PSA-Testing-Guidelines-Short-Form.pdf. Assessed 26 March 2017. In*.*

194. Resnick MJ, Lacchetti C, Bergman J, Hauke RJ, Hoffman KE, Kungel TM, Morgans AK, Penson DF: Prostate cancer survivorship care guideline: American Society of Clinical Oncology Clinical Practice Guideline endorsement. *Journal of clinical oncology : official journal of the American Society of Clinical Oncology* 2015, 33(9):1078-1085.

195. Brown DB, Nikolic B, Covey AM, Nutting CW, Saad WE, Salem R, Sofocleous CT, Sze DY: Quality improvement guidelines for transhepatic arterial chemoembolization, embolization, and chemotherapeutic infusion for hepatic malignancy. *Journal of vascular and interventional radiology : JVIR* 2012, 23(3):287-294.

196. Parkin P, Connor Gorber S, Shaw E, Bell N, Jaramillo A, Tonelli M, Brauer P: Recommendations for growth monitoring, and prevention and management of overweight and obesity in children and youth in primary care. *CMAJ : Canadian Medical Association journal = journal de l'Association medicale canadienne* 2015, 187(6):411-421.

197. Wolff AC, Hammond ME, Hicks DG, Dowsett M, McShane LM, Allison KH, Allred DC, Bartlett JM, Bilous M, Fitzgibbons P *et al*: Recommendations for human epidermal growth factor receptor 2 testing in breast cancer: American Society of Clinical Oncology/College of American Pathologists clinical practice guideline update. *Journal of clinical oncology : official journal of the American Society of Clinical Oncology* 2013, 31(31):3997-4013.

198. Robays J, Stordeur S, Hulstaert F, Baurain J-F, Brochez L, Caplanusi T, Claes K, Legius E, Rottey S, Schrijvers D, t'Kint de Roodenbeke D, Ullman U, Van Maerken T, Poppe B. Oncogenetic testing, diagnosis and follow-up in Birt-Hogg-Dubé syndrome, familial atypical multiple mole melanoma syndrome and neurofibromatosis 1 and 2. Good Clinical Practice (GCP) Brussels: Belgian Health Care Knowledge Centre (KCE). KCE Reports 243. D/2015/10.273/34 2015. URL: https://kce.fgov.be/sites/default/files/atoms/files/KCE_243_oncogenetic_testing_Neurofibromatosis_Report.pdf. Assessed 21 Macrh 2017.

199. Robays J, Stordeur S, Hulstaert F, van Maerken T, Claes K, Janin N, Matthijs G, ‘t Kint de Roodenbeke D, Berlière M, Wildiers H, Poppe B. Oncogenetic testing and follow-up for women with familial breast/ovarian cancer, Li-Fraumeni syndrome and Cowden syndrome. Good Clinical Practice (GCP) Brussels: Belgian Health Care Knowledge Centre (KCE). KCE Reports 236. D/2015/10.273/09. 2015. URL: https://kce.fgov.be/sites/default/files/atoms/files/KCE_236_oncogenetic%20testing_Report_0.pdf. Assessed 26 March 2017.

200. Rodrigues G, Yao X, Loblaw A, Brundage M, Chin J, Genitourinary Cancer Disease Site Group. Low-dose rate brachytherapy for patients with low-or intermediate-risk prostate cancer. Toronto, ON: Cancer Care Ontario; Program in Evidence-based Care Evidence-Based Series No.: 3-10 Version 2. 2012. URL: https://www.cancercare.on.ca/common/pages/UserFile.aspx?fileId=254196. Assessed 23 March 2017.

201. Evans JA, Early DS, Fukami N, Ben-Menachem T, Chandrasekhara V, Chathadi KV, Decker GA, Fanelli RD, Fisher DA, Foley KQ *et al*: The role of endoscopy in Barrett's esophagus and other premalignant conditions of the esophagus. *Gastrointestinal endoscopy* 2012, 76(6):1087-1094.

202. Evans JA, Early DS, Chandraskhara V, Chathadi KV, Fanelli RD, Fisher DA, Foley KQ, Hwang JH, Jue TL, Pasha SF *et al*: The role of endoscopy in the assessment and treatment of esophageal cancer. *Gastrointestinal endoscopy* 2013, 77(3):328-334.

203. Fisher DA, Shergill AK, Early DS, Acosta RD, Chandrasekhara V, Chathadi KV, Decker GA, Evans JA, Fanelli RD, Foley KQ *et al*: Role of endoscopy in the staging and management of colorectal cancer. *Gastrointestinal endoscopy* 2013, 78(1):8-12.

204. Henry NL, Somerfield MR, Abramson VG, Allison KH, Anders CK, Chingos DT, Hurria A, Openshaw TH, Krop IE: Role of Patient and Disease Factors in Adjuvant Systemic Therapy Decision Making for Early-Stage, Operable Breast Cancer: American Society of Clinical Oncology Endorsement of Cancer Care Ontario Guideline Recommendations. *Journal of clinical oncology : official journal of the American Society of Clinical Oncology* 2016, 34(19):2303-2311.

205. Royal College of Obstetricians and Gynaecologists. The Initial Management of Chronic Pelvic Pain. Green-top Guideline No. 41. 2012. URL: https://www.rcog.org.uk/en/guidelines-research-services/guidelines/gtg41/. Assessed 14 March 2017.

206. Royal Dutch Society for Physical Therapy. KNGF Guideline Low back pain. 2013. URL: https://www.fysionet-evidencebased.nl/index.php/kngf-guidelines-in-english. Assessed 01 March 2017.

207. Zerey M, Hawver LM, Awad Z, Stefanidis D, Richardson W, Fanelli RD: SAGES evidence-based guidelines for the laparoscopic resection of curable colon and rectal cancer. *Surgical endoscopy* 2013, 27(1):1-10.

208. Moyer VA: Screening and behavioral counseling interventions in primary care to reduce alcohol misuse: U.S. preventive services task force recommendation statement. *Annals of internal medicine* 2013, 159(3):210-218.

209. Moyer VA: Screening for and management of obesity in adults: U.S. Preventive Services Task Force recommendation statement. *Annals of internal medicine* 2012, 157(5):373-378.

210. Siu AL: Screening for Breast Cancer: U.S. Preventive Services Task Force Recommendation Statement. *Annals of internal medicine* 2016, 164(4):279-296.

211. Moyer VA: Screening for cervical cancer: U.S. Preventive Services Task Force recommendation statement. *Annals of internal medicine* 2012, 156(12):880-891, w312.

212. Qaseem A, Denberg TD, Hopkins RH, Jr., Humphrey LL, Levine J, Sweet DE, Shekelle P: Screening for colorectal cancer: a guidance statement from the American College of Physicians. *Annals of internal medicine* 2012, 156(5):378-386.

213. Bibbins-Domingo K, Grossman DC, Curry SJ, Davidson KW, Epling JW, Jr., Garcia FAR, Gillman MW, Harper DM, Kemper AR, Krist AH *et al*: Screening for Colorectal Cancer: US Preventive Services Task Force Recommendation Statement. *Jama* 2016, 315(23):2564-2575.

214. Moyer VA: Screening for gestational diabetes mellitus: U.S. Preventive Services Task Force recommendation statement. *Annals of internal medicine* 2014, 160(6):414-420.

215. Moyer VA: Screening for oral cancer: U.S. Preventive Services Task Force recommendation statement. *Annals of internal medicine* 2014, 160(1):55-60.

216. Moyer VA: Screening for ovarian cancer: U.S. Preventive Services Task Force reaffirmation recommendation statement. *Annals of internal medicine* 2012, 157(12):900-904.

217. Basch E, Oliver TK, Vickers A, Thompson I, Kantoff P, Parnes H, Loblaw DA, Roth B, Williams J, Nam RK: Screening for prostate cancer with prostate-specific antigen testing: American Society of Clinical Oncology Provisional Clinical Opinion. *Journal of clinical oncology : official journal of the American Society of Clinical Oncology* 2012, 30(24):3020-3025.

218. Qaseem A, Barry MJ, Denberg TD, Owens DK, Shekelle P: Screening for prostate cancer: a guidance statement from the Clinical Guidelines Committee of the American College of Physicians. *Annals of internal medicine* 2013, 158(10):761-769.

219. Moyer VA: Screening for prostate cancer: U.S. Preventive Services Task Force recommendation statement. *Annals of internal medicine* 2012, 157(2):120-134.

220. Andersen BL, DeRubeis RJ, Berman BS, Gruman J, Champion VL, Massie MJ, Holland JC, Partridge AH, Bak K, Somerfield MR *et al*: Screening, assessment, and care of anxiety and depressive symptoms in adults with cancer: an American Society of Clinical Oncology guideline adaptation. *Journal of clinical oncology : official journal of the American Society of Clinical Oncology* 2014, 32(15):1605-1619.

221. Jeronimo J, Castle PE, Temin S, Shastri SS: Secondary Prevention of Cervical Cancer: American Society of Clinical Oncology Resource-Stratified Clinical Practice Guideline Summary. *Journal of oncology practice* 2017, 13(2):129-133.

222. Denduluri N, Somerfield MR, Eisen A, Holloway JN, Hurria A, King TA, Lyman GH, Partridge AH, Telli ML, Trudeau ME *et al*: Selection of Optimal Adjuvant Chemotherapy Regimens for Human Epidermal Growth Factor Receptor 2 (HER2) -Negative and Adjuvant Targeted Therapy for HER2-Positive Breast Cancers: An American Society of Clinical Oncology Guideline Adaptation of the Cancer Care Ontario Clinical Practice Guideline. *Journal of clinical oncology : official journal of the American Society of Clinical Oncology* 2016, 34(20):2416-2427.

223. Wong SL, Balch CM, Hurley P, Agarwala SS, Akhurst TJ, Cochran A, Cormier JN, Gorman M, Kim TY, McMasters KM *et al*: Sentinel lymph node biopsy for melanoma: American Society of Clinical Oncology and Society of Surgical Oncology joint clinical practice guideline. *Journal of clinical oncology : official journal of the American Society of Clinical Oncology* 2012, 30(23):2912-2918.

224. Lyman GH, Somerfield MR, Giuliano AE: Sentinel Lymph Node Biopsy for Patients With Early-Stage Breast Cancer: 2016 American Society of Clinical Oncology Clinical Practice Guideline Update Summary. *Journal of oncology practice* 2017, 13(3):196-198.

225. Lyman GH, Somerfield MR, Bosserman LD, Perkins CL, Weaver DL, Giuliano AE: Sentinel Lymph Node Biopsy for Patients With Early-Stage Breast Cancer: American Society of Clinical Oncology Clinical Practice Guideline Update. *Journal of clinical oncology : official journal of the American Society of Clinical Oncology* 2016:Jco2016710947.

226. Moran MS, Schnitt SJ, Giuliano AE, Harris JR, Khan SA, Horton J, Klimberg S, Chavez-MacGregor M, Freedman G, Houssami N *et al*: Society of Surgical Oncology-American Society for Radiation Oncology consensus guideline on margins for breast-conserving surgery with whole-breast irradiation in stages I and II invasive breast cancer. *Journal of clinical oncology : official journal of the American Society of Clinical Oncology* 2014, 32(14):1507-1515.

227. Morrow M, Van Zee KJ, Solin LJ, Houssami N, Chavez-MacGregor M, Harris JR, Horton J, Hwang S, Johnson PL, Marinovich ML *et al*: Society of Surgical Oncology-American Society for Radiation Oncology-American Society of Clinical Oncology Consensus Guideline on Margins for Breast-Conserving Surgery With Whole-Breast Irradiation in Ductal Carcinoma in Situ. *Practical radiation oncology* 2016, 6(5):287-295.

228. Varghese TK, Jr., Hofstetter WL, Rizk NP, Low DE, Darling GE, Watson TJ, Mitchell JD, Krasna MJ: The society of thoracic surgeons guidelines on the diagnosis and staging of patients with esophageal cancer. *The Annals of thoracic surgery* 2013, 96(1):346-356.

229. Armstrong MJ, Gronseth G, Anderson DC, Biller J, Cucchiara B, Dafer R, Goldstein LB, Schneck M, Messe SR: Summary of evidence-based guideline: periprocedural management of antithrombotic medications in patients with ischemic cerebrovascular disease: report of the Guideline Development Subcommittee of the American Academy of Neurology. *Neurology* 2013, 80(22):2065-2069.

230. Giordano SH, Temin S, Kirshner JJ, Chandarlapaty S, Crews JR, Davidson NE, Esteva FJ, Gonzalez-Angulo AM, Krop I, Levinson J *et al*: Systemic therapy for patients with advanced human epidermal growth factor receptor 2-positive breast cancer: American Society of Clinical Oncology clinical practice guideline. *Journal of clinical oncology : official journal of the American Society of Clinical Oncology* 2014, 32(19):2078-2099.

231. Basch E, Loblaw DA, Oliver TK, Carducci M, Chen RC, Frame JN, Garrels K, Hotte S, Kattan MW, Raghavan D *et al*: Systemic therapy in men with metastatic castration-resistant prostate cancer:American Society of Clinical Oncology and Cancer Care Ontario clinical practice guideline. *Journal of clinical oncology : official journal of the American Society of Clinical Oncology* 2014, 32(30):3436-3448.

232. Tombal B, Desomer A, Jonckheer P, Veereman G, D’Hont C, Van Velthoven R, Feyaerts A, Schrijvers D, Gil T, Renard L, De Meerleer G, Rorive S, Spinnewijn B, Servaes A, Van Damme N, Van Brabandt H. National practice guideline on the treatment of localised prostate cancer – part 2 –Synthesis. Good Clinical Practice (GCP) Brussels: Belgian Health Care Knowledge Centre (KCE). KCE Reports 226Cs. D/2014/10.273/52. 2014. URL: https://kce.fgov.be/sites/default/files/atoms/files/KCE_226_Prostate%20cancer_part%202_Report_1.pdf. Assessed 31 March 2017.

233. Toward Optimized Practice (TOP) Low Back Pain Working Group. 2015 December. Evidence-informed primary care management of low back pain: Clinical practice guideline. Edmonton, AB: Toward Optimized Practice. URL: http://www.topalbertadoctors.org/cpgs/885801. Assessed 08 Feb 2017.

234. Linkins LA, Dans AL, Moores LK, Bona R, Davidson BL, Schulman S, Crowther M: Treatment and prevention of heparin-induced thrombocytopenia: Antithrombotic Therapy and Prevention of Thrombosis, 9th ed: American College of Chest Physicians Evidence-Based Clinical Practice Guidelines. *Chest* 2012, 141(2 Suppl):e495S-e530S.

235. Manchikanti L, Abdi S, Atluri S, Benyamin RM, Boswell MV, Buenaventura RM, Bryce DA, Burks PA, Caraway DL, Calodney AK *et al*: An update of comprehensive evidence-based guidelines for interventional techniques in chronic spinal pain. Part II: guidance and recommendations. *Pain physician* 2013, 16(2 Suppl):S49-283.

236. Dhesy-Thind S, Fletcher GG, Blanchette PS, Clemons MJ, Dillmon MS, Frank ES, Gandhi S, Gupta R, Mates M, Moy B *et al*: Use of Adjuvant Bisphosphonates and Other Bone-Modifying Agents in Breast Cancer: A Cancer Care Ontario and American Society of Clinical Oncology Clinical Practice Guideline. *Journal of clinical oncology : official journal of the American Society of Clinical Oncology* 2017, 35(18):2062-2081.

237. Harris LN, Ismaila N, McShane LM, Andre F, Collyar DE, Gonzalez-Angulo AM, Hammond EH, Kuderer NM, Liu MC, Mennel RG *et al*: Use of Biomarkers to Guide Decisions on Adjuvant Systemic Therapy for Women With Early-Stage Invasive Breast Cancer: American Society of Clinical Oncology Clinical Practice Guideline. *Journal of clinical oncology : official journal of the American Society of Clinical Oncology* 2016, 34(10):1134-1150.

238. Visvanathan K, Hurley P, Bantug E, Brown P, Col NF, Cuzick J, Davidson NE, Decensi A, Fabian C, Ford L *et al*: Use of pharmacologic interventions for breast cancer risk reduction: American Society of Clinical Oncology clinical practice guideline. *Journal of clinical oncology : official journal of the American Society of Clinical Oncology* 2013, 31(23):2942-2962.

239. Lyman GH, Bohlke K, Khorana AA, Kuderer NM, Lee AY, Arcelus JI, Balaban EP, Clarke JM, Flowers CR, Francis CW *et al*: Venous thromboembolism prophylaxis and treatment in patients with cancer: american society of clinical oncology clinical practice guideline update 2014. *Journal of clinical oncology : official journal of the American Society of Clinical Oncology* 2015, 33(6):654-656.

240. Vergote I, Vlayen J, Heus P, Hoogendam J.P, Damen J.A.A.G, van de Wetering F t, van der Baan F.H, Bourgain C, De Grève J, Debruyne D, Fastrez M, Goffin F, Huizing M, Kerger J, Kridelka F, Stroobants S, Tjalma W, Van Dam P, Van de Caveye V, Villeirs G, Vuylsteke P, Fairon N, Zweemer R.P, Hooft L, Scholten R.J.P.M, Verleye L. Ovarian cancer: diagnosis, treatment and follow-up – Summary. Good Clinical Practice (GCP) Brussels: Belgian Health Care Knowledge Centre (KCE). KCE Reports 268Cs. D/2016/10.273/48. 2016. URL: https://kce.fgov.be/sites/default/files/atoms/files/KCE_268_Ovarian_cancer.pdf. Assessed 26 March 2017*.*

241. Bates SM, Greer IA, Middeldorp S, Veenstra DL, Prabulos AM, Vandvik PO: VTE, thrombophilia, antithrombotic therapy, and pregnancy: Antithrombotic Therapy and Prevention of Thrombosis, 9th ed: American College of Chest Physicians Evidence-Based Clinical Practice Guidelines. *Chest* 2012, 141(2 Suppl):e691S-e736S.

242. Washington State Department of Labor and Industries. Conservative care options for occupational carpal tunnel syndrome. Olympia (WA): Washington State Department of Labor and Industries; 2013 Dec 1. 21 p.*.*

243. Washington State Department of Labor and Industries. Guideline for Prescribing Opioids to Treat Pain in Injured Workers. 2013. URL: http://www.lni.wa.gov/ClaimsIns/Providers/TreatingPatients/ByCondition/Opioids/default.asp?utm_source=shortmarketingurl&utm_medium=url&utm_campaign=Opioids. Assessed 14 March 2017.

244. Wildiers H, Stordeur S, Vlayen J, Scholten R, van de Wetering F, Bourgain C, Carly B, Christiaens M-R, Cocquyt V, Lifrange E, Schobbens J-C, Van Goethem M, Villeirs G, Van Limbergen E, Neven P. Breast cancer in women: diagnosis, treatment and follow-up –Synthesis. Good Clinical Practice (GCP) Brussels: Belgian Health Care Knowledge Centre (KCE). KCE Reports 143Cs – 3rd EDITION. D/2013/10.273/37. 2013. URL: https://kce.fgov.be/sites/default/files/atoms/files/KCE_143_Breast_cancer_0_0.pdf. Assessed 03 April 2017.

245. World Health Organization. Diagnostic Criteria and Classification of Hyperglycaemia First Detected in Pregnancy. 2013. URL: http://www.who.int/diabetes/publications/Hyperglycaemia_In_Pregnancy/en/. Assessed 21 March 2017.

246. World Health Organization. WHO guidelines for screening and treatment of precancerous lesions for cervical cancer prevention. 2013. URL: http://apps.who.int/iris/bitstream/10665/94830/1/9789241548694_eng.pdf. Assessed 22 March 2017.

247. World Health Organization. WHO guidelines for treatment of cervical intraepithelial neoplasia 2-3 and adenocarcinoma in situ: cryotherapy, large loop excision of the transformation zone, and cold knife conization. 2014. URL: http://www.who.int/reproductivehealth/publications/cancers/treatment_CIN_2-3/en/. Assessed 21 March 2017.

**The guideline is not evidence-based (n=4)**

248. IKNL (Integraal Kankercentrum Nederland). Epitheliaal Ovariumcarcinoom. 2012. URL: <https://www.oncoline.nl/epitheliaal-ovariumcarcinoom> . Assessed 12 June 2017.

249. IKNL (Integraal Kankercentrum Nederland). Oesofaguscarcinoom. 2015. URL: <https://www.oncoline.nl/oesofaguscarcinoom>. Assessed 12 June 2017.

250. University of Michigan Health System. Obesity prevention and management. 2016. URL: https://www.med.umich.edu/1info/FHP/practiceguides/obesity/obesity.pdf. Assessed 22 Feb 2017.

251. Washington State Department of Labor and Industries. Work-related carpal tunnel syndrome diagnosis and treatment guideline. 2016. URL: [http://www.lni.wa.gov/ClaimsIns/Files/OMD/MedTreat/CarpalTunnel.pdf. Assessed 14 March 2017](http://www.lni.wa.gov/ClaimsIns/Files/OMD/MedTreat/CarpalTunnel.pdf.%20Assessed%2014%20March%202017).

**The topics of the international and German S3-guidelines are not comparable (n=13)**

252. Lipsky BA, Berendt AR, Cornia PB, Pile JC, Peters EJ, Armstrong DG, Deery HG, Embil JM, Joseph WS, Karchmer AW *et al*: 2012 Infectious Diseases Society of America clinical practice guideline for the diagnosis and treatment of diabetic foot infections. *Clinical infectious diseases : an official publication of the Infectious Diseases Society of America* 2012, 54(12):e132-173.

253. Kottke T, Wilkinson J, Baechler C, Danner C, Erickson K, O’Connor P, Sanford M, Straub R. Institute for Clinical Systems Improvement. Healthy Lifestyles. UpdateJanuary 2016. URL: https://www.icsi.org/guidelines__more/catalog_guidelines_and_more/catalog_guidelines/catalog_prevention__screening_guidelines/healthy_lifestyles/. Assessed 20 March 2017.

254. National Institute for Health and Clinical Excellence. Chronic kidney disease in adults: assessment and management. Update 2015. URL: www.nice.org.uk/guidance/cg182. Assessed 02 May 2017.

255. National Institute for Health and Clinical Excellence. Diabetic foot problems: prevention and management. Update 2016. URL: http://nice.org.uk/guidance/ng19. Assessed 07 July 2017.

256. Dickinson J, Tsakonas E, Conner Gorber S, Lewin G, Shaw E, Singh H, Joffres M, Birtwhistle R, Tonelli M, Mai V *et al*: Recommendations on screening for cervical cancer. *CMAJ : Canadian Medical Association journal = journal de l'Association medicale canadienne* 2013, 185(1):35-45.

257. Registered Nurses’ Association of Ontario. Assessment and Management of Foot Ulcers for People with Diabetes (2nd ed.). Toronto, ON: Registered Nurses’ Association of Ontario. 2013. URL: http://rnao.ca/sites/rnao-ca/files/Assessment_and_Management_of_Foot_Ulcers_for_People_with_Diabetes_Second_Edition1.pdf. Assessed 08 Feb 2017.

258. Registered Nurses’ Association of Ontario. Assessment and Management of Pain (3rd ed.). Toronto, ON: Registered Nurses’ Association of Ontario. 2013. URL: http://rnao.ca/sites/rnao-ca/files/AssessAndManagementOfPain2014.pdf. Assessed 14 March 2017.

259. Registered Nurses’ Association of Ontario. Engaging Clients Who Use Substances. Toronto, ON: Registered Nurses’ Association of Ontario. 2015. URL: http://rnao.ca/sites/rnao-ca/files/Engaging_Clients_Who_Use_Substances_13_WEB.pdf. Assessed 20 Macrh 2017.

260. Scottish Intercollegiate Guidelines Network (SIGN). Antithrombotics: indications and management. Edinburgh: SIGN (SIGN publication no. 129). 2012, update 2013. URL: http://www.sign.ac.uk. Assessed 13 June 2017.

261. Scottish Intercollegiate Guidelines Network (SIGN). Management of perinatal mood disorders. Edinburgh: SIGN (SIGN publication no. 127). 2012. URL: http://www.sign.ac.uk. Assessed 02 May 2017.

262. University of Michigan Health System. Venous Thromboembolism (VTE). 2014. URL: https://www.med.umich.edu/1info/FHP/practiceguides/vte/vte.pdf. Assessed 17 March 2017.

263. Working Group of the Clinical Practice Guideline for the Prevention and Treatment of Suicidal Behaviour. Clinical Practice Guideline for the Prevention and Treatment of Suicidal Behaviour. Ministry of Health, Social Policy and Equality National Health System Quality Plan. Galician Agency for Health Technology Assessment (avalia-t), 2012, SNS Clinical Practice Guidelines: Avalia-t 2010/02. URL: http://www.guiasalud.es/GPC/GPC_481_Conducta_Suicida_Avaliat_compl_en.pdf. Assessed 14 March 2017.

264. World Health Organization. Guidelines for the identification and management of substance use and substance use disorders in pregnancy. 2014. URL: http://apps.who.int/iris/bitstream/10665/107130/1/9789241548731_eng.pdf. Assessed 20 March 2017
