# Supplementary material for: Guideline-based quality indicators—a systematic comparison of German and international clinical practice guidelines
Source: Implement Sci. 2019 Jul 9;14:71. doi: 10.1186/s13012-019-0918-y (PMC6617919; doi:10.1186/s13012-019-0918-y)
Supplement: Supplementary file 4 — Quality appraisal of included CPGs. (DOCX 35 kb) [file 13012_2019_918_MOESM4_ESM.docx]

**Additional file 4**:

**Quality appraisal of included CPGs** - items and standardized domain scores of the domain “Methodological Rigor of Guideline Development” (German Instrument for Methodological Guideline Appraisal, DELBI)

Table a: Quality appraisal of German S3-CPGs

| German S3-CPG |  | Item 1 (systematic methods were used to search for evidence)* | Item 2 (the criteria for selecting the evidence are clearly described)* | Item 3 (the methods used for formulating the recommendations are clearly described)* | Item 4 (health benefits, side effects and risks have been considered in formulating the recommendations* | Item 5 (there is an explicit link between the recommendations and the supporting evidence)* | Item 6 (the guideline has been externally reviewed by experts prior to its publication)* | Item 7 (a procedure for updating the guideline is provided)* | Standardized domain score (%) |
| --- | --- | --- | --- | --- | --- | --- | --- | --- | --- |
| 021/007OL 2013 | Reviewer 1 | 4 | 4 | 3 | 2 | 4 | 2 | 3 | 71 |
|  | Reviewer 2 | 4 | 4 | 4 | 2 | 4 | 1 | 3 |  |
| 021/023OL 2014 | Reviewer 1 | 4 | 3 | 3 | 3 | 4 | 1 | 2 | 64 |
|  | Reviewer 2 | 4 | 4 | 3 | 2 | 3 | 2 | 3 |  |
| 032/009OL 2012 | Reviewer 1 | 4 | 3 | 4 | 3 | 4 | 1 | 3 | 67 |
|  | Reviewer 2 | 4 | 2 | 4 | 2 | 4 | 1 | 3 |  |
| 032/024OL 2016 | Reviewer 1 | 4 | 4 | 3 | 2 | 4 | 3 | 3 | 74 |
|  | Reviewer 2 | 4 | 4 | 3 | 2 | 4 | 2 | 3 |  |
| 032/035OL 2013 | Reviewer 1 | 4 | 4 | 3 | 2 | 4 | 1 | 2 | 64 |
|  | Reviewer 2 | 4 | 3 | 3 | 2 | 4 | 2 | 3 |  |
| 032/045OL 2012 | Reviewer 1 | 4 | 4 | 4 | 3 | 4 | 1 | 2 | 74 |
|  | Reviewer 2 | 4 | 4 | 4 | 3 | 4 | 1 | 3 |  |
| 043/022OL 2014 | Reviewer 1 | 4 | 4 | 4 | 3 | 4 | 3 | 3 | 83 |
|  | Reviewer 2 | 4 | 4 | 4 | 3 | 4 | 2 | 3 |  |
| 128/001OL 2015 | Reviewer 1 | 4 | 4 | 3 | 2 | 4 | 2 | 2 | 67 |
|  | Reviewer 2 | 4 | 3 | 3 | 2 | 4 | 2 | 3 |  |
| nvl/001d 2015 | Reviewer 1 | 4 | 3 | 3 | 2 | 2 | 2 | 3 | 64 |
|  | Reviewer 2 | 4 | 2 | 3 | 3 | 3 | 3 | 4 |  |
| nvl/001f 2012 | Reviewer 1 | 4 | 2 | 3 | 1 | 2 | 2 | 3 | 57 |
|  | Reviewer 2 | 4 | 3 | 3 | 1 | 3 | 3 | 4 |  |
| nvl/007 2015 | Reviewer 1 | 4 | 3 | 4 | 2 | 2 | 2 | 3 | 76 |
|  | Reviewer 2 | 4 | 4 | 4 | 3 | 3 | 4 | 4 |  |
| 001/018 2013 | Reviewer 1 | 4 | 3 | 3 | 2 | 3 | 1 | 3 | 62 |
|  | Reviewer 2 | 4 | 3 | 3 | 3 | 4 | 1 | 3 |  |
| 003/001 2015 | Reviewer 1 | 4 | 4 | 3 | 2 | 3 | 2 | 3 | 69 |
|  | Reviewer 2 | 4 | 4 | 3 | 3 | 3 | 2 | 3 |  |
| 015/070 2014 | Reviewer 1 | 4 | 3 | 3 | 2 | 4 | 2 | 2 | 67 |
|  | Reviewer 2 | 4 | 3 | 3 | 3 | 4 | 3 | 2 |  |
| 038/019 2012 | Reviewer 1 | 4 | 4 | 4 | 3 | 4 | 2 | 3 | 79 |
|  | Reviewer 2 | 4 | 4 | 3 | 3 | 3 | 2 | 3 |  |
| 050/001 2014 | Reviewer 1 | 4 | 3 | 3 | 1 | 4 | 2 | 3 | 74 |
|  | Reviewer 2 | 4 | 4 | 4 | 3 | 4 | 3 | 3 |  |
| 057/023 2014 | Reviewer 1 | 4 | 3 | 3 | 2 | 2 | 2 | 2 | 50 |
|  | Reviewer 2 | 4 | 2 | 3 | 2 | 2 | 2 | 2 |  |
| 145/003 2014 | Reviewer 1 | 4 | 3 | 3 | 2 | 4 | 3 | 3 | 76 |
|  | Reviewer 2 | 4 | 3 | 3 | 3 | 4 | 4 | 3 |  |

* one = “strongly disagree”, two = “disagree”, three = “agree” and four = “strongly agree”

Table b: Quality appraisal of international CPGs

| International CPG |  | Item 1 (systematic methods were used to search for evidence)* | Item 2 (the criteria for selecting the evidence are clearly described)* | Item 3 (the methods used for formulating the recommendations are clearly described)* | Item 4 (health benefits, side effects and risks have been considered in formulating the recommendations)* | Item 5 (there is an explicit link between the recommendations and the supporting evidence)* | Item 6 (the guideline has been externally reviewed by experts prior to its publication)* | Item 7 (a procedure for updating the guideline is provided)* | Standardized domain score (%) |
| --- | --- | --- | --- | --- | --- | --- | --- | --- | --- |
| CCHMC VTE 2014 | Reviewer 1 | 3 | 3 | 2 | 3 | 4 | 2 | 2 | 55 |
|  | Reviewer 2 | 3 | 4 | 3 | 2 | 4 | 1 | 1 |  |
| CTFPHC prostate 2014 | Reviewer 1 | 3 | 3 | 3 | 3 | 4 | 2 | 2 | 57 |
|  | Reviewer 2 | 3 | 3 | 3 | 3 | 3 | 2 | 1 |  |
| CTFPHC obesity 2015 | Reviewer 1 | 3 | 3 | 3 | 3 | 4 | 2 | 2 | 57 |
|  | Reviewer 2 | 3 | 3 | 3 | 3 | 3 | 2 | 1 |  |
| CTFPHC colorectal 2016 | Reviewer 1 | 3 | 3 | 3 | 3 | 4 | 2 | 2 | 55 |
|  | Reviewer 2 | 3 | 2 | 3 | 3 | 3 | 2 | 1 |  |
| ICSI backpain 2012 | Reviewer 1 | 2 | 2 | 2 | 2 | 4 | 2 | 3 | 48 |
|  | Reviewer 2 | 3 | 1 | 2 | 2 | 4 | 2 | 3 |  |
| ICSI palliative 2013 | Reviewer 1 | 2 | 2 | 2 | 2 | 4 | 2 | 3 | 48 |
|  | Reviewer 2 | 3 | 1 | 2 | 2 | 4 | 2 | 3 |  |
| ICSI obesity 2013 | Reviewer 1 | 2 | 2 | 2 | 2 | 4 | 2 | 3 | 48 |
|  | Reviewer 2 | 3 | 1 | 2 | 2 | 4 | 2 | 3 |  |
| ICSI hypo 2014 | Reviewer 1 | 2 | 2 | 2 | 2 | 4 | 2 | 3 | 48 |
|  | Reviewer 2 | 3 | 1 | 2 | 2 | 4 | 2 | 3 |  |
| ICSI diabtypeII 2014 | Reviewer 1 | 3 | 2 | 2 | 3 | 4 | 2 | 3 | 52 |
|  | Reviewer 2 | 3 | 2 | 2 | 2 | 4 | 1 | 3 |  |
| ICSI pain 2016 | Reviewer 1 | 3 | 3 | 2 | 3 | 3 | 3 | 3 | 52 |
|  | Reviewer 2 | 3 | 1 | 2 | 3 | 3 | 2 | 2 |  |
| KCE gastrointest 2012 | Reviewer 1 | 4 | 2 | 2 | 2 | 3 | 3 | 1 | 52 |
|  | Reviewer 2 | 4 | 3 | 2 | 2 | 4 | 3 | 1 |  |
| NICE obesity 2014 | Reviewer 1 | 4 | 4 | 2 | 4 | 3 | 4 | 4 | 83 |
|  | Reviewer 2 | 3 | 4 | 2 | 4 | 3 | 4 | 4 |  |
| NICE weight 2014 | Reviewer 1 | 4 | 4 | 2 | 3 | 4 | 4 | 4 | 81 |
|  | Reviewer 2 | 3 | 4 | 2 | 3 | 3 | 4 | 4 |  |
| NICE diabpreg 2015 | Reviewer 1 | 4 | 4 | 3 | 3 | 3 | 4 | 4 | 83 |
|  | Reviewer 2 | 4 | 4 | 2 | 3 | 3 | 4 | 4 |  |
| NICE diabtypeI 2015 | Reviewer 1 | 4 | 4 | 3 | 4 | 2 | 4 | 4 | 83 |
|  | Reviewer 2 | 4 | 4 | 3 | 3 | 2 | 4 | 4 |  |
| NICE diabtypeII 2016 | Reviewer 1 | 4 | 4 | 2 | 4 | 3 | 4 | 4 | 81 |
|  | Reviewer 2 | 3 | 4 | 2 | 3 | 3 | 4 | 4 |  |
| NICE menstrualbleeding 2016 | Reviewer 1 | 4 | 4 | 2 | 3 | 3 | 4 | 4 | 81 |
|  | Reviewer 2 | 3 | 4 | 2 | 4 | 3 | 4 | 4 |  |
| NICE bipolar 2016 | Reviewer 1 | 3 | 4 | 2 | 4 | 2 | 4 | 4 | 74 |
|  | Reviewer 2 | 3 | 4 | 2 | 3 | 2 | 4 | 4 |  |
| SIGN ovar 2013 | Reviewer 1 | 4 | 2 | 2 | 2 | 3 | 3 | 4 | 60 |
|  | Reviewer 2 | 3 | 2 | 2 | 2 | 3 | 3 | 4 |  |
| SIGN breast 2013 | Reviewer 1 | 3 | 2 | 2 | 2 | 3 | 3 | 4 | 57 |
|  | Reviewer 2 | 3 | 2 | 2 | 2 | 3 | 3 | 4 |  |
| SIGN pain 2013 | Reviewer 1 | 4 | 2 | 2 | 2 | 3 | 3 | 4 | 60 |
|  | Reviewer 2 | 3 | 2 | 2 | 2 | 3 | 3 | 4 |  |
| SIGN VTEPrev 2014 | Reviewer 1 | 4 | 2 | 2 | 2 | 3 | 3 | 4 | 60 |
|  | Reviewer 2 | 3 | 2 | 2 | 2 | 3 | 3 | 4 |  |
| SIGN colorectal 2016 | Reviewer 1 | 3 | 2 | 2 | 2 | 3 | 3 | 4 | 57 |
|  | Reviewer 2 | 3 | 2 | 2 | 2 | 3 | 3 | 4 |  |
| SIGN melanoma 2017 | Reviewer 1 | 3 | 2 | 2 | 2 | 3 | 3 | 4 | 57 |
|  | Reviewer 2 | 3 | 2 | 2 | 2 | 3 | 3 | 4 |  |
| SNS diabtypeI 2012 | Reviewer 1 | 3 | 2 | 2 | 2 | 3 | 3 | 2 | 50 |
|  | Reviewer 2 | 3 | 3 | 2 | 3 | 4 | 1 | 2 |  |

* one = “strongly disagree”, two = “disagree”, three = “agree” and four = “strongly agree”
