# Supplementary material for: Guideline-based quality indicators—a systematic comparison of German and international clinical practice guidelines
Source: Implement Sci. 2019 Jul 9;14:71. doi: 10.1186/s13012-019-0918-y (PMC6617919; doi:10.1186/s13012-019-0918-y)
Supplement: Supplementary file 5 — Topic-related QIs in international CPGs and German S3-CPGs and if applicable corresponding recommendations. (DOCX 238 kb) [file 13012_2019_918_MOESM5_ESM.docx]

**Additional file 5: Topic-related QIs in international CPGs and German S3-CPGs and if applicable corresponding recommendations**

| **Acronym**  **international CPG** | **QI_int_[# (S/P/O); reference range rr; title]** | **QI_int_ implicit/explicit derivation from guideline recommendations**  **[yes^i/e^/no]** | **Corresponding recommendation(s) (rec) (GoR, LoE)** | **Acronym**  **corres-ponding German S3-CPG** | **QI_S3_ [# (S/P/O); reference range rr; title]** | **QI_S3_ implicit/explicit derivation from guideline recommendations**  **[yes^i/e^/no]** | **Corresponding recommendation (GoR, LoE)** |
| --- | --- | --- | --- | --- | --- | --- | --- |
| CTFPHC colorectal 2016 | #_int_1 (P); rr: N.R.  the proportion of people aged 50–59 years with whom colorectal cancer screening is discussed | yes^i^ | We recommend screening adults aged 50 to 59 years for colorectal cancer with FOBT (gFOBT or FIT) every two years or flexible sigmoidoscopy every 10 years. (weak rec; moderate-quality evidence) | 021/007OL 2013 | #_S3_1 (P); rr: N.R.  **Angabe Abstand mesorektale Faszie**  Numerator:  Alle Patienten mit Angabe des Abstands zur mesorektalen Faszie im Befundbericht  Denominator:  Alle Patienten mit Rektumkarzinom und MRT oder Dünnschicht-CT des Beckens | yes^e^ | 7.17  Die Befundbeschreibung soll eine Aussage über den Abstand zur mesorektalen Faszie beinhalten (strong rec, consensus-based). |
|  | #_int_2 (P); rr: N.R.  the proportion of people aged 60–74 undergoing FOBT or flexible sigmoidoscopy at recommended intervals | yes^i^ | We recommend screening adults aged 60 to 74 years for colorectal cancer with FOBT (gFOBT or FIT) every two years or flexible sigmoidoscopy every 10 years. (strong rec; moderate-quality evidence) |  | #_S3_2 (P); rr: N.R.  **Untersuchung Lymphknoten**  Numerator:  Anzahl der Patienten mit ≥ 12 pathologisch untersuchten Lymphknoten  Denominator:  Alle Patienten mit KRK, die eine Lymphadenektomie erhalten | yes^e^ | 7.61  12 und mehr Lymphknoten sollen entfernt und untersucht werden (strong rec, consensus-based). |
|  | #_int_3 (O); rr: N.R.  interval cancers diagnosed after a negative screening test | no | n.a. |  | #_S3_3 (P); rr: N.R.  **Qualität TME (totale mesorektale Exzision)**  Numerator:  Anzahl aller Patienten mit guter oder moderater Qualität (Grad 1: Mesorektale Faszie erhalten oder Grad 2: Intramesorektale Einrisse) der TME  Denominator:  Alle Patienten mit radikal operiertem Rektumkarzinom | yes^e^ | 7.66  Da die Qualität eines Operationsresektates unter Berücksichtigung der oben genannten Kategorien Rückschlüsse auf die Prognose bezüglich der Entwicklung eines Lokalrezidives zulässt, soll diese obligat im patho-histologischen Befundbericht wie folgend beschrieben werden:  Die Qualität des Präparates wird beurteilt an der Integrität der mesorektalen Faszie im Falle der Resektion mit 3 Kategorien:  • Grad1 (gut): Mesorektale Faszie erhalten  • Grad 2 (moderat): Intramesorektale Einrisse  • Grad 3 (schlecht): Erreichen der Muscularis propria oder Tumor  Im Falle einer Rektumexstirpation sind bei einer vollständigen Resektion der Levatormuskulatur Präparateinrisse und ein tumorpositiver zirkumferentieller Sicherheitsabstand seltener.  Im patho-histologischen Befundbericht ist deshalb die Beschreibung bezüglich der Radikalität im Bereich der Levatormuskulatur obligat. Hierzu sollen folgende Kategorien Verwendung finden:  • Grad 1 (gut): Levatormuskulatur mitreseziert, keine Eröffnung des Darmes oder des Tumors  • Grad 2 (moderat): Muscularis propria erhalten, keine Eröffnung des Darmes oder des Tumors  • Grad 3 (schlecht): Teile der Muscularis propria fehlen oder Eröffnung des Darmes oder des Tumors  Diese Bewertungen sind vom Pathologen vorzunehmen (strong rec, consensus-based). |
|  | #_int_4 (O); rr: N.R.  harms of follow up testing | no | n.a. |  | #_S3_4 (P); rr: N.R.  **Angabe Resektionsrand**  Numerator:  Anzahl der Patienten, bei denen der Abstand des aboralen Tumorrandes zur aboralen Resektionsgrenze in mm und der Abstand des Tumors zur zirkumferentiellen mesorektalen Resektionsebene in mm dokumentiert wurden.  Denominator:  Alle Patienten mit RK, bei denen der Primärtumor in Form einer TME oder PME reseziert wurde. | yes^e^ | 7.65  Der zirkumferentielle Sicherheitsabstand ist negativ, wenn er 1 mm oder mehr beträgt (R0 „wide“). Ein positiver zirkumferentieller Sicherheitsabstand liegt vor, wenn der zirkumferentielle Sicherheitsabstand weniger als 1 mm beträgt (R0 „close“) oder Tumorgewebe direkt an ihn heranreicht. Der gemessene Abstand soll in Zahlen dokumentiert werden (strong rec, consensus-based). |
|  | #_int_5 (O); rr: N.R.  colorectal cancer incidence and mortality | no | n.a. |  | #_S3_5 (P); rr: N.R.  **Vorstellung Tumorkonferenz**  Numerator:  Anzahl der Patienten, die prätherapeutisch in einer interdisziplinären Tumorkonferenz vorgestellt wurden  Denominator:  Alle Patienten mit Rektumkarzinom und alle Pat. mit Kolonkarzinom Stad. IV | yes^e^ | 7.1  Alle Patienten mit KRK sollen nach Abschluss der Primärtherapie (z.B. Operation, Chemotherapie) in einer interdisziplinären Tumorkonferenz vorgestellt werden.  Bereits prätherapeutisch sollen Patienten in folgenden Konstellationen vorgestellt werden:  -mit Rektumkarzinom  - mit Kolonkarzinom. im Stadium IV  - mit metachronen Fermetastasen  - mit Lokalrezidiven  - vor jeder lokal ablativen Maßnahme RFA/LITT/SIRT. (strong rec, consensus-based) |
|  | #_int_6 (P); rr: N.R.  use of screening colonoscopy and screening with gFOBT or flexible sigmoidoscopy in people aged 75 years and older | yes^i^ | We recommend not screening adults aged 75 years and older for colorectal cancer (weak recommendation; low-quality evidence). |  | #_S3_6 (P); rr: N.R.  **Adjuvante Chemotherapie**  Numerator:  Anzahl der Patienten, die eine adjuvante Chemotherapie erhalten haben.  Denominator:  Alle Patienten mit Kolonkarzinom im UICC-Stadium III, bei denen eine R0-Resektion des Primärtumors erfolgte | yes^e^ | 8.2  Bei Patienten mit einem R0 resezierten Kolonkarzinom im Stadium III ist eine adjuvante Chemotherapie indiziert (GoR: A, LoE: 1a). |
|  |  |  |  |  | #_S3_7 (P); rr: N.R.  **Neoadjuvante Radio- und Radiochemotherapie**  Numerator:  Anzahl der Patienten, die eine neoadjuvante Radio- oder Radiochemotherapie erhalten haben  Denominator:  Alle Patienten mit Rektumkarzinom des mittleren und unteren Drittels und den TNM-Kategorien cT3, 4/cM0 und/oder cN1, 2/cM0, die operiert wurden (= klinisches UICC-Stadium II u. III). | yes^e^ | 8.10  Im UICC-Stadium II und III ist die neoadjuvante Radio- oder Radiochemotherapie indiziert. Eine Sondersituation besteht bei cT1/2-Karzinomen mit fraglichem Lymphknotenbefall; hier ist auch die primäre Operation (mit ggf. adjuvanter Radiochemotherapie bei pN+) eine mögliche Behandlungsoption (GoR: A, LoE: 1b). |
|  |  |  |  |  | #_S3_8 (O); rr: N.R.  **Anastomoseninsuffizienz Rektumkarzinom**  Numerator:  Anzahl der Patienten mit Anastomoseninsuffizienz Grad B (mit Antibiotikagabe o. interventioneller Drainage o.transanaler Lavage/Drainage) oder C ((Re-)-Laparotomie) nach elektiven Eingriffen  Denominator:  Alle Patienten mit Rektumkarzinom, bei denen in einer elektiven primären Tumorresektion eine Anastomose angelegt wurde. | no | n.a. |
|  |  |  |  |  | #_S3_9 (O); rr: N.R.  **Anastomoseninsuffizienz Kolonkarzinom**  Numerator:  Re-Interventionsbedürftige Anastomoseninsuffizienzen Kolon nach elektiven Eingriffen  Denominator:  Alle Patienten mit Kolonkarzinom, bei denen in einer elektiven Tumorresektion eine Anastomose angelegt wurde. | no | n.a. |
|  |  |  |  |  | #_S3_10 (P); rr: N.R.  **Anzeichnung Stomaposition**  Numerator:  Anzahl der Pat. mit präoperativer Anzeichnung der Stomaposition  Denominator:  Alle Pat. mit Rektumkarzinom, bei denen eine Operation mit Stomaanlage durchgeführt wurde | yes^e^ | 7.42  Die Stomaposition soll präoperativ angezeichnet werden (strong rec, consensus-based). |
| SIGN colorectal 2016 | #_int_1 (P); rr: N.R.  number and frequency of surveillance colonoscopies performed on patients with inflammatory bowel disease at low, medium and high risk | yes^i^ | All patients with ulcerative colitis or Crohn’s colitis of 10 years duration should undergo a screening colonoscopy (GoR: D). |  |  |  |  |
|  | #_int_2 (P); rr: N.R.  number of patients who receive follow-up colonoscopy after colonoscopic polypectomy | yes^i^ | Patients who have undergone colonoscopic polypectomy for adenomas should be offered followup colonoscopy based on risk stratification (GoR: D). |  |  |  |  |
|  | #_int_3 (P); rr: N.R.  number and frequency of surveillance colonoscopies performed in patients with ≥5 small adenomas, or ≥3 adenomas with at least one polyp ≥1 cm in size | yes^i^ | Patients with ≥5 small adenomas, or ≥3 adenomas with at least one polyp ≥1 cm in size are at high risk, and should undergo colonoscopy at one year (GoR: D). |  |  |  |  |
|  | #_int_4 (P); rr: N.R.  number of referrals to the Clinical Genetics Service of individuals whose family history is suggestive of a CRC syndrome | yes^i^ | All individuals whose family history is suggestive of a CRC syndrome should be referred to a clinical genetics service for consideration of genetic testing to clarify the risk (GoR: B). |  |  |  |  |
|  | #_int_5 (P); rr: N.R.  the inclusion of tumour differentiation, staging (Dukes’ and TNM systems), margins (peritoneal and CRM) and extramural vascular invasion in pathological reports of colorectal cancer resection specimens | yes^i^ | Pathological reporting of colorectal cancer resection specimens should include information on:   - tumour differentiation - staging (Dukes and TNM systems) - margins (peritoneal and CRM) - extramural vascular invasion (GoR:B). |  |  |  |  |
|  | #_int_6 (O); rr: N.R.  outcomes of treatment, including treatment-related morbidity and mortality, disease-free survival and overall survival | no | n.a. |  |  |  |  |
| KCE gastrointest 2012 | #_int_1 (P); rr: N.R.  **Staging**  Numerator:  All patients diagnosed with oesophageal cancer in a given year discussed at the MDT meeting within 1 month after incidence date.  Denominator:  All patients diagnosed with oesophageal cancer in a given year. | yes^e^ | All patients diagnosed with oesophageal cancer should be discussed at a multidisciplinary meeting (GoR: strong, LoE: low). | 021/023OL 2014 | #_S3_1 (P); rr: N.R.  **Vollständige histopathologische Befundung von Biopsiematerial**  Numerator:  Anzahl Patienten mit Angabe der Art der neoplastischen Läsion (Low Grade Dysplasie/Low Grade Intra Epitheliale Neoplasie, High Grade Dysplasie/High Grade Intraepitheliale Neoplasie=C15x +8077/0, 8077/2., C16x, +8148/0, 8148/2, Tis Klassifikation nach UICC, invasives Karzinom), WHO-hist. Typ , bei invasiven Karzinomen Grading nach aktueller WHO-Klassifikation, Angabe, ob Biopsie aus dem distalen Ösophagus (C 15.5) mit becherzellhaltiger Barrettmukosa  Denominator:  Alle Patienten mit V.a. Neoplasie des Ösophagus (D.00.1, C.15x., C16x) und Biopsie (1.440.9 und 1.440.a) | yes^e^ | 6.19  Der histopathologische Befund am Biopsiematerial soll die folgende Angaben enthalten:  • Art der neoplastischen Läsion (LGD/LGIEN, HGD/HGIEN, Karzinom), insbesondere ob ein invasives Karzinom vorliegt (bei HGD/HGIEN: Klassifikation am Biopsat als Tis nach UICC)  • Histologischer Typ nach WHO (insbesondere Unterscheidung Plattenepithel- versus Adenokarzinom)  • Bei invasiven Adenokarzinomen:  Differenzierungsgrad (Grading) nach aktueller WHO-Klassifikation  • Bei Läsionen im distalen Ösophagus: ist eine Becherzell-haltige Barrett-Mukosa vorhanden? (strong rec, consensus-based) |
|  | #_int_2 (P); rr: N.R.  **Staging**  Numerator:  All patients diagnosed with oesophageal cancer in a given year undergoing a CT neck/thorax/abdomen within 1 month before and after incidence date  Denominator:  All patients diagnosed with oesophageal cancer in a given year | yes^e^ | In patients with newly diagnosed oesophageal cancer, CT scan of the neck (including lower neck region), thorax and abdomen should always be performed (GoR: strong, LoE: low). |  | #_S3_2 (P); rr: N.R.  **Vollständige histopathologische Befundung von lokalen Exzidaten**  Numerator:  Anzahl Patienten mit Angabe der Art der neoplastischen Läsion (C15x +8077/0, 8077/2., C16x, +8148/0, 8148/2), WHOKlass., Grading, Lymphgefäß-und/oder Veneninvasion, Tiefe (Invasionstiefe) + Angabe von zirkulärem und basaler Resektionsrand  Denominator:  Alle Patienten mit Neoplasie des Ösophagus (D.00.1, C.15x, C16x) und endoskopischer Resektion (5.422.2, 5.422.0, 5.422.2,5.422.3) | yes^e^ | 6.21  Der histopathologische Befund an lokalen Exzidaten (endoskopische Resektion; ER) soll folgende Angaben enthalten:  • Größe der neoplastischen Läsion in 3 Dimensionen, wenn möglich  • Art der neoplastischen Läsion  (LGD/LGIEN, HGD/HGIEN, Karzinom) – insbesondere, ob ein invasives Karzinom vorliegt (bei HGD/HGIEN: Klassifikation am Resektat als pTis nach UICC)  • Bei Karzinomnachweis: Histologischer Typ nach WHO (insbesondere Unterscheidung Plattenepithelversus Adenokarzinom, andere seltene Typen)  • Bei invasiven Adenokarzinomen:  Differenzierungsgrad (Grading) nach aktueller WHO-Klassifikation  • Maximale Tiefe der Infiltration: pT1a (m1, m2, m3, m4) / pT1b (sm1, sm2, sm3) plus Infiltrationstiefe in μm (oder höhere pTKategorie)  • Lymphgefäß- und/oder Veneninvasion  (L0 vs. L1, V0 vs. V1)  • Zusammenfassende Einschätzung des LK-Metastasierungsrisikos: Low risk vs. High risk- Resektionsränder bzgl. Der Neoplasie (bei ER in toto zirkulärer und basaler RR; bei „piecemeal“-ER basaler RR, da hier der zirkuläre RR in der Regel histo-pathologisch als RX gewertet werden muss) (strong rec, consensus-based) |
|  | #_int_3 (P); rr: N.R.  **Treatment of mucosal cancer**  *Proportion of patients diagnosed with cT1a oesophageal cancer undergoing endoscopic mucosal resection who had an en bloc resection (non-measurable indicator)* | yes^e^ | Endoscopic mucosal resection (EMR) should be performed whenever possible for a T1a oesophageal cancer, aiming at staging and curative resection. If the staging and R0 resection is pathologically confirmed, this procedure can be considered therapeutic, taking into account other well-defined criteria relating to size, length of Barrett, histological type, differentiation grade and lymphovascular invasion. In case the staging and R0 resection is not confirmed, surgery can be considered (GoR: strong, LoE: low). |  |  |  |  |
|  | #_int_4 (P); rr: N.R.  **Neoadjuvant treatment**  Numerator:  All patients with oesophageal cancer beyond the mucosa (T _2-4_ N_Any_ M_0-1a_) who received neoadjuvant treatment before their surgical intervention.  Denominator:  All patients with oesophageal cancer beyond the mucosa (T _2-4_ N_Any_ M_0-1a_) who underwent a surgical intervention. | yes^e^ | If, after multidisciplinary discussion, neoadjuvant treatment is considered for a locally-advanced oesophageal or junction tumour, neoadjuvant chemoradiotherapy is preferred (GoR: strong, LoE: low). |  | #_S3_3 (P); rr: N.R.  **Vollständige histopathologische Befundung des Operationsresektates**  Numerator:  Anzahl Patienten mit Angabe von Größe der neoplastischen Läsion, Art der Läsion (C15x +8077/0, 8077/2., C16x, +8148/0, 8148/2, Tis), WHO-Klass. Grading, pT, pN, Ratio LK, L, V, R-Status (TNM)  Denominator:  Alle Patienten mit Neoplasie des Ösophagus und chirurgischer Resektion (D.00.1, C.15x, C16x) und chirurgischer Resektion (OPS 5.422.0, alle 5.423, 5.424, 5.425, 5.426) | yes^e^ | 6.22  Der histopathologische Befund an Operationsresektaten soll folgende Angaben enthalten:  • Größe der neoplastischen Läsion  Lokalisation des Tumorzentrums in Beziehung zur ÖGJ und Angabe, ob der Tumor die ÖGJ kreuzt  • Art der neoplastischen Läsion  (LGD/LGIEN, HGD/HGIEN, Karzinom) – insbesondere, ob ein invasives Karzinom vorliegt (bei HGD/HGIEN: Klassifikation als pTis  nach UICC)  • Bei Karzinomnachweis: Histologischer  Typ nach WHO (insbesondere Unterscheidung Plattenepithelversus Adenokarzinom, andere seltene Typen)  Differenzierungsgrad (Grading)  •Maximale Tiefe der Infiltration (pT)  •Lymph- oder Hämangio-Invasion :  L0 vs. L1, V0 vs. V1)  •Resektionsränder (oral, aboral  und zirkumferenziell): R0 / R1  •Lymphknoten-Status nach UICC  (pN) und Ratio aus Zahl der befallenen und untersuchten Lymphknoten (…/…LK) (strong rec, consensus-based) |
|  | #_int_5 (O); rr: N.R.  **Surgery**  *Proportion of surgically treated patients who had a R0 resection (non-measurable indicator)* | yes^e^ | Surgery for oesophageal cancer should be aimed at achieving an R0 resection, and should be considered preferentially through a transthoracic en bloc resection (GoR: strong, LoE: high). |  |  |  |  |
|  | #_int_6 (O); rr: N.R.  **Surgery**  Numerator:  All patients with oesophageal cancer treated with oesophagectomy in a given year dying within 30 days.  Denominator:  All patients with oesophageal cancer treated with oesophagectomy in a given year. | no | n.a. |  | #_S3_4 (P); rr: N.R.  **Therapieempfehlung aus interdisziplinärer Tumorkonferenz**  Numerator:  Anzahl Patienten mit Therapieempfehlung aus interdisziplinärer Tumorkonferenz vor Therapie (Staging abgeschlossen)  Denominator:  Alle Patienten mit Neoplasie des Ösophagus (D.00.1, C.15x, C16x) | yes^e^ | 8.1  Therapieempfehlungen sollen in einer interdisziplinären Tumorkonferenz getroffen werden. Als Grundlage für die Therapieempfehlung sollen Staging-Informationen, die Patienten-Komorbiditäten, der Ernährungsstatus und die Patientenpräferenz berücksichtigt werden (strong rec., consensus-based). |
|  | #_int_7 (P); rr: N.R.  **Surgery**  *Proportion of patients with oesophageal cancer or cancer of the gastro-oesophageal junction who were treated by a radical transthoracic oesophagectomy and two-field lymphadenectomy of abdominal and thoracic lymph nodes (non-measurable indicator)* | yes^e^ | Extensive two-field lymphadenectomy should be standard during oesophagectomy to improve staging, local disease control and potentially cure rate (GoR: strong, LoE: low). |  | #_S3_5 (O); rr: N.R.  **Vollständige endoskopische Resektion einer intraepithelialen Neoplasie oder eines mukosalen Frühkarzinoms im Barrett-Ösophagus**  Numerator:  Anzahl Patienten mit R0  Denominator:  Alle Patienten mit Diagnose einer hochgradigen intraepithelialen Neoplasie (C16x, 8148/2) oder eines mukosalen Karzinoms (=8140/3) L0, V0, G1/G2, keine Ulzerationen, Infiltrationstiefe ≤ m3 im BarrettÖsophagus (K22.7) und endoskopischer Resektion (5.422.2, 5.422.20. 5.422.3, 5.422.4) | yes^e^ | 8.2  a. Bei Nachweis einer hochgradigen intraepithelialen Neoplasie oder eines mukosalen Karzinoms (L0, V0, keine Ulzerationen, Grading G1/G2) im BarrettÖsophagus soll eine endoskopische Resektion durchgeführt werden, da hierdurch neben der Therapie auch ein Staging der Läsion mit der Frage der Tiefeninfiltration erfolgt. (strong rec., consensus-based)  b. Daher ist eine endoskopisch komplette Resektion mit kurativer Intention anzustreben. (strong rec, consensus-based)  d. Nach erfolgreicher Resektion von Neoplasien im Barrett-Ösophagus soll die nicht neoplastische Barrett-Mucosa thermisch abladiert werden, um die Rate an metachronen Neoplasien zu senken. (strong rec, consensus-based) |
|  | #_int_8 (O); rr: N.R.  **Surgery**  *Mean number of resected/evaluated lymph nodes during oesophagectomy (non-measurable indicator)* | yes^e^ |  |  |  |  |  |
|  | #_int_9 (O); rr: N.R.  **Surgery**  *Proportion of patients experiencing anastomotic leakage after oesophagectomy (non-measurable indicator)* | no | n.a. |  | #_S3_6 (O); rr: N.R.  **Vollständige chirurgische Resektion**  Numerator:  Anzahl Patienten mit R0  Denominator:  Alle Patienten mit Neoplasie des Ösophagus (D.00.1, C.15x, C16x) und Operation (chirurgische Resektion OPS 5.422.0, alle 5.423, 5.424, 5.425, 5.426) | yes^e^ | 8.9  Das Ziel der chirurgischen Resektion beim Plattenepithelkarzinom und Adenokarzinom ist die vollständige Entfernung  des Tumors (oral, aboral und in der Zirkumferenz) und der regionären Lymphknoten. (strong re., consensus-based) |
|  | #_int_10 (P); rr: N.R.  **Primary chemoradiotherapy**  Numerator:  All patients with any stage of oesophageal cancer who were treated primary chemoradiotherapy (without surgical resection).  Denominator:  All patients with any stage of oesophageal cancer. | yes^e^ | Definitive concomitant chemoradiotherapy should be considered in patients with locally advanced oesophageal cancer of any histological type (GoR: strong, LoE: moderate):  o If the tumour is considered unresectable;  o If the patient is unfit for surgery;  o If the patient declines surgery.  Definitive concomitant chemoradiotherapy can be considered for patients with cervical oesophageal cancer in order to preserve the larynx (GoR: weak, LoE: low). |  | #_S3_7 (P); rr: N.R.  **präoperative Radiotherapie bei Patienten mit einem Plattenepithelkarzinom des Ösophagus T3/T4**  Numerator:  Anzahl Patienten mit präoperativer Radiochemotherapie  Denominator:  Alle Patienten mit Plattenepithelkarzinom des Ösophagus (C15x) und cT3/cT4 | yes^e^ | 8.27  Bei operablen Patienten mit einem Plattenepithelkarzinom des Ösophagus der Kategorie cT3 und bei resektablen cT4 Tumoren soll eine präoperative Radiochemotherapie mit anschließender kompletter Resektion durchgeführt werden (GoR: A, LoE: 1a) |
|  | #_int_11 (P); rr: N.R.  **Metastatic disease**  Numerator:  All patients diagnosed with metastatic oesophageal cancer that died in a given year and had palliative support within 3 months before death.  Denominator:  All patients diagnosed with metastatic oesophageal cancer that died in a given year. | no | n.a. |  | #_S3_8 (P); rr: N.R.  **perioperative Chemotherapie oder präoperative Radiochemotherapie bei operablen Patienten mit Adenokarzinom des Ösophagus**  Numerator:  Anzahl Patienten mit prä- und postoperativer Chemotherapie oder präoperativer Radiochemotherapie  Denominator:  Alle Patienten mit Adenokarzinom des #_int_1 (P); rr: N.R.  Ösophagus (C.16x, 8140/3) und Operation (OPS 5.422.0, alle 5.423, 5.424, 5.425, 5.426) und cT3 oder cT4 | yes^e^ | 8.24  Beim operablen Patienten mit Adenokarzinom des Ösophagus oder des ösophagogastralen Übergangs der Kategorie cT3 und bei resektablen cT4 Tumoren soll eine perioperative Chemotherapie oder eine präoperative Radiochemotherapie durchgeführt werden (GoR: A, LoE: 1a). |
|  | #_int_12 (P); rr: N.R.  **Recurrent disease**  *Proportion of patients diagnosed with recurrent oesophageal cancer discussed at the multidisciplinary team meeting prior to any treatment (non-measurable indicator)* | yes^e^ | In patients with recurrent oesophageal cancer, treatment options should be discussed in the multidisciplinary team (GoR: strong, LoE: very low). |  | #_S3_9 (P); rr: N.R.  **Systemtherapie des metastasierten Ösophaguskarzinoms**  Numerator:  Anzahl Patienten mit systemischer Chemotherapie (Erstlinie)  Denominator:  Alle Patienten mit einem metastasierten Adenokarzinom des Ösophagus (C16.x, 8140/3,M1) | yes^e^ | 9.1  Patienten mit einem metastasierten oder lokal fortgeschrittenen, nicht kurativ behandelbaren Adenokarzinom des Ösophagus soll eine systemische Chemotherapie angeboten werden. Therapieziel ist die Verlängerung der Überlebenszeit und der Erhalt der Lebensqualität (GoR: A, LoE: 1a). |
|  | #_int_13 (O); rr: N.R.  **Generic indicators**  Five-year survival rates computed after the oesophageal cancer incidence date by combined stage. | no | n.a. |  | #_S3_10 (O); rr: N.R.  **Anastomoseninsuffizienz nach chirurgischer Resektion**  Numerator:  Anzahl Patienten mit Anastomoseninsuffizienz (ICD: K91.83 „Insuffizienzen von Anastomosen und Nähten nach OP an: Anus, Darm, Magen, Ösophagus, Rektum), die endoskopisch, interventionell oder operativ behandelt wurden  Denominator:  Alle Patienten mit Neoplasie des Ösophagus (D.00.1, C.15x, C16x) und Operation (chirurgische Resektion OPS 5.422.0, alle 5.423, 5.424, 5.425, 5.426) | no | n.a. |
|  | #_int_14 (O); rr: N.R.  **Generic indicators**  Proportion of patients diagnosed with an oesophageal cancer in a given year, surviving 5 years after incidence date. | no | n.a. |  | #_S3_11 (O); rr: N.R.  **Mortalität nach Operation**  Numerator:  Anzahl postoperativ verstorbene Patienten nach 30 Tagen  Denominator:  Alle Patienten mit Neoplasie des Ösophagus (D.00.1,C.15x, C16x) und Operation (chirurgische Resektion OPS 5.422.0, alle 5.423, 5.424, 5.425, 5.426) | no | n.a. |
|  | #_int_15 (P); rr: N.R.  **Generic indicators**  Numerator:  All patients with oesophageal cancer surgically treated in high-volume hospitals in a given year  Denominator:  All patients with oesophageal cancer surgically treated in a given year | no | n.a. |  | #_S3_12 (O); rr: N.R.  **Mortalität nach Operation**  Numerator:  Anzahl postoperativ verstorbene Patienten nach 90 Tagen  Denominator:  Alle Patienten mit Neoplasie des Ösophagus (D.00.1,C.15x, C16x) und Operation (chirurgische Resektion OPS 5.422.0, alle 5.423, 5.424, 5.425, 5.426) | no | n.a. |
|  | #_int_1 (P); rr: N.R.  **Staging**  Numerator:  Proportion of patients diagnosed with gastric cancer discussed at the MDT meeting.  Denominator:  All patients diagnosed with gastric cancer in a given year. | yes^e^ | All patients diagnosed with gastric cancer should be discussed at a multidisciplinary team meeting (GoR: strong, LoE: low). | 032/009OL 2012 | #_S3_1 (P); rr: N.R.  **Staging**  Numerator:  Anzahl Patienten mit konventioneller B-Bild-Sonographie und CT-Thorax und CT-Abdomen  Denominator:  Alle Patienten mit Karzinomen des Magens und des ÖGU und kurativer Therapieintention | yes^e^ | Rec 24  Eine Fernmetastasierung soll mittels konventioneller B-Bild-Sonographie, CT-Thorax und CT-Abdomen ausgeschlossen sein. (strong rec, consensus-based) |
|  | #_int_2 (P); rr: N.R.  **Staging**  Numerator:  Proportion of patients diagnosed with gastric cancer undergoing a CT thorax/abdomen.  Denominator:  All patients diagnosed with gastric cancer in a given year. | yes^e^ | In patients with newly diagnosed gastric cancer, CT scan of the chest and abdomen should always be performed (GoR: strong, LoE: low). |  | #_S3_2 (P); rr: N.R.  **Staging**  Numerator:  Anzahl Patienten mit Stadieneinteilung und histologischer Klassifikation nach aktueller TNM – Klass. der UICC  Denominator:  Alle Patienten mit operierten Karzinomen des Magens und des ÖGU | yes^e^ | Rec 38  Die Stadieneinteilung und histologische Klassifikation der Karzinome des gastroösophagealen Übergangs und des Magens soll nach der jeweils aktuellen TNM-Klassifikation der UICC erfolgen. (strong rec., consensus-based) |
|  | #_int_3 (P); rr: N.R.  **Treatment of mucosal cancer**  *Proportion of patients diagnosed with cT1a gastric cancer undergoing endoscopic mucosal resection/endoscopic submucosal dissection who had an en bloc resection (non-measurable indicator)* | no | n.a. |  | #_S3_3 (P); rr: N.R.  **Staging**  Numerator:  Anzahl von Patientinnen/Patienten mit vollständiger und standardisierter pathol.-anat. Begutachtung  Denominator:  Alle Patienten mit operierten Karzinomen des Magens und des ÖGU | yes^e^ | Rec 39  Die pathol.-anat. Begutachtung soll vollständig und in standarisierter Form vorgenommen werden. (strong rec, consensus-based) |
|  | #_int_4 (P); rr: N.R.  **Neoadjuvant treatment**  Numerator:  Proportion of patients with gastric cancer beyond the mucosa (T_2-4_ N_Any_ M_0_) who received neoadjuvant chemotherapy before their surgical intervention  Denominator:  Proportion of patients with gastric cancer beyond the mucosa (T_2-4_ N_Any_ M_0_) who underwent a surgical intervention. | yes^e^ | If after multidisciplinary discussion neoadjuvant treatment is considered for a locally-advanced gastric tumour, neoadjuvant chemotherapy is recommended (GoR: strong, LoE: moderate). |  | #_S3_4 (O); rr: N.R.  **Chirurgische Therapie**  Numerator:  Anzahl Patienten mit R0-Resektion (vollständige Entfernung des Tumors und der regionären Lymphknoten, hist. bestätigt tumorfreie Resektionsränder)  Denominator:  Alle Patienten mit Magenkarzinom und Operation mit kurativer Intention | yes^e^ | Statement 54  Ziel der kurativen Chirurgie des Magenkarzinoms ist die vollständige Entfernung des Tumors und der regionären Lymphknoten mit histologisch bestätigt tumorfreien proximalen, distalen und zirkumferentiellen Resektionsrändern (R0) (LoE: 1b). |
|  | #_int_5 (O); rr: N.R.  **Surgery**  *Proportion of surgically treated patients who had a R0 resection (non-measurable indicator)* | yes^e^ | Surgery for gastric cancer should aim at achieving an R0 resection (GoR: strong, LoE: low). |  | #_S3_5 (P); rr: N.R.  **Chirurgische Therapie**  Numerator:  Anzahl Patienten mit Entfernung der regionären Lymphknoten von Kompartiment 1 und 2 (D2)  Denominator:  Alle Patienten mit Operation von Karzinomen des Magens oder ÖGU in kurativer Intention | yes^e^ | Statement 56  Die Entfernung der regionären Lymphknoten von Kompartiment 1 und 2 (D2-Lymphadenektomie) stellt den Standard für die operative Behandlung in kurativer Intention dar (LoE: 1). |
|  | #_int_6 (O); rr: N.R.  **Surgery**  Numerator:  All patients with gastric cancer beyond the mucosa treated with gastrectomy in a given year dying within 30 days after surgery  Denominator  All patients with gastric cancer beyond the mucosa treated with gastrectomy in a given year. | yes^e^ | Surgical resection should be considered standard treatment for patients with resectable gastric cancer (GoR: strong, LoE: low). |  | #_S3_6 (O); rr: N.R.  **Chirurgische Therapie**  Numerator:  Anzahl Patienten mit Entfernung von mehr als 25 Lymphknoten  Denominator:  Alle Patienten mit D2-Lymphadenektomie ohne Splenektomie/Pankreaslinks-resektion | yes^e^ | Statement 57  Bei der D2-Lymphadenektomie ohne Splenektomie/Pankreaslinksresektion werden in der Regel mehr als 25 regionäre Lymphknoten entfernt und histopathologisch untersucht (LoE: 1). |
|  | #_int_7 (O); rr: N.R.  **Surgery**  *Mean number of resected/evaluated lymph nodes during gastrectomy (non-measurable indicator)* | no | n.a. |  | #_S3_7 (P); rr: N.R.  **Perioperative, neoadjuvante und adjuvante Therapie**  Numerator:  Anzahl der Patienten mit perioperativer (prä- und postop.) Chemotherapie  Denominator:  Alle Patienten mit lokalisiertem Magenkarzinom cT3 und resektablen T4 | yes^e^ | Rec 66  Bei lokalisierten Magenkarzinom der Kategorien uT3 und resektablen uT4a Tumoren soll/sollte eine perioperative Chemotherapie, d.h. präoperativ durchgeführt und postoperativ fortgesetzt werden (GoR: A/B, LoE: 1b) (*no consens regarding strength of rec*). |
|  | #_int_8 (O); rr: N.R.  **Surgery**  *Proportion of patients experiencing anastomotic leakage after gastrectomy (non-measurable indicator)* | no | n.a. |  | #_S3_8 (P); rr: N.R.  **Perioperative, neoadjuvante und adjuvante Therapie**  Numerator:  Anzahl von Patienten mit perioperativer Chemotherapie oder neoadjuvanter Radiotherapie  Denominator:  Alle Patienten mit lok. Adenokarzinom des ösophagogastralen Übergangs uT3 und resektable uT4 | yes^e^ | Rec 67  Beim lokalisierten Adenokarzinom des ösophago-gastralen Übergangs der Kategorien uT3 und resektablen uT4 Tumoren soll/sollte eine perioperative Chemotherapie oder eine neo-adjuvante Radiochemotherapie durchgeführt werden (GoR: A/B, LoE: 1b; 1a-1b, 1b-2b) (*no consens regarding strength of rec*). |
|  | #_int_9 (P); rr: N.R.  **Metastatic disease**  Numerator:  All patients diagnosed with metastatic gastric cancer in a given year that received a combination of at least 2 different chemotherapeutic agents within 1 month before and 3 months after incidence date.  Denominator:  All patients diagnosed with metastatic gastric cancer in a given year. | yes^e^ | In patients with locally advanced or metastatic cancer of the stomach with good performance status combination chemotherapy is recommended (GoR: strong, LoE: high). |  | #_S3_9 (P); rr: N.R.  **Perioperative, neoadjuvante und adjuvante Therapie**  Numerator:  Anzahl Patienten mit interdisziplinärer Entscheidung über weitere Therapie  Denominator:  Alle Patienten mit Nachweis eines allgemeinen Tumorprogresses unter neoadjuvanter Therapie | yes^e^ | Rec 73  Bei Nachweis eines allgemeinen Tumorprogresses soll die Entscheidung über die weitere Therapie interdisziplinär erfolgen. (strong rec, consensus-based) |
|  | #_int_10 (P); rr: N.R.  **Metastatic disease**  Numerator:  All patients with metastatic gastric cancer that died in a given year and had palliative support within 3 months before death.  Denominator:  All patients with metastatic gastric cancer that died in a given year. | no | n.a. |  | #_S3_10 (P); rr: N.R.  **Tumorgerichtete palliative Therapie**  Numerator:  Anzahl von Patienten, denen eine systemische Chemotherapie angeboten wurde  Denominator:  Alle Patienten mit nicht –resektablem Magenkarzinom /Karzinom des ÖGU und gutem Allgemeinzustand ohne Chemotherapie | yes^e^ | Rec 86  Patienten in gutem Allgemeinzustand soll eine systemische Chemotherapie angeboten werden. Therapieziel ist die Verbesserung des Überlebens und der Erhalt der Lebensqualität. Alter stellt keine Kontraindikation dar. (GoR: A, LoE: 1b) |
|  | #_int_11 (P); rr: N.R.  **Recurrent disease**  *Proportion of patients diagnosed with recurrent gastric cancer discussed at the multidisciplinary team meeting prior to any treatment (non-measurable indicator)* | yes^e^ | In patients with recurrent gastric cancer, treatment options should be discussed in the multidisciplinary team (GoR: strong, LoE: very low). |  | #_S3_11 (P); rr: N.R.  **Tumorgerichtete palliative Therapie**  Numerator:  Anzahl von qualitätsgesicherten Bestimmungen  Denominator:  Alle Patienten mit Bestimmung des HER-2-Status | yes^e^ | Rec 89  (Vor dem Einsatz einer palliativen medikamentösen Tumortherapie sollte der HER-2-Status als positiver prädiktiver Faktor für eine Therapie mit Trastuzumab bestimmt werden.)  Die histopathologische Bestimmung am Tumorgewebe soll qualitätsgesichert durchgeführt werden. (strong rec, consensus-based) |
|  | #_int_12 (O); rr: N.R.  **Generic indicators**  Five-year survival rates computed after the gastric cancer incidence date by combined stage. | no | n.a. |  | #_S3_12 (P); rr: N.R.  **Ernährung**  Numerator:  Anteil Patienten die postoperativ vor der Entlassung eine eingehende diätetische Beratung zu den erforderlichen Änderungen im Ernähungsverhalten erhalten  Denominator:  Alle Patienten nach Ösophagektomie oder Gastrektomie | yes^e^ | Rec 122  Postoperativ sollen Patienten nach Ösophagektomie oder Gastrektomie vor der Entlassung eine eingehende diätetische Beratung zu den erforderlichen Änderungen im Ernährungsverhalten sowie ggf. eine Schulung im Umgang mit einer vorhandenen Feinnadelkatheterjejunostomie erhalten. (Ernährungsmedizinische Verlaufskontrollen ggf. mit Wiederholung der Ernährungsberatung sollten regelmäßig erfolgen.) (strong rec, consensus-based) |
|  | #_int_13 (O); rr: N.R.  **Generic indicators**  Proportion of patients diagnosed with a gastric cancer in a given year, surviving 5 years after incidence date. | no | n.a. |  |  |  |  |
|  | #_int_14 (P); rr: N.R.  **Generic indicators**  Numerator:  All patients with gastric cancer surgically treated in high-volume hospitals in a given year  Denominator:  All patients with gastric cancer surgically treated in a given year | no | n.a. |  | #_S3_13 (P); rr: N.R.  **Nachsorge und Rehabilitation**  Numerator:  Anzahl von Patienten mit dokumentierter Empfehlung zu Vit. B12-Substitution (z.B.1000μg alle 3 Mo) im Arztbrief  Denominator:  Alle Patienten nach Gastrektomie | yes^e^ | Rec 129  Nach einer Gastrektomie soll eine regelmäßige Vit. B12 Substitution lebenslang durchgeführt werden. (strong rec, consensus-based) |
| SIGN melanoma 2017 | #_int_1 (P); rr: 90%  **Excision Biopsy**  Numerator:  Number of patients with cutaneous melanoma undergoing diagnostic excision biopsy who had this carried out by a skin cancer clinician.  Denominator:  All patients with cutaneous melanoma undergoing diagnostic excision biopsy.  (Exclusions: no exclusions) | yes^i^ | GPs should refer urgently all patients in whom melanoma is a strong possibility rather than carry out a biopsy in primary care (GPP). | 032/024OL 2016 | #_S3_1 (P); rr: N.R.  **Sicherheitsabstand (1 cm) bei radikaler Exzision**  Numerator:  Pat. mit radikaler Exzision mit Sicherheitsabstand 1 cm  Denominator:  Pat. mit einem primären, kutanen Melanom und kurativer radikaler Exzision bei einer Tumordicke ≤ 2 mm | yes^e^ | 3.7  Für das maligne Melanom soll unter kurativer Intention eine radikale Exzision mit den Sicherheitsabständen zum Tumorrand erfolgen, um lokale Rezidive des Tumors zu vermeiden (GoR: A, LoE: 1a) |
|  | #_int_2 (P); rr: 90%  **Pathology Reporting**  Numerator:  Number of patients with cutaneous melanoma undergoing diagnostic excision biopsy where the surgical pathology report contains a full set of data items (as defined by the current Royal College of Pathologists dataset).  Denominator:  All patients with cutaneous melanoma undergoing diagnostic excision biopsy.  (Exclusions: no exclusions) | no | n.a. |  | #_S3_2 (P); rr: N.R.  **Sicherheitsabstand (2 cm) bei radikaler Exzision**  Numerator:  Pat. mit radikaler Exzision mit Sicherheitsabstand 2 cm  Denominator:  Pat. mit einem primären, kutanen Melanom und kurativer radikaler Exzision bei einer Tumordicke > 2 mm | yes^e^ |  |
|  | #_int_3 (P); rr: 95%  **Multi-Disciplinary Team Meeting (MDT)**  Numerator:  Number of patients with cutaneous melanoma discussed at the MDT before definitive treatment (wide local excision, chemotherapy/SACT, supportive care and radiotherapy).  Denominator:  All patients with cutaneous melanoma.  (Exclusions: Patients who died before first treatment) | yes^i^ | All patients with a diagnosis of melanoma should be discussed at a specialist multidisciplinary team (MDT) meeting (GPP). |  | #_S3_3 (P); rr: N.R.  **Lokoregionale Lymphknoten-Sonographie**  Numerator:  Pat. mit lokoregionaler Lymphknoten-Sonographie  Denominator:  Pat. mit malignem Melanom ≥ IB–IIIC | yes^e^ | 3.28  Die lokoregionale Lymphknoten-Sonographie soll bei Patienten mit Primärdiagnose eines malignen Melanoms ab Tumorstadium Ib durchgeführt werden (GoR: A, LoE: 1a).  3.55  Die lokoregionale Lymphknoten-Sonographie soll bei Patienten mit Verdacht auf oder Nachweis von lokoregionaler Metastasierung (Stadium IIIB/IIIC) eines malignen Melanoms durchgeführt werden (GoR: A, LoE: 1a). |
|  | #_int_4 (P); rr: 95%  **Clinical Examination of Draining Lymph Node Basins**  Numerator:  Number of patients with cutaneous melanoma who undergo clinical examination of relevant draining lymph node basins as part of clinical staging.  Denominator:  All patients with cutaneous melanoma.  (Exclusions: no exclusions) | no | n.a. |  |  |  |  |
|  | #_int_5 (P); rr: 90%  **Sentinel Node Biopsy Pathology**  Numerator:  Number of patients with cutaneous melanoma undergoing SNB, where the SNB report contains a full set of data items (as defined by the current Royal College of Pathologists dataset).  Denominator:  All patients with cutaneous melanoma undergoing SNB.  (Exclusions: no exclusions) | no | n.a. |  | #_S3_4 (P); rr: N.R.  **Wächterlymphknoten-Biopsie**  Numerator:  Pat., bei denen die WLKB durchgeführt wird.  Denominator:  Pat. mit einem primären, kutanen Melanom mit einer Tumordicke ≥ 1 mm und ohne Hinweis auf lokoregionale oder Fernmetastasierung | yes^e^ | 3.35  Zur Stadienzuordnung soll die Wächterlymph-knoten-Biopsie ab einer Tumordicke von 1,0 mm und ohne Hinweis auf lokoregionale oder Fernmetastasierung durchgeführt werden (GoR: A, LoE: 1a). |
|  | #_int_6a (P); rr: 95%  **Wide Local Excisions**  Numerator:  Number of patients with cutaneous melanoma undergoing diagnostic excision biopsy who undergo a wide local excision.  Denominator:  All patients with cutaneous melanoma undergoing diagnostic excision biopsy.  (Exclusions: Patients who died before treatment) | no | n.a. |  |  |  |  |
|  | #_int_6b (P); rr: 95%  **Wide Local Excisions**  Numerator:  Number of patients with cutaneous melanoma undergoing partial biopsy who undergo a wide local excision.  Denominator:  All patients with cutaneous melanoma undergoing partial biopsy.  (Exclusions:  - Patients who died before treatment  - Patients who have also undergone diagnostic excision biopsy) | no | n.a |  | #_S3_5 (P); rr: N.R.  **Postoperative Radiatio**  Numerator:  Pat. mit Radiatio mit 50–60 Gy in konventioneller Fraktionierung (5 x 1,8–2,5 Gy/Woche)  Denominator:  Pat. mit malignem Melanom und postoperativer Radiatio des Lymphabflussge-bietes | yes^e^ | 3.71  Falls die Indikation zur Bestrahlung des Lym-phabflussgebietes gestellt wird, soll die Strah-lentherapie mit 50–60 Gy in konventioneller Fraktionierung (5 x 1,8–2,5 Gy/Woche) erfol-gen (GoR: A, LoE: 2b). |
|  | #_int_7a (P); rr: 95%  **Time to Wide Local Excision**  Numerator:  Number of patients with cutaneous melanoma undergoing wide local excision within 84 days of their diagnostic excision biopsy.  Denominator:  All patients with cutaneous melanoma undergoing diagnostic excision biopsy.  (Exclusions: Patients who have also undergone partial biopsy) | no | n.a. |  | #_S3_6 (P); rr: N.R.  **Adjuvante systemische Therapie**  Numerator:  Pat. mit adjuvanter systemischer Chemotherapie/Dacarbazin  Denominator:  Pat. mit malignem Melanom Stadium I–III | yes^e^ | 3.73  Dacarbazin soll in der adjuvanten Therapie des Melanoms nicht verabreicht werden (GoR: A, LoE: 1a). |
|  | #_int_7b (P); rr: 95%  **Time to Wide Local Excision**  Numerator:  Number of patients with cutaneous melanoma undergoing wide local excision within 84 days of their partial biopsy.  Denominator:  All patients with cutaneous melanoma undergoing partial biopsy.  (Exclusions: no exclusions) | no | n.a. |  | #_S3_7 (P); rr: N.R.  **Adjuvante Extremitätenperfusion**  Numerator:  Pat. mit adjuvanter Extremitätenperfusion  Denominator:  Pat. mit malignem Melanom Stadium I–IIIB | yes^e^ | 3.75  Eine adjuvante Extremitätenperfusion mit Melphalan soll in der adjuvanten Therapie des Melanoms nicht verabreicht werden (GoR: A, LoE: 1b). |
|  | #_int_8 (P); rr: 75%  **BRAF Status**  Numerator:  Number of patients with unresectable stage III or IV cutaneous melanoma who have their BRAF status checked.  Denominator:  All patients with unresectable stage III or IV cutaneous melanoma  (Exclusions: no exclusions) | no | n.a. |  | #_S3_8 (P); rr: N.R.  **LDH-Bestimmung**  Numerator:  Pat. mit LDH-Bestimmung  Denominator:  Pat. mit malignem Melanom Stadium IV | yes^e^ | 3.103  LDH soll als Teil der aktuellen AJCC-Klassifikation bei Patienten mit Verdacht auf bzw. Nachweis von Fernmetastasen bestimmt werden (GoR: A, LoE: 1b). |
|  | #_int_9 (P); rr: 95%  **Imaging for Patients with Advanced Melanoma**  Numerator:  Number of patients with stage III and IV cutaneous melanoma undergoing completion lymphadenectomy who undergo CT or PET CT prior to completion lymphadenectomy.  Denominator:  All patients with stage III and IV cutaneous melanoma undergoing completion lymphadenectomy.  (Exclusions: no exclusions) | no | n.a. |  | #_S3_9 (P); rr: N.R.  **Lokoregionale Lymphknoten-Sonographie in der Nachsorge**  Numerator:  Pat. mit lokoregionaler Lymphknoten-Sonographie  Denominator:  Tumorfreie Pat. in der Nachsorge bei malignem Melanom Stad. >= IB –IIIC | yes^e^ | 3.134  Lokoregionale Lymphknoten-Sonographie soll bei Melanompatienten ab Stadium IB in der Nachsorge erfolgen (GoR A:, LoE: 1a). |
|  | #_int_10 (P); rr: 60%  **Systemic Therapy**  Numerator:  Number of patients with unresectable stage III and IV cutaneous melanoma who undergo SACT Systemic Anti Cancer Therapy).  Denominator:  All patients with unresectable stage III and IV cutaneous melanoma.  (Exclusions: Patients who died before treatment) | no | n.a. |  | #_S3_10 (P); rr: N.R.  **Vorstellung Hauttumorboard**  Numerator:  Pat. mit Stadium IV, die in dem interdisziplinären Hauttumorboard vorgestellt werden  Denominator:  Pat. mit malignem Melanom Stad. IV | yes^e^ | 3.146  Patienten mit metastasiertem Melanom (ab Stadium III) sollen zur Abstimmung der weiteren Diagnostik und Therapie in einem interdisziplinären Hauttumorboard vorgestellt werden. Die Möglichkeit des Einschlusses in klinische Studien sollte in jedem Fall geprüft werden (strong rec, consensus-based) |
|  | #_int_11 (P); rr: 40%  **Access to Lymphoedema Service**  Numerator:  Number of patients with cutaneous melanoma undergoing groin block dissection who have been referred to a lymphoedema service.  Denominator:  All patients with cutaneous melanoma undergoing groin block dissection.  (Exclusions: no exclusions) | no | n.a |  |  |  |  |
| SIGN ovar 2013 | #_int_1 (P); rr: 90%  **Risk of Malignancy Index recorded in the patient notes**  Numerator:  Number of patients with FIGO Stage 1 epithelial ovarian cancer having RMI score recorded in their notes prior to any definitive surgical intervention.  Denominator:  All patients with FIGO Stage I epithelial ovarian cancer undergoing definitive surgical intervention.  (Exclusions:  • Patients presenting for surgery as an emergency.  • Patients who do not undergo ultrasound scan) | no | n.a. | 032/035OL 2013 | #_S3_1 (P); rr: N.R.  **Operatives Staging frühes Ovarialkarzinom**  Numerator:  Anzahl Pat. mit operativem Staging mit:  •Laparotomie  •Peritonealzytologie  •Peritonealbiopsien  •Adnexexstirpation beidseits  •Hysterektomie, ggf. extraperitoneales Vorgehen  •Omentektomie mind. infrakolisch  •bds. pelvine u. paraaortale Lymphonodektomie  Denominator:  Alle Pat. mit Erstdiagnose OC FIGO I–IIIA | yes^e^ | 7.1.  Ein optimales Staging soll folgende Operationsschritte umfassen:  •Längsschnittlaparotomie  •Inspektion und Palpation der gesamten Abdominalhöhle  •Peritonealzytologie  •Biopsien aus allen auffälligen Stellen  •Peritonealbiopsien aus unauffälligen Regionen  •Adnexexstirpation beidseits  •Hysterektomie, ggf. extraperitoneales Vorgehen  •Omentektomie mind. Infrakolisch  •Appendektomie (bei muzinösem/unklarem Tumortyp)  •bds. pelvine und paraaortale Lymphonodektomie (strong rec, consensus-based) |
|  | #_int_2 (P); rr: 90%  **Extent of disease assessed by Computed Tomography (CT) or Magnetic Resonance Imaging (MRI) prior to treatment**  Numerator:  Number of patients with epithelial ovarian cancer having a CT scan or MRI of the abdomen and pelvis carried out prior to starting treatment.  Denominator:  All patients with epithelial ovarian cancer.  (Exclusions:  • Patients who decline to undergo investigation.  • Patients presenting for surgery as an emergency.) | no | n.a. |  | #_S3_2 (O); rr: N.R.  **Intraoperative Tumorruptur**  Numerator:  Anzahl Pat. mit intraoperativer Tumorruptur  Denominator:  Alle Pat. mit Erstdiagnose eines OC FIGO IA o. IB | no | n.a. |
|  |  |  |  |  | #_S3_3 (O); rr: N.R.  **Makroskopisch vollständige Resektion fortgeschrittenes Ovarialkarzinom**  Numerator:  Anzahl Pat. mit makroskopisch vollständiger Resektion  Denominator:  Alle Pat. mit Erstdiagnose OC ≥ FIGO IIB und operativer Tumorentfernung | yes^e^ | 7.6  Das Ziel der Primär-OP soll eine makroskopisch vollständige Resektion sein (strong rec, consensus-based). |
|  | #_int_3 (P); rr: 95%  **Treatment planned and reviewed at a multi-disciplinary team meeting**  Numerator:  Number of patients with epithelial ovarian cancer discussed at the MDT (multidisciplinary team) before definitive treatment.  Denominator:  All patients with epithelial ovarian cancer  (Exclusions: Patients who died before first treatment.) | no | n.a. |  | #_S3_4 (P); rr: N.R.  **Operation fortgeschrittenes Ovarialkarzinom**  Numerator:  Anzahl Pat., deren definitive operative Therapie durch einen Gynäkoonkologen durchgeführt wurde.  Denominator:  Alle Pat. mit Erstdiagnose OC FIGO ≥IIB nach Abschluss der operativen Therapie | yes^e^ | 7.2  Im Falle einer unerwarteten Diagnose eines fortgeschrittenen Ovarialkarzinoms soll eine histologische Sicherung und Beschreibung der Ausbreitung erfolgen. Die definitive Behandlung soll dann durch einen Gynäkoonkologen in einer geeigneten Einrichtung erfolgen (GoR: A, LoE: 4) |
|  |  |  |  |  | #_S3_5 (P); rr: N.R.  **Postoperative Chemotherapie fortgeschrittenes Ovarialkarzinom**  Numerator:  Anzahl Pat. mit postoperativer Chemotherapie  Denominator:  Alle Pat. mit Erstdiagnose OC ≥ FIGO IIB und Chemotherapie | yes^e^ | 7.10  Als Therapiefolge soll die Primär-Operation gefolgt von einer Chemotherapie durchgeführt werden (GoR: A, LoE: 1+). |
|  | #_int_4a (P); rr: 95%  **Patients with early stage disease have an adequate staging operation**  Numerator:  Number of early stage (FIGO Stage 1) epithelial ovarian cancer patients having primary surgery involving TAH (Total Abdominal Hysterectomy), BSO (Bilateral Salpingo-Oophorectomy), omentectomy and washings.  Denominator:  All early stage (FIGO Stage 1) epithelial ovarian cancer patients undergoing primary surgery.  (Exclusions:  • Patients having fertility conserving surgery.  • Patients with risk of malignancy index <200.  • Patients presenting for emergency surgery) | no | n.a. |  | #_S3_6 (P); rr: N.R.  **Keine adjuvante Chemotherapie frühes Ovarialkarzinom**  Numerator:  Anzahl Pat. mit adjuvanter Chemotherapie  Denominator:  Alle Pat. mit Erstdiagnose OC FIGO IA, G 1 und komplettem operativem Staging | yes^e^ | 8.1  Bei Patientinnen mit Ovarialkarzinom im Stadium IA Grad 1 nach komplettem operativem Staging soll keine adjuvante Chemotherapie durchgeführt werden (GoR: A, LoE: 1+). |
|  |  |  |  |  | #_S3_7 (P); rr: N.R.  **Platinhaltige Chemotherapie frühes Ovarialkarzinom**  Numerator:  Anzahl Pat. mit einer platinhaltigen Chemotherapie  Denominator:  Alle Pat. mit Erstdiagnose OC FIGO IC o. IA/B mit Grad 3 | yes^e^ | 8.2  Patientinnen mit Ovarialkarzinom im Stadium IC oder IA/B und Grad 3 sollen eine platinhaltige Chemotherapie über 6 Zyklen erhalten (GoR: A, LoE: 1+). |
|  |  |  |  |  | #_S3_8 (P); rr: N.R.  **First-line-Chemotherapie fortgeschrittenes Ovarialkarzinom**  Numerator:  Anzahl Pat. mit 6 Zyklen First-Line Chemotherapie Carboplatin AUC 5 u. Paclitaxel 175mg/m2  Denominator:  Alle Pat. mit Erstdiagnose Ov-Ca >= FIGO IIB | yes^e^ | 8.5  Die First-Line Chemotherapie für Patientinnen mit fortgeschrittenem Ovarialkarzinom (IIb-IV) soll aus Carboplatin AUC 5 und Paclitaxel 175 mg/m2 über 3 h iv. für insgesamt 6 Zyklen alle 3 Wochen bestehen (GoR: A, LoE: 1++) |
|  |  |  |  |  | #_S3_9 (P); rr: N.R.  **Chemotherapie bei platin-resistenten u/o –refraktären Erstrezidiv**  Numerator:  Anzahl der Pat. mit nicht-platinhaltiger Monotherapie mit Pegylierten liposomalen Doxorubicin, Topotecan, Gemcitabine o. Paclitaxel wöchentlich  Denominator:  Alle Pat. mit platin-resistenten u/o –refraktären Erstrezidiv eines Ov-Ca´s und Erstrezidivchemotherapie außerhalb von klinischen Studien | yes^e^ | 9.4  Patientinnen mit platin-resistentem und/oder -refraktärem Ovarialkarzinomrezidiv sollen, wenn eine Indikation zur Chemotherapie besteht, eine nicht-platinhaltige Monotherapie erhalten:  Folgende Zytostatika können in Betracht gezogen werden:  •Pegyliertes liposomales Doxorubicin  •Topotecan  •Gemcitabine  •Paclitaxel wöchentlich (GoR: A, LoE: 1+) |
|  | #_int_4b (P); rr: 95%  **Patients with early stage disease have an adequate staging operation**  Numerator:  Number of early stage (FIGO Stage 1) epithelial ovarian cancer patients having primary surgery involving TAH, BSO, omentectomy and washings.  Denominator:  All early stage (FIGO Stage 1) epithelial ovarian cancer patients operated on by a gynaecological oncologist.  (Exclusions:  • Patients having fertility conserving surgery.  • Patients with risk of malignancy index <200.  • Patients presenting for emergency surgery) | no | n.a. |  | #_S3_10 (P); rr: N.R.  **Kombinationstherapie bei platinsensitiven Rezidiv**  Numerator:  Anzahl Pat. mit platinhaltiger Kombinationstherapie  Denominator:  Alle Pat. mit platinsensitiven Rezidiv eines Ov-Ca´s und Rezidivchemotherapie, außerhalb von klinischen Studien | yes^e^ | 9.5  Patientinnen mit platinsensitivem Ovarialkarzinomrezidiv sollen, wenn eine Indikation zur Chemotherapie besteht, eine platinhaltige Kombinationstherapie erhalten (strong rec, consensus-based).  Folgende Kombinationen können in Betracht gezogen werden:  Carboplatin / Gemcitabin /Bevacizumab  Carboplatin/Peg.lip.Doxorubicin  Carboplatin / Paclitaxel  Carboplatin / Gemcitabin |
|  |  |  |  |  | #_S3_11 (P); rr: N.R.  **Beratung Sozialdienst**  Numerator:  Anzahl Pat. mit Beratung durch den Sozialdienst  Denominator:  Alle Pat. mit Erstdiagnose OC und Behandlung in der Einrichtung | yes^e^ | 10.0  Patientinnen mit Ovarialkarzinom sollen über die Möglichkeiten rehabilitativer Maßnahmen sowie die Unterstützung durch die Sozialberatung informiert und nach individueller Abklärung des Bedarfs geeignete Maßnahmen angeboten werden (strong rec, consensus-based). |
|  | #_int_5a (O); rr: 30%  **No macroscopic residual disease following surgery for advanced disease**  Numerator:  Number of patients with advanced epithelial ovarian cancer (FIGO Stage 2 or higher) with no macroscopic residual disease following surgery.  Denominator:  All patients with advanced epithelial ovarian cancer (FIGO Stage 2 or higher) undergoing surgery.  (Exclusions: Patients with FIGO Stage 4 disease.) | no | n.a. |  |  |  |  |
|  | #_int_5b (O); rr: 60%  **No macroscopic residual disease following surgery for advanced disease**  Numerator:  Number of patients with advanced epithelial ovarian cancer (FIGO Stage 2 or higher) undergoing surgery with macroscopic residual disease < 1cm.  Denominator:  All patients with advanced epithelial ovarian cancer (FIGO Stage 2 or higher) undergoing surgery.  (Exclusions: Patients with FIGO Stage 4 disease.) | no | n.a. |  |  |  |  |
|  | #_int_6 (P); rr: 90%  **Histopathology reports are complete and support clinical decision-making**  Numerator:  Number of patients with epithelial ovarian cancer undergoing definitive cytoreductive surgery who have a complete pathology report that contains all data items as defined by the Royal College of Pathologists.  Denominator:  All patients with epithelial ovarian cancer undergoing definitive cytoreductive surgery.  (Exclusions: no exclusions) | no | n.a. |  |  |  |  |
|  | #_int_7a (P); rr: 100%  **Histo/cytological diagnosis prior to starting neo-adjuvant chemotherapy**  Numerator:  Number of patients having histo/cytological diagnosis of epithelial ovarian cancer recorded prior to starting chemotherapy.  Denominator:  All patients with epithelial ovarian cancer undergoing neo-adjuvant chemotherapy.  (Exclusions: Patients for whom paracentesis, image-guided biopsy or laparoscopy is considered not suitable.) | no | n.a. |  |  |  |  |
|  | #_int_7b (P); rr: 80%  **Histo/cytological diagnosis prior to starting neo-adjuvant chemotherapy**  Numerator:  Number of patients who have a diagnosis of epithelial ovarian cancer confirmed by histology prior to starting chemotherapy.  Denominator:  All patients with epithelial ovarian cancer having histo/cytological diagnosis recorded prior to starting neo-adjuvant chemotherapy.  (Exclusions: no exclusions) | no | n.a. |  |  |  |  |
|  | #_int_8a (P); rr: 75%  **Delayed primary surgery**  Numerator:  Number of patients with advanced epithelial ovarian cancer (FIGO Stage 3c or 4) undergoing delayed primary surgery after neo-adjuvant chemotherapy.  Denominator:  All patients with advanced epithelial ovarian cancer (FIGO Stage 3c or 4) having neo-adjuvant chemotherapy.  (Exclusions: no exclusions) | yes^i^ | The use of neoadjuvant chemotherapy in women with stage IIIc or IV ovarian cancer may be considered as an alternative to primary debulking surgery (GoR: A, LoE: 1^++^). |  |  |  |  |
|  | #_int_8b (P); rr: 65%  **Delayed primary surgery**  Numerator:  Number of patients with advanced epithelial ovarian cancer (FIGO Stage 3c or 4) undergoing delayed primary surgery with residual disease <1cm.  Denominator  All patients with advanced epithelial ovarian cancer (FIGO Stage 3c or 4) undergoing delayed primary surgery after neo-adjuvant chemotherapy.  (Exclusions: no exclusions) | no | n.a. |  |  |  |  |
|  | #_int_9 (P); rr: 90%  **First-line Chemotherapy**  Numerator:  Number of epithelial ovarian cancer patients who receive chemotherapy treatment involving either paclitaxel in combination with a platinum-based compound or carboplatin only  Denominator:  All epithelial ovarian cancer patients  (Exclusions:  • Patients with low-grade serous disease.  • Patients with FIGO stage 1a or 1b, low grade (G1) disease.  • Patients with Stage 1a clear cell tumours.  • Patients who decline chemotherapy treatment.) | yes^i^ | #_int_9:  First line chemotherapy treatment of epithelial ovarian cancer should include a platinum agent either in combination or as a single agent, unless specifically contraindicated (GoR: A, LoE: 1^++^).  Carboplatin is the platinum drug of choice in both single and combination therapy (GoR: A, LoE: 1^++^). |  |  |  |  |
| SIGN breast 2013 | #_int_1 (P); rr: N.R.  number of referrals to the Clinical Genetics Service of individuals who may have an inherited increased risk of breast cancer | no | n.a. | 032/045OL 2012 | #_S3_1 (P); rr: > 95 %  **Intraoperative Präparateradio-/-sonographie**  Numerator:  Operationen mit intraoperativem Präparatröntgen bei mammographischer Drahtmarkierung und Operationen mit intraoperativer Präparatsonographie bei sonographischer Drahtmarkierung  Denominator:  Operationen mit präoperativer Drahtmarkierung gesteuert durch Mammographie oder Sonographie | yes^e^ | Stag-6  Die präoperative Markierung soll bei nicht tastbaren Veränderungen grundsätzlich erfolgen. Der Nachweis einer adäquaten Resektion ist durch intraoperative Bildgebung zu erbringen (GoR: A, LoE: 3b). |
|  | #_int_2 (O); rr: N.R.  rates of local recurrence or second tumour | no | n.a. |  |  |  |  |
|  | #_int_3 (P); rr: N.R.  rates of mastectomy and breast reconstruction | no | n.a. |  |  |  |  |
|  | #_int_4 (O); rr: N.R.  outcomes of treatment, including treatment-related morbidity and mortality, disease-free survival and overall survival | no | n.a. |  |  |  |  |
|  |  |  |  |  | #_S3_2 (P); rr: < 5 %  **Axilläre Lymphknotenentfernung bei DCIS (Duktales Carcinoma in situ)**  Numerator:  Patientinnen mit axillärer Lymphknotenentnahme  Denominator:  Patientinnen mit Histologie „DCIS“ und abgeschlossener operativer Therapie bei Primärerkrankung und brusterhaltender Therapie | yes^e^ | Präinv-3  Eine Axilladissektion soll beim DCIS nicht durchgeführt werden. Eine Sentinel- Node-Biopsie soll nur dann durchgeführt werden, wenn eine sekundäre Sentinel-Node-Biopsie aus technischen Gründen nicht möglich ist (GoR: A, LoE: 1b). |
|  |  |  |  |  | #_S3_3 (O); rr: > 95 %  **Angabe Resektionsrand und Sicherheitsabstand**  Numerator:  Patientinnen mit endgültigem tumorfreiem Resektionsrand (R0) und ausreichendem Sicherheitsabstand  Denominator:  Patientinnen mit gesicherter Primärerkrankung und Histologie ”invasives Mammakarzinom und/oder DCIS” (Duktales Carcinoma in situ) und abgeschlossener operativer Therapie | yes^e^ | Präinv-3  Der Resektionsrand ist ein wichtiger prognostischer Faktor beim DCIS. Der tumorfreie Abstand zum Schnittrand soll mindestens 2 mm betragen, wenn eine postoperative Bestrahlungsbehandlung erfolgt (GoR: A, LoE: 2b).  Operativ-1  Basis der Therapie für alle nicht fortgeschrittenen Mammakarzinome ist die Tumorresektion in sano (R0-Status) (strong rec, consensus-based).  Operativ-1  Der Resektionsrandstatus hat einen prognostischen Effekt beim invasiven Mammakarzinom. Es besteht ein signifikanter Zusammenhang zwischen dem Resektionsrandstatus (positiv vs. knapp vs. negativ) und der Lokalrezidivrate (statement, LoE: 3a).  Patho-5.1  Zur Einschätzung des Erkrankungsverlaufs (Prognose) und der voraussichtlichen Wirkung systemischer Therapien (Prädiktion) sollen die Eigenschaften des Tumors und die Situation der Patientin dokumentiert werden.  Als Prognosefaktoren sollen erhoben werden:  a. pTNM-Status (Tumorgröße, axillärer Lymphknotenbefall, Fernmetastasierung) (GoR: A, LoE: 1a)  b. Resektionsrand (R-Klassifikation) und Sicherheitsabstände (GoR: A, LoE: 1b; evidence is stated)  c. histologischer Typ (GoR: A, LoE: 2b; evidence is stated)  d. Grading (GoR: A, LoE: 2a)  Als Prognosefaktoren sollten erhoben werden:  e. Lymphgefäß- und Blutgefäßeinbruch (Lx, Vx) (GoR: unklar, LoE: 2b)  f. Alter (consensus-based)  g. Beim nodal-negativen Mammakarzinom kann die Bestimmung der Tumorkonzentrationen von uPA und PAI-1 mittels ELISA weitere prognostische Informationen liefern (GoR: 0, LoE: 1a)  Für die adjuvante Therapie sollen folgende prädiktive Faktoren erhoben werden:  h. Östrogen-/Progesteronrezeptorstatus für eine endokrine Systemtherapie (GoR: A, LoE: 1a)  i. HER2/neu-Status für eine zielgerichtete Anti-HER2-Therapie (GoR: A, LoE: 1b)  j. Menopausenstatus für den Einsatz einer antiöstrogenen Therapie (GoR:A, LoE: 1c)  k. Der prognostische und prädiktive Wert des Proliferationsmarkers Ki-67 ist nicht ausreichend belegt. Außerhalb von Studien soll er daher nicht zur Subtypisierung ER-positiver Mammakarzinome (z. B. Ki-67 < 14 %: Luminal A; Ki-67 _ 14 %: Luminal B) als Entscheidungsgrundlage für die systemische Therapie klinisch angewendet werden (consensus-based).  l. Der Einsatz von Analysen der Genexpression – PCR-basiert oder mittels Microarrays – zur Beurteilung der Prognose oder des Therapieansprechens (Prädiktion) ist für den Routineeinsatz nicht ausreichend validiert und kann daher nicht empfohlen werden (consensus-based). |
|  |  |  |  |  | #_S3_4 (P); rr: > 95 %  **Durchgeführte Strahlentherapie nach BET (brusterhaltende Therapie)**  Numerator:  Patientinnen mit invasivem Karzinom und BET, die eine Radiatio der Brust erhalten haben  Denominator:  Patientinnen mit Primärerkrankung invasives Mammakarzinom und BET | yes^e^ | RT-1  Bei invasivem Karzinom soll eine Bestrahlung der betroffenen Brust nach brusterhaltender Operation durchgeführt werden (GoR: A, LoE: 1a). |
|  |  |  |  |  | #_S3_5 (P); rr: > 95 %  **Durchgeführte endokrine Therapie bei rezeptorpositivem Befund**  Numerator:  Patientinnen, die eine adjuvante endokrine Therapie erhalten haben  Denominator:  Steroidrezeptorpositive Patientinnen mit Primärerkrankung invasives Mammakarzinom | yes^e^ | Adj-6  Bei Patientinnen mit östrogen- und/oder progesteronrezeptorpositiven Tumoren ist eine endokrine Behandlung indiziert (GoR: A, LoE: 1a). |
|  |  |  |  |  | #_S3_6 (P); rr: > 95 %  **Trastuzumab-Therapie bei HER2 (Human Epidermal Growth Factor Receptor 2) -positivem Befund**  Numerator:  Alle Patientinnen, die eine (neo-) adjuvante Trastuzumab- Therapie über 1 Jahr erhalten haben  Denominator:  Alle HER2 –positiven (immunhistochemisch Score 3+ und/oder ISH-positiv) Patientinnen mit Primärerkrankung invasives Mammakarzinom >= pT1c | yes^e^ | Adj-17  Patientinnen mit HER2-überexprimierenden Tumoren mit einem Durchmesser 1 cm (immunhistochemisch Score 3+ und/oder ISH-positiv) sollen eine (neo-)adjuvante Behandlung mit Trastuzumab über 1 Jahr erhalten (GoR: A, LoE: 1b).  Adj-17  Die adjuvante Behandlung mit Trastuzumab sollte vorzugsweise simultan mit der Taxan-Phase der adjuvanten Chemotherapie begonnen werden (GoR: B, LoE: 2a).  Adj-17  Wenn die Indikation für eine Chemotherapie bei Tumoren < 10 mm vorliegt, sollte zusätzlich Trastuzumab gegeben werden (moderate rec, consensus-based) |
|  |  |  |  |  | #_S3_7 (P); rr: > 80 %  **Durchgeführte Chemotherapien bei steroidrezeptornegativen Tumoren**  Numerator:  Patientinnen, die eine Chemotherapie erhalten haben  Denominator:  Alle Patientinnen mit steroidrezeptornegativen Tumoren bei histologisch gesicherter Primärerkrankung invasives Mammakarzinom | yes^e^ | Adj-9  Alle Patientinnen mit rezeptornegativen Tumoren (pN0 und pN+) sollen eine adjuvante Chemotherapie erhalten (GoR: A, LoE: 1a). |
|  |  |  |  |  | #_S3_8 (P)¸ rr: > 95 %  **Indikationen zur Strahlentherapie der Brustwand nach Mastektomie**  Numerator:  Patientinnen, mit Bestrahlung der Brustwand nach Mastektomie  Denominator:  Patientinnen mit histologisch gesicherter Primärerkrankung invasives Mammakarzinom, die eine Mastektomie erhalten haben und Indikation zur Bestrahlung der Brustwand (T3/T4-Tumoren, R1/R2-Resektionen ohne Nachresektionsmöglichkeit oder pN+>3 LK) aufweisen. | yes^e^ | RT-4  Die postoperative Radiotherapie der Brustwand nach Mastektomie senkt das Risiko eines lokoregionalen Rezidivs (statement, LoE: 1a).  RT-4  Bei Patientinnen mit hohem Lokalrezidivrisiko wird auch das Gesamtüberleben verbessert (statement, LoE: 1a).  RT-4  Bei folgenden Situationen ist daher die nachfolgende Strahlentherapie der Brustwand nach Mastektomie indiziert:  - T3/T4 (GoR A, LoE 1a)  - pT3 pN0 R0 nur bei Vorliegen von sonstigen Risikofaktoren (Lymphgefäßinvasion, Grading G3, „close resection margin“, Prämenopausalstatus, Alter < 50 Jahre) (GoR B, LoE 2b)  - R1-/R2-Resektion und fehlender Möglichkeit der sanierenden Nachresektion (GoR A, LoE 1a)  - pN+ (> 3 Lymphknoten) (GoR A, LoE 1a) |
|  |  |  |  |  | #_S3_9 (P); rr: > 95 %  **Meldung von diagnostizierten Fällen von invasivem Mammakarzinom und/oder DCIS (Duktales Carcinoma in situ) an Krebsregister zur Qualitätssicherung**  Numerator:  Patientinnen, die an ein klinisches u./o. epidemiologisches Krebsregister gemeldet wurden  Denominator:  Alle Patientinnen mit histologisch gesicherter Primärerkrankung invasives Mammakarzinom und/oder DCIS | yes^e^ | Doku-1  Befunde, Behandlungen, primär und im Krankheitsverlauf, sowie relevante Verlaufsereignisse sollen von Kliniken, niedergelassenen Ärzten und Instituten, die die Versorgung tragen, dokumentiert, bei Bedarf jederzeit genutzt und regelmäßig ausgewertet werden (strong rec, consensus-based). |
| CTFPC prostate 2014 | #1_int_ (P) rr: N.R.  rates of PSA testing | yes^i^ | For men aged less than 55 years, we recommend not screening for prostate cancer with the prostate-specific antigen (PSA) test. (strong rec, low-quality evidence.).  For men aged 55–69 years, we recommend not screening for prostate cancer with the PSA test. (weak rec, moderate-quality evidence.)  For men 70 years of age and older, we recommend not screening for prostate cancer with the PSA test. (strong rec, low-quality evidence.) | 043/022OL 2014 | - | n.a. | n.a. |
|  | #2_int_ (P), rr: N.R.  rates of subsequent follow-up | no | n.a. |  |  |  |  |
|  | #3_int_ (P) rr: N.R.  the degree to which men who request screening were accurately informed of the risks and benefits of screening | no | n.a. |  |  |  |  |
| ICSI palliative 2013 | #_int_1 (P); rr: N.R.  Numerator:  Number of patients who have been screened for palliative care.  Denominator:  Number of patients with a diagnosis of a serious illness that includes but is not limited to:  • Pulmonary disease  • Cancer/neoplasm  • Liver disease  • Renal disease  • Neurological disorders:  - Stroke  - Parkinson's  - Amyotrophic lateral sclerosis  - Multiple sclerosis | yes^e^ | Annotation #1 | 128/001OL 2015 | #_S3_1 (O); rr: N.R.  **Reduktion Atemnot**  Numerator:  Anzahl Patienten mit Reduktion der Atemnot innerhalb von 48 h  Denominator:  Alle Patienten mit Diagnose „nicht heilbare Krebserkrankung“ (APV und SPV) mit mittlerer/starker Atemnot | yes^e^ | 5.3  Die wiederholte Beurteilung der Atemnot vor, während und nach einer symptomatischen Therapie soll Bestandteil der Erfassung sein (strong rec, consensus-based). |
|  | #_int_2 (S); rr: N.R.  Numerator:  Number of clinicians with education and training regarding palliative care concepts.  Denominator:  Number of clinicians who work with patients with a diagnosis of a serious illness that  includes but is not limited to:  • Pulmonary disease  • Cancer/neoplasm  • Liver disease  • Renal disease  • Neurological disorders:  - Stroke  - Parkinson's  - Amyotrophic lateral sclerosis  - Multiple sclerosis | yes^e^ | Annotation #2 |  | #_S3_2 (O); rr: N.R.  **Reduktion Schmerz**  Numerator:  Anzahl Patienten mit Reduktion des Schmerzes innerhalb von 48 h  Denominator:  Alle Patienten mit Diagnose „nicht heilbare Krebserkrankung“  (APV und SPV) mit mittlerem/starkem Schmerz | yes^e^ | 6.1  Schmerzanamnese und schmerzbezogene klinische Untersuchung sollen Bestandteil jeder Schmerzdiagnostik sein (strong rec, consensus-based). |
|  | #_int_3 (S); rr: N.R.  Numerator:  Number of clinicians with training in the use of scripting for palliative care discussions.  Denominator:  Number of clinicians who work with patients with a diagnosis of a serious illness that  includes but is not limited to:  • Pulmonary disease  • Cancer/neoplasm  • Liver disease  • Renal disease  • Neurological disorders:  - Stroke  - Parkinson's  - Amyotrophic lateral sclerosis  - Multiple sclerosis | yes^e^ | Annotation #2 |  | #_S3_3 (P); rr: N.R.  **Opiate und Laxantien**  Numerator:  Anzahl Patienten ohne Therapie mit osmotisch wirksamen und/oder stimulierenden Laxantien  Denominator:  Alle Patienten mit Diagnose „nicht heilbare Krebserkrankung“  (APV und SPV) mit Opiatmedikation | yes^e^ | 6.25  Laxantien zur Behandlung oder Vorbeugung von opioidbedingter Obstipation sollen routinemäßig verordnet werden (GoR A, LoE 1+).  7.7  In der medikamentösen Mono- oder Kombinationstherapie zur Behandlung einer Obstipation sollen osmotisch wirksame und/oder stimulierende Laxantien eingesetzt werden (GoR A, LoE 1-). |
|  |  |  |  |  | #_S3_4 (P); rr: N.R.  **Symptomassessment in der Sterbephase**  Numerator:  Anzahl Patienten mit Symptomassessment mit Hilfe eines validierten Screeninginstruments in den letzten 72 h vor Versterben  Denominator:  Alle verstorbenen Patienten (APV und SPV) | yes^e^ | 10.23  In der Sterbephase auftretende Angst soll regelmäßig evaluiert werden. Hierbei soll neben verbalen Äußerungen auf klinische Hinweise, wie z. B. Unruhe, Schwitzen, Mimik oder Abwehrreaktionen geachtet werden (strong rec, consensus-based). |
|  | #_int_4 (P); rr: N.R.  Numerator:  Number of patients who have been assessed for palliative care domains.  Denominator:  Number of patients with a diagnosis of a serious illness that includes but is not limited to:  • Pulmonary disease  • Cancer/neoplasm  • Liver disease  • Renal disease  • Neurological disorders:  - Stroke  - Parkinson's  - Amyotrophic lateral sclerosis  - Multiple sclerosis | yes^e^ | Annotations #3 to 9 |  | #_S3_5 (P); rr: N.R.  **Erfassung von Unruhe in der Sterbephase**  Numerator:  Anzahl Patienten mit Evaluation von Unruhe in den letzten 72 h vor Versterben  Denominator:  Alle verstorbenen Patienten (APV und SPV) | yes^e^ | 10.24  Bei Unruhe in der Sterbephase sollen die im Vordergrund stehenden auslösenden Ursachen bestimmt werden, z. B. Schmerz, Obstipation, Harnverhalt, Atemnot, Angst und/oder ein Delir (strong rec, consensus-based). |
|  | #_int_5 (P); rr: N.R.  Numerator:  Number of patients who have symptoms assessment documented in the medical record.  Denominator:  Number of patients with a diagnosis of a serious illness that includes but is not limited to:  • Pulmonary disease  • Cancer/neoplasm  • Liver disease  • Renal disease  • Neurological disorders:  - Stroke  - Parkinson's  - Amyotrophic lateral sclerosis  - Multiple sclerosis | yes^e^ | Annotations #3 to 9 |  | #_S3_6 (P); rr: N.R.  **Beenden von tumorspezifischen Maßnahmen in der Sterbephase**  Numerator:  Anzahl Patienten mit tumorspezifischen Maßnahmen (system. Th, Radioth.) innerhalb von 14 Tagen vor Versterben  Denominator:  Alle verstorbenen Patienten (APV und SPV) | yes^e^ | 10.31  Tumorspezifische Medikamente und Maßnahmen sollen in der Sterbephase beendet werden (strong rec, consensus-based). |
|  | #_int_6 (P); rr: N.R.  Numerator:  Number of patients who have the following identified/documented:  • A discussion of treatment options with risk and benefits to each option discussed.  • Patient goals such as needs, preferences, values, concerns and fears.  • Plan of care follows the patient across the care continuum (inpatient, outpatient, home care/public health nursing, etc.)  Denominator:  Number of patients with a diagnosis of a serious illness that includes but is not limited to:  • Pulmonary disease  • Cancer/neoplasm  • Liver disease  • Renal disease  • Neurological disorders:  - Stroke  - Parkinson's  - Amyotrophic lateral sclerosis  - Multiple sclerosis | yes^e^ | Annotations #3, 10 |  | #_S3_7 (P); rr: N.R.  **Beenden von medizinischen Maßnahmen in der Sterbephase**  Numerator:  Anzahl Patienten mit Dialyse oder Hämofiltration oder Beatmung innerhalb von 7 Tagen vor Versterben  Denominator:  Alle verstorbenen Patienten (APV und SPV) | yes^e^ | 10.32  Alle medizinischen, pflegerischen und physiotherapeutischen Maßnahmen, die nicht dem Therapieziel bestmöglicher Lebensqualität dienen, sollen in der Sterbephase nicht eingeleitet oder, falls sie im Vorfeld eingeleitet wurden, beendet werden: z. B. Beatmung, Dialyse/ Hämofiltration, Intensivtherapie, Lagerung zur Dekubitus- oder Pneumonieprophylaxe (strong rec, consensus-based). |
|  | #_int_7 (P); rr: N.R.  Numerator:  Number of patients who have a revised, documented care plan that addresses the domains of palliative care.  Denominator:  Number of patients with a diagnosis of a serious illness that includes but is not limited to:  • Pulmonary disease  • Cancer/neoplasm  • Liver disease  • Renal disease  • Neurological disorders:  - Stroke  - Parkinson's  - Amyotrophic lateral sclerosis  - Multiple sclerosis | yes^e^ | Annotations #3 to 9 |  | #_S3_8 (P); rr: N.R.  **Screening auf Depression**  Numerator:  Anzahl Patienten mit Screening auf Depression bei Therapieplanung  Denominator:  Alle Patienten mit Diagnose „nicht heilbare Krebserkrankung“ (APV und SPV) | yes^e^ | 8.2  Bei Patienten mit einer nicht heilbaren Krebserkrankung soll das Vorliegen einer Depression aktiv und regelmäßig geprüft werden (GoR A, LoE 4) |
|  | #_int_8 (P); rr: N.R.  Numerator:  Number of patients who have a revised symptom assessment in the medical record.  Denominator:  Number of patients with a diagnosis of a serious illness that includes but is not limited to:  • Pulmonary disease  • Cancer/neoplasm  • Liver disease  • Renal disease  • Neurological disorders:  - Stroke  - Parkinson's  - Amyotrophic lateral sclerosis  - Multiple sclerosis | yes^e^ | Annotations #3 to 9 |  | #_S3_9 (P); rr: N.R.  **Vorausschauende Versorgungsplanung**  Numerator:  Anzahl Patienten mit Gespräch über die vorausschauende Versorgungsplanung bei Therapieplanung  Denominator:  Anzahl Patienten mit Gespräch über die vorausschauende Versorgungsplanung bei Therapieplanung | yes^e^ | 9.19  Patienten mit einer nicht heilbaren Krebserkrankung sollen das Angebot einer vorausschauenden Versorgungsplanung erhalten (strong rec, consensus-based).  9.20  Die Gesprächsbegleitung zur vorausschauenden Versorgungsplanung soll frühzeitig im Verlauf sowie wiederholt bei wesentlichen Veränderungen von Befinden und Prognose angeboten werden (strong rec, consensus-based). |
|  | #_int_9 (P); rr: N.R.  Numerator:  Number of patients who have documentation in the medical record of a completed advance directive.  Denominator:  Number of patients with a diagnosis of a serious illness that includes but is not limited to:  • Pulmonary disease  • Cancer/neoplasm  • Liver disease  • Renal disease  • Neurological disorders:  - Stroke  - Parkinson's  - Amyotrophic lateral sclerosis  - Multiple sclerosis | yes^e^ | Annotations #3, 9 |  | #_S3_10 (P); rr: N.R.  **Screening mittels MIDOS und IPOS**  Numerator:  Anzahl Patienten mit Screening mittels validierter Tools (z. B. MIDOS oder IPOS) bei Therapieplanung  Denominator:  Alle Patienten mit Diagnose „nicht heilbare Krebserkrankung“ (APV und SPV) | yes^e^ | 11.4  Bei einer nicht heilbaren Krebserkrankung sollen die körperlichen, psychischen, sozialen und spirituellen Bedürfnisse sowie die Belastungen und Informationsbedürfnisse der Patienten und Angehörigen wiederholt und bei einer Änderung der klinischen Situation erfasst werden (strong rec, consensus-based) |
|  | #_int_10 (P); rr: N.R.  Numerator:  Number of patients who have a completed POLST form documented in the medical record.  Denominator:  Number of patients with a diagnosis of a serious illness that includes but is not limited to:  • Pulmonary disease  • Cancer/neoplasm  • Liver disease  • Renal disease  • Neurological disorders:  - Stroke  - Parkinson's  - Amyotrophic lateral sclerosis  - Multiple sclerosis | yes^e^ | Annotations #3, 9 |  |  |  |  |
|  |  | ***Annotations***  **#1**  Palliative care should begin at the time of diagnosis of a serious condition and continue through cure, or until death and then into the family's bereavement period (strong rec, low quality evidence).  **#2**   - Palliative care discussion or referral should be considered whenever a patient develops a serious illness (strong rec, low quality evidence). - Palliative care discussions should be included wherever a patient with a life-limiting illness presents, including the intensive care unit and the emergency department (strong rec, low quality evidence).   **#3**   - Clinicians should use a validated assessment tool to assess palliative care needs (strong rec, low quality evidence). - Care conferences with the patient, family and an interdisciplinary team are recommended on an ongoing basis to discuss patient's condition, course of illness, treatment options, goals and plan of care (strong rec, low quality evidence).   **#4**  The physical aspects of the patient's serious illness should be an integral component of the palliative care plan (strong rec, low quality evidence).  **#5**   - A cultural assessment should be an integral component of every palliative care plan (strong rec, low quality evidence). - Clinicians should follow the established best practices of utilizing professional medical interpreters when English is not a patient's first language or when there are gaps in understanding English (strong rec, low quality evidence).   **#6**  A psychological assessment should be an integral component of the palliative care plan (strong rec, low quality evidence).  **#7**  A social assessment should be an integral component of the palliative care plan (strong rec, low quality evidence).  **#8**   - A spiritual assessment should be an integral part of the palliative care plan (strong rec, low quality evidence). - Clinicians should utilize clinically trained chaplains as members of the interdisciplinary health care team to provide patient-centered spiritual care and support (strong rec, low quality evidence).   **#9**   - Clinicians should initiate or facilitate advance care planning for all adult patients and their families with regular review as the patient's condition changes (strong rec, low quality evidence). - Informed consent should be obtained for any treatment or plan of care from either a patient with decision-making capacity or an appropriate surrogate decision-maker (strong rec, low quality evidence). - Clinicians should recognize those patients who are receiving non-beneficial, low-yield therapy (strong rec, high quality evidence).   **#10**  Clinicians should engage in shared decision-making with the patient and/or their families when establishing or revising goals of care (strong rec, low quality evidence). | |  |  |  |  |
| NICE diabtypeI 2015 and  NICE diabtypeII 2016 | #_int_1 (P); rr: N.R.  NM59  The percentage of patients with diabetes who have a record of an albumin: creatinine ratio (ACR) test in the preceding 15 months. | yes^e^ | **NICE diabtypeI 20015: Rec 1.1.6**  At the time of diagnosis (or if necessary after the management of critically decompensated metabolism), the diabetes professional team should develop with and explain to the adult with type 1 diabetes a plan for their early care. To agree such a plan will generally require:   - medical assessment to:   - ensure security of diagnosis of type of diabetes  - ensure appropriate acute care is given when needed  - review and detect potentially confounding disease and medicines  - detect adverse vascular risk factors   - environmental assessment to understand:   - the social, home, work and recreational circumstances of the person and carers  - their preferences in nutrition and physical activity  - other relevant factors, such as substance use   - cultural and educational assessment to identify prior knowledge and to enable optimal advice and planning about:   - treatment modalities  - diabetes education programmes   - assessment of emotional state to determine the appropriate pace of education (strong rec).   The results of the assessment should be used to agree a future care plan. Some items of the initial diabetes assessment:   - acute medical history - social, cultural and educational history/lifestyle review - complications history/symptoms - long-term/recent diabetes history - other medical history/systems - family history of diabetes/cardiovascular disease - medication history/current medicines - vascular risk factors - smoking - general examination - weight/BMI - foot/eye/vision examination - urine albumin excretion/urine protein/serum creatinine - psychological wellbeing - attitudes to medicine and self-care - immediate family and social relationships and availability of informal support (strong rec).   **NICE diabtypeII 2016: Rec. 1.7.12**  For guidance on managing kidney disease in adults with type 2 diabetes, see the *NICE guideline on chronic kidney disease in adults.* | nvl/001d 2015 | #_S3_1 (P); rr: N.R.  **Risikofaktoren für Nierenerkrankungen bei Patienten mit Diabetes mellitus**  Numerator:  Anzahl der Patienten, bei denen Risikofaktoren für eine Nierenerkrankung erfasst sind.  Denominator:  Alle Menschen mit Diabetes. | yes^e^ | 2-1  Risikofaktoren für eine Nierenerkrankung sollen erfasst und soweit möglich behandelt werden (GoR ⇑⇑) |
|  | #_int_2 (P); rr: N.R.  NM74  The percentage of patients with diabetes who have had the following care processes performed in the preceding 12 months  • BMI measurement  • BP measurement  • HbA1c measurement  • Cholesterol measurement  • Record of smoking status  • Foot examination  • Albumin: creatinine ratio  • Serum creatinine measurement | yes^e^ | **NICE diabtypeI 2015: Rec 1.13.2**  Assess cardiovascular risk factors annually, including:   - albuminuria - smoking - blood glucose control - blood pressure - full lipid profile (including HDL and LDL cholesterol and triglycerides) - age - family history of cardiovascular disease - abdominal adiposity (strong rec).   **NICE diabtypeI 2015: Rec. 1.15.6**  Make visual acuity testing a routine part of eye screening programmes (strong rec).  **NICE diabtypeII 2016: Rec. 1.3.4**  Integrate dietary advice with a personalised diabetes management plan, including other aspects of lifestyle modification, such as increasing physical activity and losing weight (strong rec).  **NICE diabtypeII 2016: Rec. 1.4.1**  Measure blood pressure at least annually in an adult with type 2 diabetes without previously diagnosed hypertension or renal disease. Offer and reinforce preventive lifestyle advice (strong rec).  **NICE diabtypeII 2016: Rec. 1.5.2**  For guidance on the primary and secondary prevention of cardiovascular disease in adults with type 2 diabetes, see the *NICE guidelines on cardiovascular disease andmyocardial infarction*.  **NICE diabtypeII 2016: Rec. 1.6.1**  In adults with type 2 diabetes, measure HbA1c levels at:   - 3–6-monthly intervals (tailored to individual needs), until the HbA1c is stable on unchanging therapy - 6-monthly intervals once the HbA1c level and blood glucose lowering therapy are stable (strong rec).   **NICE diabtypeII 2016: Rec. 1.7.11**  For guidance on preventing and managing foot problems in adults with type 2 diabetes, see the *NICE guideline on diabetic foot problems.* |  | #_S3_2 (P); rr: N.R.  **Die Bestimmung des Serumkreatinins und Berechnung der glomerulären Filtrationsrate (eGFR) im Rahmen eines Screenings**  Numerator:  Anzahl der Patienten, bei denen die glomeruläre Filtrationsrate (eGFR) bestimmt wurde.  Denominator:  Alle Patienten mit Diabetes und vorliegender Albuminurie. | yes^e^ | 2-6  Die glomeruläre Filtrationsrate (eGFR) soll zur Einschätzung der Nierenfunktion benutzt werden (GoR ⇑⇑). |
|  | #_int_3 (P); rr: N.R  NM95  The percentage of patients with diabetes, on the register, with a diagnosis of nephropathy (clinical proteinuria) or micro-albuminuria who are currently treated with an ACE-I (or ARBs) | yes^e^ | **NICE diabtypeI 2015: Rec. 1.15.10**  For guidance on managing kidney disease in adults with type 1 diabetes, see the *NICE guideline on chronic kidney disease*.  **NICE diabtypeI 2015: Rec. 1.15.11**  Ask all adults with type 1 diabetes with or without detected nephropathy to bring in the first urine sample of the day ('early morning urine') once a year. Send this for estimation of albumin:creatinine ratio. Estimation of urine albumin concentration alone is a poor alternative. Serum creatinine should be measured at the same time (strong rec).  **NICE diabtypeII 2016: Rec. 1.4.7**  First-line antihypertensive drug treatment should be a once-daily, generic angiotensin-converting enzyme (ACE) inhibitor. Exceptions to this are people of African or Caribbean family origin, or women for whom there is a possibility of becoming pregnant (strong rec).  **NICE diabtypeII 2016: Rec. 1.4.8**  The first-line antihypertensive drug treatment for a person of African or Caribbean family origin should be an ACE inhibitor plus either a diuretic or a generic calcium-channel blocker (strong rec).  **NICE diabtypeII 2016: Rec. 1.4.10**  For a person with continuing intolerance to an ACE inhibitor (other than renal deterioration or hyperkalaemia), substitute an angiotensin II-receptor antagonist for the ACE inhibitor (strong rec). |  | #_S3_3 (P); rr: N.R.  **Spezifische therapeutische Maßnahmen – Pharmakotherapie der Hypertonie**  Numerator:  Anzahl der Patienten, die mit ACE-Hemmer oder AT1-Rezeptorantagonisten behandelt werden.  Denominator:  Alle Patienten mit diabetischer Nephropathie und Hypertonie. | yes^e^ | 4-13  Patienten mit diabetischer Nephropathie und Hypertonie sollen mit ACE-Hemmern behandelt werden, denn diese hemmen die Progression der Niereninsuffizienz effektiver als andere Antihypertensiva (GoR ⇑⇑).  4-14  Bei Unverträglichkeit von ACE-Hemmern sollen Patienten mit Niereninsuffizienz und Hypertonie mit AT1-Rezeptorantagonisten behandelt werden (GoR ⇑⇑). |
|  | #_int_4 (P); rr: N.R  CCG63  Admissions rates for renal replacement therapy in people with diabetes | yes^e^ | **NICE diabtypeI 2015: Rec 1.5.2**  Give adults with type 1 diabetes who choose to integrate increased physical activity into a more healthy lifestyle information about:   - appropriate intensity and frequency of physical activity - role of self-monitoring of changed insulin and/or nutritional needs - effect of activity on blood glucose levels (likely fall) when insulin levels are adequate - effect of exercise on blood glucose levels when hyperglycaemic and hypoinsulinaemic - (risk of worsening of hyperglycaemia and ketonaemia) - appropriate adjustments of insulin dosage and/or nutritional intake for exercise and - post-exercise periods, and the next 24 hours - interactions of exercise and alcohol - further contacts and sources of information (strong rec).   **NICE diabtypeII 2016: Rec. 1.5.2**  For guidance on the primary and secondary prevention of cardiovascular disease in adults with type 2 diabetes, see the *NICE guidelines on cardiovascular disease and myocardial infarction.* |  | #_S3_4 (P); rr: N.R.  **Nieren-/Pankreastransplantation bei Patienten mit Typ-1-Diabetes**  Numerator:  Anzahl der in einem Transplantationszentrum vorgestellten Patienten.  Denominator:  Alle Menschen mit Typ-1-Diabetes und fortgeschrittener Niereninsuffizienz (< 30 ml/min/1,73m²KO) ohne Dialyse. | yes^e^ | 4-46  Menschen mit Typ-1-Diabetes und fortgeschrittener Niereninsuffizienz (GFR< 30 ml/min/1,73 m2 KO) sollen bereits vor Eintritt der Dialysebehandlung zur kombinierten Nieren-/ Pankreastransplantation vorgesehen werden (GoR ⇑⇑). |
| SNS diabtypeI 2012 | #_int_1(O); rr: N.R  **Renal Transplantation**  Numerator:  Number of kidney transplants performed in people with DM1 in a given year.  Denominator:  Estimated population with DM1. | no | n.a. |  |  |  |  |
| NICE diabtypeI 2015 and  NICE diabtypeII 2016 | #_int_1 (P); rr: N.R.  NM27  The percentage of patients newly diagnosed with diabetes, on the register, in the preceding 1 April to 31 March who have a record of being referred to a structured education programme within 9 months after entry on to the diabetes register | yes^e^ | **NICE diabtypeI 2015: rec 1.3.1**  Offer all adults with type 1 diabetes a structured education programme of proven benefit, for example the DAFNE (dose-adjustment for normal eating) programme. Offer this programme 6–12 months after diagnosis (strong rec).  **NICE diabtypeII 2016: rec 1.2.1**  Offer structured education to adults with type 2 diabetes and/or their family members or carers (as appropriate) at and around the time of diagnosis, with Type 2 diabetes in adults: management annual reinforcement and review. Explain to people and their carers that structured education is an integral part of diabetes care (strong rec).  **NICE diabtypeII 2016: rec 1.2.2**  Ensure that any structured education programme for adults with type 2  diabetes includes the following components:   - It is evidence-based, and suits the needs of the person. - It has specific aims and learning objectives, and supports the person and their family members and carers in developing attitudes, beliefs, knowledge and skills to self-manage diabetes. - It has a structured curriculum that is theory-driven, evidence-based and resource-effective, has supporting materials, and is written down. - It is delivered by trained educators who have an understanding of educational theory appropriate to the age and needs of the person, and who are trained and competent to deliver the principles and content of the programme. - It is quality assured, and reviewed by trained, competent, independent assessors who measure it against criteria that ensure consistency. - The outcomes are audited regularly (strong rec). | nvl/001f 2012 | #_S3_1 (P); rr: N.R.  Numerator:  Anzahl der Patientinnen/Patienten, für die das Angebot eines strukturierten Schulungsprogrammes unmittelbar nach Diagnosestellung des Diabetes dokumentiert ist  Denominator:  Alle Menschen mit neu diagnostiziertem Diabetes mellitus | yes^e^ | 2-1  Strukturierte Schulungsprogramme sollen jedem Menschen mit Diabetes mellitus sowie gegebenenfalls wichtigen Bezugspersonen (z. B. Angehörigen) unmittelbar nach Diagnosestellung des Diabetes und regelmäßig im Verlauf der Erkrankung als unverzichtbarer Bestandteil der Diabetesbehandlung angeboten werden (GoR ⇑⇑).  (#_S3_1, #_S3_2) |
|  | #_int_2 (P); rr: N.R.  NM28  The percentage of patients with diabetes who have a record of a dietary review by a suitably competent professional in the preceding 15 months | yes^e^ | **NICE diabtypeI 2015: rec 1.4.6**  Provide nutritional information individually and as part of a diabetes education programme (see section 1.3). Include advice from professionals with specific and approved training and continuing accredited education in delivering nutritional advice to people with health conditions. Offer opportunities to receive nutritional advice at intervals agreed between adults with type 1 diabetes and their advising professionals (strong rec).  **NICE diabtypeII 2016: rec 1.3.1**  Provide individualised and ongoing nutritional advice from a healthcare professional with specific expertise and competencies in nutrition (strong rec). |  | #_S3_2 (P); rr: N.R.:  Numerator:  Anzahl der Patientinnen/Patienten, für die das Angebot eines strukturierten Schulungsprogrammes nach Änderung des Therapieregimes im Verlauf der Erkrankung dokumentiert ist  Denominator:  Alle Menschen mit Diabetes mellitus | yes^e^ |  |
|  | #_int_3 (P); rr: N.R.  NM38  The percentage of patients with any or any combination of the following conditions: CHD, PAD, stroke or TIA, hypertension, diabetes, COPD, CKD, asthma, schizophrenia, bipolar affective disorder or other psychoses who are recorded as current smokers who have a record of an offer of support and treatment within the preceding 12 months | yes^e^ | **NICE diabtypeI 2015: rec 1.13.5**  Give adults with type 1 diabetes who smoke advice on smoking cessation and use of smoking cessation services, including NICE guidance-recommended therapies. Reinforce these messages annually for people who currently do not plan to stop smoking, and at all clinical contacts if there is a prospect of the person stopping (strong rec.).  (#_int_3, #_int_4)  **NICE diabtypeII 2016:** rec 1.3.10  For recommendations on lifestyle advice, see the NICE guidelines on: *preventing excess weight gain, weight management, obesity, physical activity, smoking: brief interventions and referrals, stop smoking services, smoking:harm reduction, and smoking: acute, maternity and mental health services*.  (#_int_3, #_int_4) |  | #_S3_3 (P); rr: N.R.:  Numerator:  Anzahl der Schulungen mit spezifischen Schulungsmaterialien  Denominator:  Alle durchgeführten strukturierten Schulungsprogramme für Menschen mit Diabetes | yes^e^ | 4-5  Zur didaktischen Unterstützung sollen spezifische Schulungsmaterialen wie z. B. Patientenhandbücher oder Arbeitsblätter verbindlicher Bestandteil von strukturierten Schulungsprogrammen sein (GoR ⇑⇑) |
|  | #_int_4 (P); rr: N.R.  NM39  The percentage of patients with any or any combination of the following conditions: CHD, PAD, stroke or TIA, hypertension, diabetes, COPD, CKD, asthma, schizophrenia, bipolar affective disorder or other psychoses who are recorded as current smokers who have a record of an offer of support and treatment within the preceding 15 months | yes^e^ |  |  |  |  |  |
|  | #_int_5 (P); rr: N.R.  CCG14  The proportion of adults with diabetes referred to a structured education programme within 12 months of diagnosis | yes^e^ | **NICE diabtypeI 2015: rec 1.3.1**  Offer all adults with type 1 diabetes a structured education programme of proven benefit, for example the DAFNE (dose-adjustment for normal eating) programme. Offer this programme 6–12months after diagnosis (strong rec).  **NICE diabtypeI 2015: rec 1.3.8**  Carry out more formal review of self-care and needs annually in all adults with type 1 diabetes. Vary the agenda addressed each year according to the priorities agreed between the healthcare professional and the adult with type 1 diabetes (strong rec).  **NICE diabtypeII 2016:** rec 1.1.1  Adopt an individualised approach to diabetes care that is tailored to the needs and circumstances of adults with type 2 diabetes, taking into account their personal preferences, comorbidities, risks from polypharmacy, and their ability to benefit from long-term interventions because of reduced life expectancy. Such an approach is especially important in the context of multimorbidity. Reassess the person's needs and circumstances at each review and think about whether to stop any medicines that are not effective (strong rec). |  |  |  |  |
| ICSI diabtypeII 2014 | #_int_1 (P); rr: N.R.  Numerator:  Number of patients who are advised about lifestyle modification and nutrition therapy within one year of diagnosis.  Denominator:  Number of patients ages 18-75 years old who have T2DM. | yes^i^ | **Nutrition Therapy**  A qualified health professional (which may include a clinician, dietitian, nursing staff and pharmacist) should provide nutrition therapy to a patient diagnosed with T2DM as part of a global treatment plan (GoR: strong, quality of evidence: moderate). |  |  |  |  |
| SNS diabtypeI 2012 | #_int_1 (P); rr: N.R.  **Training in diabetes education**  The evaluation will be carried out through a descriptive memoir of the training activities in diabetes education conducted in each Autonomous Community. Every two years, it will include as follows:  • Types of intervention performed.  • Routes and methods used.  • Target populations at which they are aimed.  • If any evaluation has been performed and the results achieved. | no | n.a. |  |  |  |  |
| ICSI backpain 2012 | #_int_1 (P); rr: N.R.  Numerator:  Number of patients who have following completed at the initial visit with the clinician:  1) pain assessment*, 2) functional status**, 3) patient history (including notation of presence or absence of "red flags"), 4) assessment of prior treatment and response, 5) job and activity association, and 6) psychosocial screening that includes depression and chemical dependency screening.  * Pain assessment can be done using the Visual Analog Scale, pain diagram or other assessment tool.  ** Functional assessment can be done using the Oswestry Disability Questionnaire or other assessment tool  Denominator:  Number of patients with diagnosis of acute low back pain or radiculopathy. | yes^e^ | Annotation #2a (2b enthält keine explizite Empfehlung) | nvl/007 2011 | #_S3_1 (P); rr: N.R.  **psychologische Risikofaktoren und Instrumente zur Früherkennung**  Numerator:  Anzahl von Patientinnen/Patienten für die psychosoziale Risikofaktoren systematisch erfasst werden  Denominator:  Alle Patientinnen/Patienten mit nach 4 Wochen andauerndem nichtspezifischem Kreuzschmerz trotz leitliniengerechter Maßnahmen | yes^e^ | 3-2  Dauern Schmerzen trotz leitliniengerechter Maßnahmen länger als 4 Wochen1 an, sollen psychosoziale Risikofaktoren schon in der primären ärztlichen Versorgung erfasst werden (GoR ⇑⇑) |
|  | #_int_2 (P); rr: N.R.  Numerator:  Number of patients who have following assessed at follow visit with the clinician: 1) pain assessment*, 2) functional assessment**, 3) clinician's objective assessment, and 4) psychosocial screening that includes depression and chemical dependency screening.  * Pain assessment can be done using the Visual Analog Scale, pain diagram or other assess-ment tool.  ** Functional assessment can be done using the Oswestry Disa-bility Questionnaire or other assessment tool  Denominator:  Number of patients with diagnosis of acute low back pain or radiculopathy. | yes^e^ | Annotation #2a |  | #_S3_2 (P); rr: N.R.  **bildgebende Verfahren bei akutem Kreuzschmerz**  Numerator:  Anzahl von Patientinnen/Patienten ohne Veranlassung/Durchführung bildgebender Diagnostik  Denominator:  Alle Patientinnen/Patienten mit akutem Kreuzschmerz und ohne Hinweise auf „red flags“ nach Anamnese und klinischer Untersuchung | yes^e^ | 3-5  Bei akutem Kreuzschmerz soll nach klinischem Ausschluss gefährlicher Verläufe durch Anamnese und körperliche Untersuchung keine bildgebende Untersuchung durchgeführt werden (GoR ⇓⇓). |
|  | #_int_3 (P); rr: N.R.  Numerator:  Number of patients for whom the clinician ordered imaging studies during the six weeks after pain onset, in the absence of "red flags."  Denominator:  Number of patients with non-specific back pain diagnosis. | yes^e^ | Annotations #11, 16, 17, 18 |  |  |  |  |
|  | #_int_4 (P); rr: N.R.  Numerator:  Number of patients who have repeat imaging studies in the absence of "red flags" or progressive symptoms.  Denominator:  Number of patients with diagnosis of non-specific back pain. | yes^e^ | Annotations #11, 16, 17, 18 |  | #_S3_3 (P); rr: N.R.  **Bewegung und Bewegungstherapie**  Numerator:  Anzahl von Patientinnen/Patienten, denen geraten wurde, körperliche Aktivität soweit wie möglich beizubehalten  Denominator:  Alle Patientinnen/Patienten mit akutem nichtspezifischem Kreuzschmerz (0-6 Wochen) | yes^e^ | 5-5  Patientinnen/Patienten sollen aufgefordert werden, körperliche Aktivitäten soweit wie möglich beizubehalten (GoR ⇑⇑). |
|  | #_int_5 (P); rr: N.R.  Numerator:  Number of patients for whom the clinician ordered imaging studies during the six weeks after pain onset.  Denominator:  Number of patients with radicular pain diagnosis. | yes^e^ | Annotation #28 |  | #_S3_4 (P); rr: N.R.  **Opioid-Analgetika**  Numerator:  Anzahl von Patientinnen/Patienten bei welchen die Opioidtherapie nach spätestens 3 Monaten reevaluiert wurde  Denominator:  Alle Patientinnen/Patienten mit chronischem Kreuzschmerz und 3 Monaten kontinuierlicher Opioidtherapie | yes^e^ | 6-11  Eine Reevaluation der Opioidtherapie soll bei akutem nichtspezifischem Kreuzschmerz nach spätestens 4 Wochen, bei chronischem nichtspezifischem Kreuzschmerz nach spätestens 3 Monaten erfolgen. Tritt die gewünschte Schmerzlinderung/Funktionsverbesserung nicht ein, ist die Fortsetzung der Opioidtherapie kontraindiziert (GoR ⇑⇑). |
|  | #_int_6 (P); rr: N.R.  Numerator:  Number of patients who were advised on maintenance or resumption of activities, against  bed rest, use of heat, education on importance of active lifestyle and exercise, and recommendation to take anti-inflammatory or analgesic medication in the first six weeks of pain onset in the absence of "red flags."  Denominator:  Number of patients with diagnosis of acute low back pain or radiculopathy. Exclusions: patients with "red flags." | yes^e^ | Annotations #11, 16, 17, 18, 31 |  | #_S3_5 (P); rr: N.R.  **Opioid-Analgetika**  Numerator:  Anzahl von Patientinnen/Patienten denen transdermale Opioide verordnet wurden  Denominator:  Alle Patientinnen/Patienten mit akutem (0-6 Wochen) oder subakutem (6-12 Wochen) Kreuzschmerz | yes^e^ | 6-14  Bei akutem oder subakutem nichtspezifischem Kreuzschmerz sollen transdermale Opioide nicht eingesetzt werden (GoR ⇓⇓). |
|  | #_int_7 (P); rr: N.R.  Numerator:  Number of patients who were prescribed opioids.  Denominator:  Number of patients with diagnosis of acute or subacute low back pain or radiculopathy. | yes^e^ | Annotations #11, 16, 17, 18 |  | #_S3_6 (P); rr: N.R.  **weitere intravenös verabreichte Medikamente**  Numerator:  Anzahl von Patientinnen/Patienten denen intravenös oder intramuskulär applizierbare Schmerzmittel, Glucocorticoide und Mischinfusionen verordnet wurden  Denominator:  Alle Patientinnen/Patienten mit nichtspezifischen Kreuzschmerzen | yes^e^ | 6-23  Intravenös oder intramuskulär applizierbare Schmerzmittel, Glucocorticoide und Mischinfusionen sollen für die Behandlung des akuten und chronischen nichtspezifischen Kreuzschmerzes nicht angewendet werden (GoR ⇓⇓). |
|  | #_int_8 (P); rr: N.R.  Numerator:  Number of patients who have their functional status assessed using the Oswestry Disability Questionnaire or other assessment tool.  Denominator:  Number of patients with diagnosis of acute or subacute low back pain or radiculopathy. | yes^e^ | Annotation #2a (9 auch keine Empfehlungen) |  | #_S3_7 (P); rr: N.R.  **invasive Therapieverfahren**  Numerator:  Anzahl von Patientinnen/Patienten die invasive Therapiemaßnahmen erhalten  Denominator:  Alle Patientinnen/Patienten mit nichtspezifischem Kreuzschmerz | yes^e^ | 7-1  Invasive Therapieverfahren sollen bei Patientinnen/Patienten mit nichtspezifischem Kreuzschmerz nicht eingesetzt werden (GoR ⇓⇓). |
|  | #_int_9 (P); rr: N.R.  Numerator:  Number of patients who have their pain status assessed using the Visual Analog Scale, pain diagram or other assessment tool.  Denominator:  Number of patients with diagnosis of acute or subacute low back pain or radiculopathy. | yes^e^ | Annotation #2a |  |  |  |  |
|  | #_int_10 (P); rr: N.R.  Numerator:  Number of patients who have had collaborative decision-making done with regards to referral to a specialist.  Denominator:  Number of patients with diagnosis of non-specific low back pain. | yes^e^ | Annotation #31 (40 keine Empfehlung) |  |  |  |  |
|  | #_int_11 (P); rr: N.R.  Numerator:  Number of patients who have had collaborative decision-making done regarding imaging,  intervention and/or surgery.  Denominator:  Number of patients with diagnosis radicular back pain. | yes^e^ | Annotation #31 |  |  |  |  |
|  |  | ***Annotations***  **#2a**  Clinicians should not recommend imaging (including computed tomography [CT], magnetic resonance imaging [MRI] and x-ray) for patients with non-specific low back pain (strong rec, moderate quality evidence).  **#11**   - Clinicians should educate patients as an adjunct to other treatment. No standardized form of education is suggested (strong rec, moderate quality evidence). - Non-steroidal anti-inflammatory drugs may be used for short-term pain relief in patients with acute and subacute low back pain (weak rec, moderate quality evidence). - Muscle relaxants may be used as an option in treating acute low back pain. However, possible side effects should be considered (weak rec, moderate quality evidence). - Cautious and responsible use of opioids may be considered for those carefully selected patients with severe acute pain not controlled with acetaminophen and NSAIDs, at a minimum effective dose for a limited period of time, usually less than one to two weeks (strong rec, low quality evidence). - Heat should be used for pain relief (strong rec, moderate quality evidence). - Cold therapy is not recommended for low back pain (weak rec, low quality evidence). - Clinicians should advise patients with acute and subacute low back pain to stay active and continue activities of daily living within the limits permitted by their symptoms (strong rec, moderate quality evidence). - Exercise should be recommended to reduce the recurrence of low back pain. However, no specific exercise is preferred (strong rec, moderate quality evidence). - Clinicians should not recommend bed rest for patients with low back pain (strong rec, moderate quality evidence). - Clinicians should not prescribe or recommend traction for the treatment of acute low back pain (weak rec, low quality evidence). - Clinicians should not recommend imaging (including computed tomography (CT), magnetic resonance imaging (MRI) and x-ray) for patients with non-specific low back pain (strong rec, moderate quality evidence).   **#16**   - Spinal manipulative therapy should be considered in the early intervention of low back pain (strong rec, moderate quality evidence). - At this point evidence is not sufficient to strongly recommend the clinical prediction rule. However, studies are currently underway that may add further support. Therefore, we suggest consideration of the clinical prediction rule in the category of early low back pain patients (weak rec, low quality evidence).   **#17**  Delayed-recovery assessment is not fully developed. However, much progress has been made, and it is recommended that the clinician use one or more approaches to identify a patient who is at risk and intervene with specific interventions (weak rec, low quality evidence).  **#18**   - Delayed-recovery risk assessment is not fully developed. However, much progress has been made, and it is recommended that the clinician use one or more approaches to identify a patient who is at risk and intervene with specific interventions (weak rec, low quality evidence). - Exercise is recommended in the treatment of subacute low back pain (strong rec, moderate quality evidence). - Spinal manipulative therapy should be considered in the early intervention of low back pain (strong rec, moderate quality evidence). - Clinicians should consider cognitive behavioral therapy in the treatment of subacute low back pain (weak rec, moderate quality evidence). - Acupuncture may be used as an adjunct treatment for subactue low back pain (weak rec, low quality evidence).   **#28**  Clinicians should not recommend imaging (including computed tomography [CT], magnetic resonance imaging [MRI] and x-ray) for patients in the first six weeks of radicular pain (strong rec, moderate quality evidence).  **#31**   - Imaging should be done to rule out underlying pathology or for those who are considering surgery, including epidural steroid injections (strong rec, moderate quality evidence). - Epidural steroid injections may be used for acute low back pain with a radicular component to assist with short-term pain relief (weak rec, moderate quality evidence). | |  |  |  |  |
| ICSI hypo 2014 | #_int_1 (P); rr: N.R.  Percentage of surgery patients for whom either active warming was used intraoperatively for the purpose of maintaining normothermia or who had at least one body temperature equal to or greater than 96.8ºF/36ºC recorded within the 30 minutes immediately prior to or the 15 minutes immediately after anesthesia end time. | no | n.a. | 001/018 2013 | #_S3_1 (P); rr: N.R.  Anteil Patienten mit dokumentierter Temperaturmessung prä- intra- und postoperativ unter allen chirurgischen Patienten in einer operativen Einrichtung | yes^i^ | **Allgemeine Empfehlungen zur perioperativen Körpertemperaturmessung**  1. Die Köperkerntemperatur sollte 1 -2 Stunden vor Beginn der Anästhesie durch die vorbereitende Organisationseinheit gemessen werden (consensus-based)  2. Intraoperativ sollte die Messung der Körperkerntemperatur kontinuierlich oder zumindest regelmäßig, alle 15 Minuten, erfolgen (consensus-based).  3. Eine Temperaturmessmöglichkeit sollte zur Grundausstattung jedes Anästhesiearbeitsplatzes gehören (consensus-based). |
|  |  |  |  |  | #_S3_2 (O); rr: N.R.  Normothermie (Postoperative Körperkerntemperatur 36°C) | no | n.a. |
|  |  |  |  |  | #_S3_3 (O); rr: N.R.  Vermeidung von shivering | no | n.a. |
|  |  |  |  |  | #_S3_4 (O); rr: N.R.  Verbesserung des patientenindividuellen Thermokomforts | no | n.a. |
| SIGN VTEPrev 2014 | #_int_1 (P); rr: N.R.  Compliance with and recording of risk assessment in all patients admitted to or presenting acutely at hospital. | yes^i^ | All patients admitted to hospital or presenting acutely to hospital should be individually assessed for risk of VTE and bleeding. The risks and benefits of prophylaxis should be discussed with the patient (GoR: D).  The risk assessment should be shared with the patient/carer and the outcome of that discussion formally recorded as part of the routine process of informed consent to treatment (GPP) (S. 9) | 003/001 2015 | #_S3_1 (P); rr: ≥ 95 %  Anteil der Patienten mit Angabe zur nahtlosen Fortführung oder Beendigung einer VTE-Prophylaxe im Entlassungsbrief an allen Patienten, die bis zum Zeitpunkt der Entlassung eine VTE-Prophylaxe erhalten haben. | yes^e^ | 2.7.2  Bei Notwendigkeit der Fortführung der Prophylaxe soll der weiterbehandelnde Arzt darüber informiert werden (strong rec, consensus-based) |
|  | #_int_2 (P); rr: N.R.  Compliance with appropriate prescription of mechanical and pharmacological prophylaxis. | yes^i^ ?? als no eingestuft!! | All patients admitted to hospital or presenting acutely to hospital should be individually assessed for risk of VTE and bleeding. The risks and benefits of prophylaxis should be discussed with the patient (GoR: D). |  |  |  |  |
|  | #_int_3a (P); rr: N.R.  Percentage of time in range for INR for patients receiving VKA | no | n.a. |  |  |  |  |
|  | #_int_3b (P); rr: N.R.  Percentage INR tests <1.5 and >4.5 as measures of likely poor efficacy and bleeding risk | no | n.a. |  |  |  |  |
|  | #_int_4 (P); rr: N.R.  The rate of healthcare-associated VTE should be recorded and monitored routinely to identify areas where the risk assessment policy may need to be reviewed. | no | n.a. |  |  |  |  |
|  | #_int_5 (P); rr: N.R.  National condition-specific audits should use available linked datasets to monitor readmission or death associated with a VTE episode. | no | n.a. |  |  |  |  |
| CCHMC VTE 2014 | #_int_1 (O); rr: N.R.  local surveillance data | no | n.a. |  | #_S3_2 (P); rr: ≥ 95 %  Anteil der Patienten mit dokumentiertem Aufklärungsgespräch über Nutzen, Risiko und Alternativen der prophylaktischen Maßnahmen an allen Patienten, die eine VTE-Prophylaxe erhalten | yes^e^ | 3.8  Die getroffene Risikoabschätzung einer VTE und die sich daraus ergebenden Maßnahmen der VTE-Prophylaxe müssen bezüglich Nutzen, Risiko und Alternativen mit dem Patienten im Rahmen eines Aufklärungsgespräches besprochen werden (§ 630 e Abs. 1 und 2 BGB) (GoR ⇑⇑) |
|  | #_int_2 (P); rr: N.R.  Successful preoperative patient assessment with appropriate application of recommended SCD prophylaxis for surgical patients. | no (oder Rec 1?) | n.a. |  |  |  |  |
|  | #_int_3 (P); rr: N.R.  Risk assessment and application of recommended prophylaxis for all eligible patients at time of admission. | yes^i^ | It is recommended that all patients age 10 to 17 years be assessed for VTE risk factors and, based on that assessment, assigned to a risk category (low, moderate or high) a. at the time of inpatient admission, and b. reassessed at 48 to 72 hours of hospitalization (LoE: 4a / 5) |  |  |  |  |
|  | #_int_4 (P); rr: N.R.  Risk assessment and application of recommended prophylaxis at 48 to 72 hours of hospitalization | yes^i^ |  |  |  |  |  |
| NICE menstrual bleeding 2016 | #_int_1 (O); rr: N.R.  CCG77  Rates of hysterectomy | yes^e^ | Rec 1.7.3  When surgery for fibroid-related HMB is felt necessary then UAE, myomectomy and hysterectomy must all be considered, discussed and documented (strong rec.).  Rec 1.7.5  Myomectomy is recommended for women with HMB associated with uterine fibroids and who want to retain their uterus (strong rec.).  Rec 1.7.6.  UAE is recommended for women with HMB associated with uterine fibroids and who want to retain their uterus and/or avoid surgery (strong rec.). | 015/070 2014 | #_S3_1a (O); rr: N.R.  Organverletzungen bei Hysterektomie:  Patientinnen mit mindestens einer Organverletzung bei Hyterektomie | no | n.a. |
|  |  |  |  |  | #_S3_1b (O); rr: N.R.  Organverletzungen bei Hysterektomie:  Patientinnen ohne Karzinom, Endometriose und Vor-OP mit mindestens einer Organverletzung bei Hysterektomie | no | n.a. |
|  |  |  |  |  | #_S3_2 (P); rr: N.R.  Indikation bei Hysterektomie | no | n.a. |
|  | #_int_2 (O); rr: N.R.  CCG78  Rates of endometrial ablation | yes^e^ | Rec 1.6.4  Endometrial ablation should be considered in women with HMB who have a normal uterus and also those with small uterine fibroids (less than 3 cm in diameter) (strong rec.).  Rec 1.6.5  In women with HMB alone, with a uterus no bigger than a 10-week pregnancy, endometrial ablation should be considered preferable to hysterectomy (strong rec). |  |  |  |  |
| NICE bipolar 2016 | #_int_1 (P); rr: N.R.  NM15  The percentage of patients with schizophrenia, bipolar affective disorder and other psychoses who have a record of alcohol consumption in the preceding 15 months. | yes^e^ | Rec 1.2.9  If bipolar disorder is managed solely in primary care, re-refer to secondary care if any one of the following applies:   - there is a poor or partial response to treatment - the person's functioning declines significantly - treatment adherence is poor - the person develops intolerable or medically important side effects from medication - comorbid alcohol or drug misuse is suspected - the person is considering stopping any medication after a period of relatively stable mood - a woman with bipolar disorder is pregnant or planning a pregnancy. (strong rec) | 038/019 2012 | #_S3_1 (P); rr: N.R.  **Wissensvermittlung**  Numerator:  % der Patienten, bei denen dokumentiert wurde, dass sie krankheitsspezifische Informationen zur Erkrankung und zur Therapie erhalten haben  Denominator:  alle Patienten | yes^e^ | Trialog 5  Patienten und Angehörige sollten auf eine mögliche Unterstützung in Form von Ratgebern, Selbsthilfemanualen, Schulungsprogrammen (z. B. Kommunikations-Trainings, Selbstmanagement-Trainings) hingewiesen werden, konkrete Literaturhinweise erhalten und zur Teilnahme an aktuellen Veranstaltungen ermuntert werden (moderate rec, consensus-based). |
|  | #_int_2 (P); rr: N.R.  NM16  The percentage of patients with schizophrenia, bipolar affective disorder and other psychoses who have a record of BMI in the preceding 15 months | yes^e^ | Rec 1.2.12  Ensure that the physical health check for people with bipolar disorder,  performed at least annually, includes:   - weight or BMI, diet, nutritional status and level of physical activity - cardiovascular status, including pulse and blood pressure - metabolic status, including fasting blood glucose, glycosylated haemoglobin (HbA1c) and blood lipid profile - liver function - renal and thyroid function, and calcium levels, for people taking long-term lithium. (strong rec) |  | #_S3_2 (P); rr: N.R.  **Selbsthilfe von Betroffenen**  Numerator:  % der Patienten, bei denen ein Hinweis auf eine Ermutigung zur Teilnahme an einer SHG dokumentiert wurde  Denominator:  alle Patienten | yes^e^ | Trialog 8  Betroffene und Angehörige sowie andere Bezugspersonen sollten zum Besuch von Selbsthilfegruppen ermutigt werden. Dabei ist die konkrete Nennung der (nächsten) Kontaktstellen (z. B. NAKOS, DGBS, weitere Angehörigenverbände) hilfreich.  Selbsthilfegruppen sollten als therapeutische Option mehr Beachtung finden. Neben der direkten Integration in das stationäre therapeutische Angebot ist auch eine kontinuierliche Kooperation mit regionalen Gruppen oder einer Kontaktstelle für Selbsthilfegruppen denkbar. Auf diese Weise können Selbsthilfegruppen als Element der Nachsorge zur Stabilisierung des Behandlungserfolgs genutzt werden (moderate rec, consensus-based). |
|  | #_int_3 (P); rr: N.R.  NM17  The percentage of patients with schizophrenia, bipolar affective disorder and other psychoses who have a record of blood pressure in the preceding 15 months | yes^e^ |  |  | #_S3_3 (P); rr: N.R.  **Familienselbsthilfe**  Numerator:  % der Patienten, bei denen mehrfach dokumentiert wurde, dass die Angehörigen eingebunden wurden  Denominator:  Patienten, die mit Einbindung der Angehörigen einverstanden waren | yes^e^ | Trialog11  Aufgrund der starken Dynamik der bipolaren Erkrankung sind die nahen Angehörigen oft in besonderer Weise belastet. Gleichzeitig gibt es häufiger nicht nur Primär- (d. h. Eltern/Geschwister), sondern auch Sekundärfamilien (d. h. Partner/Kinder/andere Bezugspersonen). Der Erhalt dieser Beziehungen hat große prognostische Bedeutung (statement).  Trialog 12  Angehörige sollten von Beginn an und über alle Phasen der Behandlung des Erkrankten einbezogen werden (moderate rec, consensus-based). |
|  | #_int_4 (P); rr: N.R.  NM21  The percentage of patients on lithium therapy with a record of serum creatinine and TSH in the preceding 9 months | yes^e^ | Rec 1.10.21  Measure the person's weight or BMI and arrange tests for urea and electrolytes including calcium, estimated glomerular filtration rate (eGFR) and thyroid function every 6 months, and more often if there is evidence of impaired renal or thyroid function, raised calcium levels or an increase in mood symptoms that might be related to impaired thyroid function. (strong rec)  Rec 1.10.22  Monitor lithium dose and plasma lithium levels more frequently if urea levels and creatinine levels become elevated, or eGFR falls over 2 or more tests, and assess the rate of deterioration of renal function. For further information see NICE's guidance on *chronic kidney disease* and *acute kidney injury*. (strong rec) |  | #_S3_4 (P); rr: N.R.  **Screening von Risikopersonen mit Verdacht auf das Vorliegen einer Bipolaren Störung**  Numerator:  % der Patienten, bei denen ein Screening auf das Vorliegen einer Bipolaren Störung im Lebenszeitverlauf dokumentiert wurde  Denominator:  alle Patienten | yes^e^ | Diagnostik 7  Es gibt validierte Screeninginstrumente zum Screening auf das Vorliegen einer Bipolaren Störung im Lebenszeitverlauf. Diese sind bislang jedoch wenig verbreitet. Ein vermehrter Einsatz bei Risikopersonen ist wünschenswert (statement). |
|  | #_int_5 (P); rr: N.R.  NM22  The percentage of patients on lithium therapy with a record of lithium levels in the therapeutic range within the previous 4 months | yes^e^ | Rec 1.10.20  After the first year, measure plasma lithium levels every 6 months, or every 3 months for people in any of the following groups:   - older people - people taking drugs that interact with lithium - people who are at risk of impaired renal or thyroid function, raised calcium levels or other complications - people who have poor symptom control - people with poor adherence - people whose last plasma lithium level was 0.8 mmol per litre or higher. (strong rec)   Rec 1.10.21  Measure the person's weight or BMI and arrange tests for urea and electrolytes including calcium, estimated glomerular filtration rate (eGFR) and thyroid function every 6 months, and more often if there is evidence of impaired renal or thyroid function, raised calcium levels or an increase in mood symptoms that might be related to impaired thyroid function. (strong rec)  Rec 1.10.22  Monitor lithium dose and plasma lithium levels more frequently if urea levels and creatinine levels become elevated, or eGFR falls over 2 or more tests, and assess the rate of deterioration of renal function. For further information see NICE's guidance on *chronic kidney disease* and *acute kidney injury*. (strong rec) |  | #_S3_5 (P); rr: N.R.  **Differentialdiagnostik**  Numerator:  % der Patienten, bei denen eine sorgfältige Prüfung des Vorliegens einer Bipolaren Störung dokumentiert wurde  Denominator:  Patienten mit Depression, bei denen mind. eine Risikofaktor vorlag | yes^e^ | Diagnostik 11  Beim Auftreten eines oder mehrerer der oben genannten Risikofaktoren bzw. Prädiktoren ist besonders sorgfältig zu prüfen, ob die Depression im Rahmen einer Bipolaren Störung auftritt (GoR: 0) |
|  | #_int_6 (P); rr: N.R.  NM130  The percentage of patients aged 18 years and over with schizophrenia, bipolar affective disorder and other psychoses who have a record of blood glucose or HbA1c in the preceding 12 months | yes^e^ | Rec 1.10.29  When prescribing valproate, be aware of its interactions with other anticonvulsants (particularly carbamazepine and lamotrigine) and with olanzapine and smoking (strong rec) |  | #_S3_6 (P); rr: N.R.  **Komorbidität**  Numerator:  % der Patienten, bei denen eine sorgfältige Untersuchung möglicher komorbider psychischer Störungen dokumentiert ist  Denominator:  Alle Patienten | yes^e^ | Diagnostik 21  Komorbide psychische Störungen sollten bei Bipolaren Störungen zu Beginn und im Verlauf der Erkrankung bei Bipolaren Störungen sorgfältig diagnostiziert und in Therapie und Verlaufsbeobachtung berücksichtigt werden (GoR B) |
|  | #_int_7 (P); rr: N.R.  NM108  The percentage of patients with schizophrenia, bipolar affective disorder and other psychoses who have a comprehensive care plan documented in the record, in the preceding 12 months, agreed between individuals, their family and/or carers as appropriate | yes^e^ | Rec 1.2.4  When working with people with bipolar disorder in primary care:   - engage with and develop an ongoing relationship with them and their carers - support them to carry out care plans developed in secondary care and achieve their recovery goals - follow crisis plans developed in secondary care and liaise with secondary care specialists if necessary - review their treatment and care, including medication, at least annually and more often if the person, carer or healthcare professional has any concerns. (strong rec)   Rec 1.3.4  If bipolar disorder is diagnosed, develop a care plan in collaboration with the person with bipolar disorder based on the assessment carried out in recommendation 1.3.2 as soon as possible after assessment and, depending on their needs, using the care programme approach. Give the person and their GP a copy of the plan, and encourage the person to share it with their carers. (strong rec)  Rec 1.9.4  When making transfer arrangements for a return to primary care, agree a care plan with the person, which includes:   - clear, individualised social and emotional recovery goals - a crisis plan indicating early warning symptoms and triggers of both mania and depression relapse and preferred response during relapse, including liaison and referral pathways - an assessment of the person's mental state - a medication plan with a date for review by primary care, frequency and nature of monitoring for effectiveness and adverse effects, and what should happen in the event of a relapse.   Give the person and their GP a copy of the plan, and encourage the person to share it with their carers. (strong rec) |  | #_S3_7 (P); rr: N.R.  **Komorbidität**  Numerator:  % der Patienten, bei denen eine sorgfältige Untersuchung möglicher komorbider somatischer Störungen zu Beginn und im Verlauf dokumentiert ist  Denominator:  Alle Patienten | yes^e^ | Diagnostik 23  Komorbide somatische Erkrankungen sollten zu Beginn und im Verlauf der Erkrankung bei Bipolaren Störungen sorgfältig diagnostiziert und in Therapie und Verlaufsbeobachtung berücksichtigt werden (moderate rec, consensus-based) |
|  | #_int_8 (P); rr: N.R.  NM120  The percentage of patients with schizophrenia, bipolar affective disorder and other psychoses aged 25-84 (excluding those with pre-existing CHD, diabetes, stroke and/or TIA) who have had a CVD risk assessment performed in the preceding 12 months (using an assessment tool agreed with NHS England) | yes^e^ | Rec 1.2.11  Monitor the physical health of people with bipolar disorder when responsibility for monitoring is transferred from secondary care, and then at least annually. The health check should be comprehensive, including all the checks recommended in recommendation 1.2.12 and focusing on physical health problems such as cardiovascular disease, diabetes, obesity and respiratory disease. A copy of the results should be sent to the care coordinator and psychiatrist, and put in the secondary care record (strong rec). |  | #_S3_8 (P); rr: N.R.  **Verlaufsdiagnostik**  Numerator:  % der Patienten mit wiederholter sorgfältiger Dokumentation des psychische Befindens im Verlauf der Erkrankung mit Hilfe bewährter Fremdbeurteilungsskalen seitens des Behandlers als auch mit Hilfe eines vom Patienten täglich ausgefüllten Stimmungstagebuchs  Denominator:  alle Patienten | yes^e^ | Diagnostik 24  Empfohlen wird die sorgfältige Dokumentation des psychischen Befindens des Patienten im Verlauf einer bipolaren Erkrankung mit Hilfe bewährter Fremdbeurteilungsinstrumente seitens des Behandlers als auch mit Hilfe eines vom Patienten möglichst täglich auszufüllenden Stimmungstagebuchs (strong rec, consensus-based). |
|  | #_int_9 (P); rr: N.R.  NM129  The percentage of patients aged 18 and over with schizophrenia, bipolar affective disorder and other psychoses who have a record of total cholesterol: hdl ratio in the preceding 12 months | yes^e^ | Rec 1.10.29  When prescribing valproate, be aware of its interactions with other anticonvulsants (particularly carbamazepine and lamotrigine) and with olanzapine and smoking (strong rec). |  | #_S3_9 (P); rr: N.R.  **Verlaufsdiagnostik**  Numerator:  % der Patienten mit wiederholter Dokumentation des psychosozialen Funktionsvermögens im Verlauf  Denominator:  alle Patienten | yes^e^ | Diagnostik 25  Empfohlen wird die sorgfältige Dokumentation des psychosozialen Funktionsvermögens des Patienten im Verlauf einer bipolaren Erkrankung, z. B. mit Hilfe bewährter Fremdbeurteilungsinstrumente (strong rec, consensus-based). |
|  | #_int_10 (P); rr: N.R.  NM38  The percentage of patients with any or any combination of the following conditions: CHD, PAD, stroke or TIA, hypertension, diabetes, COPD, CKD, asthma, schizophrenia, bipolar affective disorder or other psychoses who are recorded as current smokers who have a record of an offer of support and treatment within the preceding 12 months | no | n.a. |  | #_S3_10 (P); rr: N.R.  **Verlaufsdiagnostik**  Numerator:  % der Patienten, die Stimmungstagebuch führen  Denominator:  alle Patienten | yes^e^ | Diagnostik 26  Menschen mit Bipolaren Störungen wird empfohlen, Life-charts/Stimmungstagebücher zu führen und ihre Selbstwahrnehmung zu schulen, und diese Informationen für Gespräche untereinander und mit den Behandlern nutzen (strong rec, consensus-based). |
|  | #_int_11 (P); rr: N.R.  NM39  The percentage of patients with any or any combination of the following conditions: CHD, PAD, stroke or TIA, hypertension, diabetes, COPD, CKD, asthma, schizophrenia, bipolar affective disorder or other psychoses who smoke whose notes contain a record of an offer of support and treatment within the preceding 15 months | no | n.a. |  | #_S3_11 (P); rr: N.R.  **Somatische und laborchemische Diagnostik vor und während einer Pharmakotherapie**  Numerator:  % Patienten, bei denen vor Beginn die Bestimmung von Differentialblutbild, Elektrolyte, Leberenzyme, Nüchternglukosespiegel, Serum-Kreatinin, Schwangerschaftstest bei Frauen im gebärfähigen Alter, EKG dokumentiert wurde  Denominator:  Alle Patienten, bei denen eine Pharmakotherapie begonnen wurde | yes^e^ | Diagnostik 27  Die Erfassung und Bestimmung der folgenden Parameter vor Beginn einer Psychopharmakotherapie ist obligat: Differentialblutbild Elektrolyte Leberenzyme Nüchternglukosespiegel Serum-Kreatinin Blutdruck und Puls Körpergewicht und Körpergröße Schwangerschaftstest bei Frauen im gebärfähigen Alter Als apparative Diagnostik ein Elektrokardiogramm (EKG) (strong rec, consensus-based). |
|  |  |  |  |  | #_S3_12 (P); rr: N.R.  **Somatische und laborchemische Diagnostik vor und während einer Pharmakotherapie**  Numerator:  % Patienten, bei denen vor Beginn die wirkstoffspezifischen zusätzlichen Bestimmungen dokumentiert wurden  Denominator:  Alle Patienten, bei denen eine Pharmakotherapie mit diesen Wirkstoffen begonnen wurde | yes^e^ | Diagnostik 28  Individuell ist die Erfassung und Bestimmung der folgenden Parameter vor Beginn einer Psychopharmakotherapie zusätzlich indiziert.  Für Therapie mit:  - Lithium: TSH, T3, T4, SD-Sonographie (bei geplanter Langzeittherapie), Kreatinin-Clearance  - Valproat: Bilirubin, Lipase, Gerinnungshemmer (PPT und Quick oder PTT und INR)  - zugelassene Atypika: Cholesterin gesamt, LDL-Cholesterine, Triglyzeride. (strong rec, consensus-based) |
|  |  |  |  |  | #_S3_13 (P); rr: N.R.  **Somatische und laborchemische Diagnostik vor und während einer Pharmakotherapie**  Numerator:  % Patienten, bei denen wiederholt Medikamentenspiegel dokumentiert sind  Denominator:  Patienten, welche mit geeigneten Wirkstoffen behandelt werden (besonders Lithium, Valproat, Carbamazepin) | yes^e^ | Diagnostik 29  Das allgemeine Monitoring während einer Psychopharmakotherapie sollte abhängig von der Wirkstoffklasse und unter Berücksichtigung der pharmakokinetischen Eigenschaften auch eine regelmäßige Messung des Medikamentenspiegels (besonders bei Lithium, aber auch bei Valproat und Carbamazepin) beinhalten. Des Weiteren müssen die Verträglichkeit und Sicherheit der Pharmakotherapie erfasst werden (strong/moderate rec, consensus-based). |
|  |  |  |  |  | #_S3_14 (P); rr: N.R.  **Somatische und laborchemische Diagnostik vor und während einer Pharmakotherapie**  Numerator:  % der Patienten, bei denen im Behandlungsverlauf die Stoffwechsellage auf Hyperglykämie untersucht wurde  Denominator:  Patienten, die mit atyp. NL behandelt wurden | yes^e^ | Diagnostik 30  Bei der Behandlung mit atypischen Antipsychotika soll im Behandlungsverlauf ein Monitoring der Stoffwechsellage wegen möglicher hyperglykämischer und hyperlipidämischer Veränderungen erfolgen (strong rec, consensus-based) |
|  |  |  |  |  | #_S3_15 (P); rr: N.R.  **Somatische und laborchemische Diagnostik vor und während einer Pharmakotherapie**  Numerator:  % der Patienten, bei denen im Behandlungsverlauf die Stoffwechsellage auf Hyperlipidämie untersucht wurde  Denominator:  Patienten, die mit atyp. NL behandelt wurden | yes^e^ |  |
|  |  |  |  |  | #_S3_16 (P); rr: N.R.  **Somatische und laborchemische Diagnostik vor und während einer Pharmakotherapie**  Numerator:  % der Patienten, bei denen im Behandlungsverlauf bezüglich EPMS untersucht wurde  Denominator:  Patienten, die mit NL behandelt wurden | yes^e^ | Diagnostik 31  Bei der Behandlung mit Neuroleptika soll im Behandlungsverlauf auf das mögliche Auftreten extrapyramidal-motorischer Symptome geachtet werden (strong rec, consensus-based). |
|  |  |  |  |  | #_S3_17 (P); rr: N.R.  **Grundsätzliches**  Numerator:  % der Patienten, bei denen wiederholt Aufklärungen dokumentiert wurden  Denominator:  Alle Patienten | yes^e^ | Therapie-Grundsätzliches 3  Eine intensive, wiederholte Aufklärung des Patienten und mit Einverständnis des Patienten auch seiner Angehörigen über angeratene Pharmakotherapiemöglichkeiten soll erfolgen, auch um die Zusammenarbeit von Patient und Arzt zu verbessern. Wichtige Inhalte dieser Aufklärungsgespräche sind: Erläuterung der Ziele und Inhalte einer Akuttherapie und ggf. einer Phasenprophylaxe, Erörterung von Bedenken gegenüber den Medikamenten, Erläuterung biologischer Wirkmechanismen, Hinweis auf Wirklatenzen, Information über Wechselwirkungen und Nebenwirkungen, Erläuterung der angestrebten Behandlungsdauer bereits zu Beginn der Therapie. Bei medikamentöser Phasenprophylaxe sollte auch eine Aufklärung zu Langzeitnebenwirkungen erfolgen (strong/moderate rec, consensus-based) |
|  |  |  |  |  | #_S3_18 (P); rr: N.R.  **Grundsätzliches**  Numerator:  % der Patienten, bei denen wiederholt Gewichtsangaben dokumentiert wurden  Denominator:  Alle Patienten | yes^e^ | Therapie-Grundsätzliches 4  Regelmäßige Gewichtskontrollen sollten wegen einer möglichen Gewichtszunahme insbesondere bei Behandlung mit Mirtazapin, trizyklischen Antidepressiva, Lithium, Valproinsäure, Clozapin, Olanzapin, Quetiapin, Risperidon und Zotepin durchgeführt werden (moderate rec, consensus-based) |
|  |  |  |  |  | #_S3_19 (P); rr: N.R.  **Grundsätzliches**  Numerator:  % der Patienten, bei denen dokumentiert wurde, dass sie an einer störungsspezifischen Psychoedukationsgruppe teilgenommen haben (bzw. denen es zumindest angeboten wurde)  Denominator:  Alle Patienten | yes^e^ | Therapie-Grundsätzliches 5  Obwohl entsprechende qualitativ hochwertige Studien fehlen, spricht die klinische Erfahrung und Rückmeldung von Betroffenen dafür, Psychoedukationsgruppen problem- oder störungsspezifisch durchzuführen. Eine Vermischung von Patienten mit unterschiedlichen Erkrankungen wird nicht empfohlen (statement). |
|  |  |  |  |  | #_S3_20 (P); rr: N.R.  **Grundsätzliches**  Numerator:  % der Patienten, bei denen dokumentiert wurde, dass sie eine mindestens einfache Psychoedukation erhalten haben  Denominator:  Alle Patienten | yes^e^ | Therapie-Grundsätzliches 6  Auch wenn es keine klaren Wirksamkeitsnachweise gibt, sollte die einfache Psychoedukation das Minimum sein, das in jeder ärztlichen, psychologischen oder psychosozialen Behandlung mit Patienten mit Bipolaren Störungen durchgeführt wird. (statement). |
|  |  |  |  |  | #_S3_21 (P); rr: N.R.  **Grundsätzliches**  Numerator:  % der Patienten, denen kreative und handlungsorientierte Therapien angeboten wurden  Denominator:  Alle Patienten | yes^e^ | Therapie-Grundsätzliches 11  Unterstützende Therapieverfahren (wie z. B. Entspannungs-, Bewegungs, Ergo-, Kunst- oder Musik-/Tanztherapie) sollten Bestandteil des individuellen integrierten Behandlungsplans sein. Die spezifischen Behandlungsziele sollten in Absprache mit allen Beteiligten festgelegt und im Verlauf überprüft werden (moderate rec, consensus-based). |
|  |  |  |  |  | #_S3_22 (P); rr: N.R.  **Nicht-medikamentöse somatische Therapieverfahren**  Numerator:  % Patienten, bei denen eine EKT empfohlen wurde  Denominator:  Alle Patienten | yes^e^ | Therapie-Manie 29  Elektrokonvulsionstherapie (EKT) kann zur Behandlung schwerer manischer Episoden durchgeführt werden (GoR: 0).  Therapie-Manie 30  Elektrokonvulsionstherapie (EKT) sollte bei den seltenen Fällen, in denen eine pharmakotherapieresistente manische Episode vorliegt, durchgeführt werden (GoR: B) |
|  |  |  |  |  | #_S3_23 (P); rr: N.R.  **Behandlung Depression**  Numerator:  % Patienten, bei denen in den aufgezählten Intervallen nach Beginn der Pharmakotherapie Kontakte dokumentiert sind  Denominator:  alle Patienten, bei denen eine pharmakologische Behandlung bei akuter bipolarer Depression begonnen wurde | yes^e^ | Therapie-Depression 8  In den ersten vier Wochen der pharmakologischen Behandlung einer akuten bipolaren Depression sind Untersuchung und Gespräch mit dem Patienten mindestens wöchentlich angeraten, um Risiken und Nebenwirkungen der Pharmakotherapie zu erkennen, den Erfolg der eingeleiteten Maßnahmen beurteilen zu können und die Zusammenarbeit zwischen Patient und Arzt zu verbessern. Danach sind Intervalle von zwei bis vier Wochen, nach 3 Monaten bei ausreichender Stabilität eventuell längere Intervalle möglich. Je nach klinischer Situation können häufigere Frequenzen notwendig sein (strong rec, consensus-based). |
|  |  |  |  |  | #_S3_24 (P); rr: N.R.  **Behandlung Depression -**  **Nicht-medikamentöse somatische Therapieverfahren**  Numerator:  % Patienten, bei denen eine Lichttherapie empfohlen wurde  Denominator:  alle Patienten | yes^e^ | Therapie-Depression 36  Trotz spärlicher Evidenz Lichttherapie als nebenwirkungsarme Therapieoption mit guter Akzeptanz durch Patienten v. a. bei saisonalen Verläufen zusätzlich  E: 0 |
|  |  |  |  |  | #_S3_25 (P); rr: N.R.  **Behandlung Depression -**  **Nicht-medikamentöse somatische Therapieverfahren**  Numerator:  % Patienten, bei denen eine Wachtherapie empfohlen wurde  Denominator:  alle Patienten | yes^e^ | Therapie-Depression 37  Trotz spärlicher Evidenz bei der bipolaren Depression kann die Lichttherapie als nebenwirkungsarme Therapieoption mit guter Akzeptanz durch Patienten vor allem bei saisonalen Verläufen zusätzlich erwogen werden (GoR: 0). |
|  |  |  |  |  | #_S3_26 (P); rr: N.R.  **Behandlung Prophylaxe - Psychotherapie**  Numerator:  % Patienten, bei denen eine ausführliche und interaktive Gruppenpsychoedukation empfohlen wurde  Denominator:  alle Patienten | yes^e^ | Therapie-Prophylaxe 23  Zur rezidiv-prophylaktischen Behandlung einer Bipolaren Störung sollte eine ausführliche und interaktive Gruppenpsychoedukation durchgeführt werden (GoR: B) |
|  |  |  |  |  | #_S3_27 (P); rr: N.R.  **Behandlung Prophylaxe - Psychotherapie**  Numerator:  % stabile und weitestgehend euthyme Patienten, bei denen eine KVT empfohlen wurde  Denominator:  alle stabilen und weitestgehend euthymen Patienten | yes^e^ | Therapie-Prophylaxe 24  Eine rezidiv-prophylaktische Behandlung einer Bipolaren Störung mit einer manualisierten, strukturierten kognitiven Verhaltenstherapie kann bei aktueller Stabilität und weitgehend euthymer Stimmungslage empfohlen werden (GoR: 0). |
|  |  |  |  |  | #_S3_28 (P); rr: N.R.  **Behandlung Prophylaxe - Psychotherapie**  Numerator:  % Patienten, bei denen eine familienfokussierte Therapie empfohlen wurde  Denominator:  alle Patienten | yes^e^ | Therapie-Prophylaxe 25  Zur rezidiv-prophylaktischen Behandlung einer Bipolaren Störung kann eine familienfokussierte Therapie angeboten werden (GoR: 0). |
|  |  |  |  |  | #_S3_29 (P); rr: N.R.  **Behandlung Prophylaxe - Psychotherapie**  Numerator:  % Patienten, bei denen eine IPSRT weitergeführt wurde  N: Patienten, die in der akuten Phase eine IPSRT erhalten haben und bei denen eine langfristige, kontinuierliche Betreuung intendiert ist  Denominator:  N.R. | yes^e^ | Therapie-Prophylaxe 27  Zur rezidiv-prophylaktischen Behandlung einer Bipolaren Störung kann dann eine Interpersonelle und Soziale Rhythmustherapie fortgeführt werden, wenn sie bereits in der akuten Episode begonnen wurde und eine langfristige und kontinuierliche Betreuung intendiert ist (und keine zeitlich befristete Kurzzeittherapie) (GoR: 0). |
|  |  |  |  |  | #_S3_30 (P); rr: N.R.  **Rezidivprophylaxe bezüglich manischer Episoden**  Numerator:  % Patienten, bei denen eine ausführliche und interaktive Gruppenpsychoedukation empfohlen wurde  Denominator:  alle Patienten | yes^e^ | Therapie-Prophylaxe 29  Zur rezidiv-prophylaktischen Behandlung manischer Episoden einer Bipolaren Störung sollte eine ausführliche und interaktive Gruppenpsychoedukation durchgeführt werden (GoR: B). |
|  |  |  |  |  | #_S3_31 (P); rr: N.R.  **Rezidivprophylaxe bezüglich depressiver Episoden**  Numerator:  % Patienten, bei denen eine ausführliche und interaktive Gruppenpsychoedukation empfohlen wurde  Denominator:  alle Patienten | yes^e^ | Therapie-Prophylaxe 33  Zur rezidiv-prophylaktischen Behandlung von depressiven Episoden einer Bipolaren Störung sollte eine ausführliche und interaktive Gruppenpsychoedukation angeboten werden (GoR: B) |
|  |  |  |  |  | #_S3_32 (P); rr: N.R.  **Rezidivprophylaxe bezüglich depressiver Episoden**  Numerator:  % Patienten, bei denen eine kognitiv-verhaltenstherapeutische PT empfohlen wurde  Denominator:  alle Patienten | yes^e^ | Therapie-Prophylaxe 34  Wenn im Vordergrund eine rezidiv-prophylaktische Behandlung depressiver Episoden einer Bipolaren Störung steht, dann sollte eine kognitiv-verhaltenstherapeutische Psychotherapie angeboten werden (GoR: B). |
|  |  |  |  |  | #_S3_33 (P); rr: N.R.  **Rezidivprophylaxe bezüglich depressiver Episoden**  Numerator:  % Patienten, bei denen eine familienfokussierte PT empfohlen wurde  Denominator:  alle Patienten | yes^e^ | Therapie-Prophylaxe 35  Zur rezidiv-prophylaktischen Behandlung depressiver Episoden einer Bipolaren Störung kann eine Psychotherapie angeboten werden. Empirische Belege liegen für familienfokussierte Psychotherapie vor (GoR: 0). |
|  |  |  |  |  | #_S3_34 (P); rr: N.R.  **Behandlung Prophylaxe - Nicht-medikamentöse somatische Therapieverfahren**  Numerator:  % Patienten, bei denen eine ausführliche und interaktive Gruppenpsychoedukation empfohlen wurde  Denominator:  alle Patienten, bei denen eine erfolgreiche EKT zur Akutbehandlung durchgeführt wurde; v.a. wenn Patient nicht auf andere leitliniengerechte phasenprophylaktische Therapie anspricht | yes^e^ | Therapie-Prophylaxe 36  Nach erfolgreicher Durchführung einer Elektrokonvulsionstherapie (EKT) zur Akutbehandlung kann aufgrund klinischer Erfahrung zur Aufrechterhaltung des therapeutischen Erfolgs als Therapieoption auch eine Fortführung der EKT angeboten werden. Dies kommt vor allem dann in Frage, wenn Patienten auf eine andere leitliniengerechte phasenprophylaktische Therapie nicht angesprochen haben und eine Fortführung der EKT wünschen (GoR: 0). |
|  |  |  |  |  | #_S3_35 (P); rr: N.R.  **Behandlung Prophylaxe - Nicht-medikamentöse somatische Therapieverfahren**  Numerator:  % der Patienten, denen kreative und handlungsorientierte Therapien angeboten wurden  Denominator:  Alle Patienten | yes^e^ | Therapie-Prophylaxe 41  Obwohl empirische Untersuchungen spezifisch zu Bipolaren Störungen in ausreichender Qualität fehlen, legt die klinische Erfahrung nahe, dass kreative und handlungsorientierte Therapieverfahren wie beispielsweise Ergo-, Kunst- und Musik-/Tanztherapie im Rahmen eines ambulanten oder (teil-) stationären Behandlungskonzepts zur psychischen und sozialen Stabilisierung bipolarer Patienten beitragen können (statement). |
|  |  |  |  |  | #_S3_36 (P); rr: N.R.  **Suizidalität**  Numerator:  % Pat. für die wiederholt dokumentiert wurde, dass Suizidalität ggf. auch durch direkte Ansprache beurteilt wurde  Denominator:  alle Patienten | yes^e^ | Suizidalität 1  Aufgrund des besonders hohen Risikos muss der Behandler Suizidalität bei jedem Patientenkontakt klinisch einschätzen und ggf. direkt thematisieren, präzise und detailliert erfragen und vor dem Hintergrund der Anamnese früherer Suizidalität und vorhandener Eigenkompetenz und sozialer Bindungen beurteilen (strong rec, consensus-based) |
|  |  |  |  |  | #_S3_37 (P); rr: N.R.  **Suizidalität**  Numerator:  % Pat. welche Psychotherapie angeboten bekommen haben  Denominator:  alle suizidalen Patienten | yes^e^ | Suizidalität 13  Bei suizidgefährdeten Patienten soll eine Psychotherapie in Betracht gezogen werden, die zunächst auf die Suizidalität fokussiert. Das kurzfristige Ziel besteht dabei in intensiver Kontaktgestaltung und aktiver unmittelbarer Unterstützung und Entlastung bis zum Abklingen der Krise.  Bei suizidgefährdeten Patienten kann eine tragfähige therapeutische Beziehung per se suizidpräventiv wirken (strong rec, consensus-based). |
| CTFPHC obesity 2015 | #_int_1 (P); rr: N.R.  Proportion of adults with overweight or obesity (in particular those at risk of diabetes), in whom the weightloss interventions are offered or discussed | yes^i^ | […] Adults who are overweight or obese may be candidates for weight-loss treatment. (weak rec; very low-quality evidence) | 050/001 2014 | #_S3_1 (S); rr: N.R.  Schulungsraum | yes^e^ | Rec. 5.7.  Patienten mit Adipositas, bei denen die Indikation zur Behandlung in einer spezialisierten ambulanten oder stationären Einrichtung gegeben ist, sollten in Einrichtungen betreut werden, die besondere Qualitätskriterien erfüllen. Hierzu gehören Anforderungen an personelle Qualifikation, räumliche Ausstattung und Therapieprogramme. LoE 4 (Expertenkonsens); starker Konsens  (#_S3_1 to #_S3_12) |
|  | #_int_2 (P); rr: N.R.  proportion of adults with overweight or obesity (in particular those at risk of diabetes), who participate in structured programs | yes^i^ | • For adults who are obese (BMI 30–39.9) and are at high risk of diabetes, we recommend that practitioners offer or refer to structured behavioural interventions aimed at weight loss. (strong rec; moderate-quality evidence)  • For adults who are overweight or obese, we recommend that practitioners offer or refer to structured behavioural interventions‡ aimed at weight loss. (weak rec; moderate-quality evidence) |  |  |  |  |
|  | #_int_3 (O); rr: N.R.  proportion of adults with overweight or obesity (in particular those at risk of diabetes), who achieve weight loss | no | n.a. |  |  |  |  |
| NICE obesity 2014 | #_int_1 (P); rr: N.R.  NM121  The percentage of patients with coronary heart disease, stroke or TIA, diabetes, hypertension, peripheral arterial disease, heart failure, COPD, asthma and/ or rheumatoid arthritis who have had a BMI recorded in the preceding 12 months | yes^e^ | Rec 1.2.1  Use clinical judgement to decide when to measure a person's height and weight. Opportunities include registration with a general practice, consultation for related conditions (such as type 2 diabetes and cardiovascular disease) and other routine health checks (strong rec). |  |  |  |  |
|  | #_int_2 (S); rr: N.R.  NM128  The contractor establishes and maintains a register of patients aged 18 or over with a BMI ≥25 in the preceding 12 months | yes^e^ | Rec 1.2.2 Use BMI as a practical estimate of adiposity in adults. Interpret BMI with caution because it is not a direct measure of adiposity (strong rec).  Rec 1.2.7  Define the degree of overweight or obesity in adults using the following table:  Classifification BMI (kg/m2)  Healthy weight 18.5–24.9  Overweight 25–29.9  Obesity I 30–34.9  Obesity II 35–39.9  Obesity III 40 or more (strong rec). |  |  |  |  |
|  | #_int_3 (P); rr: N.R.  NM143  The percentage of patients aged 18 or over (on or after 1 April 2017) who have had a record of a BMI being calculated in the preceding 5 years (and after their 18th birthday) | yes^e^ | Rec 1.2.1  Use clinical judgement to decide when to measure a person's height and weight. Opportunities include registration with a general practice, consultation for related conditions (such as type 2 diabetes and cardiovascular disease) and other routine health checks (strong rec).  Rec 1.2.2.  Use BMI as a practical estimate of adiposity in adults. Interpret BMI with caution because it is not a direct measure of adiposity (strong rec). |  | #_S3_2 (S); rr: N.R.  Evtl. Lehrküche | yes^e^ |  |
|  |  |  |  |  | #_S3_3 (S); rr: N.R.  Arzt mit ernährungsmedizinischer Qualifikation | yes^e^ |  |
|  |  |  |  |  | #_S3_4 (S); rr: N.R.  Ernährungsfachkraft vorhanden | yes^e^ |  |
|  |  |  |  |  | #_S3_5 (S); rr: N.R.  DiabetesberaterIn DDG (bei übergewichtigen und adipösen Patienten mit Diabetes) | yes^e^ |  |
|  |  |  |  |  | #_S3_6 (S); rr: N.R.  Psychotherapeut/Psychotherapeutin (ärztlich/psychologisch) mit verhaltenstherapeutischer Expertise | yes^e^ |  |
|  |  |  |  |  | #7_S3_ (S); rr: N.R.  Physiotherapeut/Physiotherapeutin oder andere Berufsgruppe mit sportmedizinischer Qualifikation | yes^e^ |  |
|  |  |  |  |  | #_S3_8 (P); rr: N.R.  Medizinische Eingangsuntersuchung | yes^e^ |  |
|  |  |  |  |  | #_S3_9 (P); rr:N.R.  Strukturierte Schulung in Gruppen | yes^e^ |  |
|  |  |  |  |  | #_S3_10 (P); rr: N.R.  Integriertes, leitliniengerechtes Therapiekonzept mit mehreren Komponenten | yes^e^ |  |
|  |  |  |  |  | #_S3_11 (P); rr: N.R.  Therapiedauer 6-12 Monate | yes^e^ |  |
|  |  |  |  |  | # _S3_12 (P); rr: N.R.  Systematische Datendokumentation | yes^e^ |  |
|  |  |  |  |  |  |  |  |
| NICE weight 2014 | #_int_1 (P); rr: N.R.  NM121  The percentage of patients with coronary heart disease, stroke or TIA, diabetes, hypertension, peripheral arterial disease, heart failure, COPD, asthma and/ or rheumatoid arthritis who have had a BMI recorded in the preceding 12 months | yes^e^ | Rec 1  Adopt an integrated approach to preventing and managing obesity (strong rec).  Rec 6  Refer overweight and obese adults to a lifestyle weight management programme (strong rec). |  |  |  |  |
| ICSI obesity 2013 | #_int_1 (P); rr: N.R.  Numerator:  Number of patients age 18 years and older who have an annual BMI documented.  Denominator:  Total number of patients age 18 years and older in the clinic's primary care panel. | yes^e^ | Annotation 1  • Clinicians should calculate body mass index (BMI) for their patients on an annual basis for screening, and as needed for management. Classify BMI based on the National Institute of Health categories (see Table 3). Educate patients about their BMI and associated risks for them (strong rec, high quality evidence).  • Clinicians should consider waist circumference measurement to estimate disease risk for patients who have normal or overweight BMI scores. Refer to Table 2 for disease risk relative to weight and waist circumference (strong rec, moderate quality evidence).  • Clinicians need to carefully consider BMI and its associated mortality risk across different ethnicity, sex and age groups (strong rec, moderate quality evidence). |  |  |  |  |
|  | #_int_2 (P); rr: N.R.  Numerator:  Number of patients with a BMI ≥ 25 who receive education and counseling for weight management appropriate to their BMI level, including nutrition, physical activity, lifestyle changes, medication and/or surgical considerations.  • BMI 25-29.9: Lifestyle changes and behavioral management.  • BMI 30-34.9: Lifestyle changes, behavioral management and medication considerations.  • BMI 35-39.9: Lifestyle changes, behavioral management, medication therapy and surgical considerations.  • BMI 40+: Lifestyle changes, behavioral management, medication and surgical considerations.  • Percentage of patients with BMI ≥ 25 who set an individualized goal along with target date for reduction in BMI.  • Percentage of patients with BMI ≥ 25 who reach their goal BMI by the set target date.  Denominator:  Number of patients with a BMI ≥ 25. | yes^e^ | Annotation 8  Clinicians should use motivational interviewing techniques as a tool for encouraging behavior change (strong rec, moderate quality evidence).  (#_int_2 to #_int_6) |  |  |  |  |
|  | #_int_3 (O); rr: N.R.  Numerator:  Number of patients who have reduced their weight by 5%.  Denominator:  Number of patients with a BMI ≥ 25. | yes^e^ |  |  |  |  |  |
|  | #_int_4 (P); rr: N.R.  Numerator:  Number of patients with a BMI ≥ 25 who have 30 minutes of physical activity five times per week documented.  Denominator:  Number of patients with a BMI ≥ 25. | yes^e^ |  |  |  |  |  |
|  | #_int_5 (O); rr: N.R.  Numerator:  Number of patients with a BMI ≥ 25 who have reduced their BMI by 10%.  Denominator:  Number of patients with a BMI ≥ 25. | yes^e^ |  |  |  |  |  |
|  | #_int_6 (P); rr: N.R.  Numerator:  Number of patients with BMI > 40 who provided referrals to a bariatric specialist.  Denominator:  Number of patients with a BMI ≥ 40. | yes^e^ |  |  |  |  |  |
| NICE diabpreg 2015 | #_int_1 (P); rr: N.R.  NM70  The percentage of women with diabetes aged 17 or over and who have not attained the age of 45 who have a record of being given information and advice about pregnancy or conception or contraception tailored to their pregnancy and contraceptive intentions recorded in the preceding 12 months | yes^e^ | Rec 1.1.3  Give women with diabetes who are planning to become pregnant, and their family members, information about how diabetes affects pregnancy and how pregnancy affects diabetes. The information should cover:   - the role of diet, body weight and exercise - the risks of hypoglycaemia and impaired awareness of hypoglycaemia during - pregnancy - how nausea and vomiting in pregnancy can affect blood glucose control - the increased risk of having a baby who is large for gestational age, which increases the - likelihood of birth trauma, induction of labour and caesarean section - the need for assessment of diabetic retinopathy before and during pregnancy - the need for assessment of diabetic nephropathy before pregnancy - the importance of maternal blood glucose control during labour and birth and early feeding of the baby, in order to reduce the risk of neonatal hypoglycaemia - the possibility of temporary health problems in the baby during the neonatal period, which may require admission to the neonatal unit - the risk of the baby developing obesity and/or diabetes in later life (strong rec). | 057/023 2014 | # _S3_1 (S); rr: N.R.  **Diabetologie:**  Information, Beratung und Betreuung soll durch Fachärzte für Innere Medizin mit der Zusatzbezeichnung Diabetologie (übergangsweise Diabetologen DDG) erfolgen, die in ihrer ambulanten Einrichtung die erforderliche Strukturqualität vorhalten und eine Mindest- Betreuungsquote von 5 Fällen pro Jahr anstreben (GoR A). | n.a. | n.a. |
|  |  |  |  |  | # _S3_2 (S); rr: N.R.  **Gynäkologie/Geburtshilfe:**  Information, Beratung und Betreuung soll durch Fachärzte für Gynäkologie und Geburtshilfe erfolgen, die in ihrer ambulanten Einrichtung die erforderliche Strukturqualität vorhalten, und eine Mindest-Betreuungsquote von 5 Fällen pro Jahr anstreben (Härtegrad A). | n.a. | n.a. |
|  |  |  |  |  | # _S3_3 (S); rr: N.R.  **Entbindungskliniken:**  Die Entbindung soll in einem Perinatalzentrum LEVEL 2 oder LEVEL 1 bei Insulintherapie, bei diätetischer Therapie einer Schwangeren mit Typ 2 Diabetes Entbindungsklinik mit perinatalem Schwerpunkt, sofern keine erheblichen Begleiterkrankungen vorliegen, erfolgen (Bundesministerium für Gesundheit und Soziale Sicherung 2005 EK IV, Härtegrad A). | n.a. | n.a. |
| SNS diabtypeI 2012 | #_int_1 (O); rr: N.R.  **Percentages of complications in pregnancy, childbirth and puerperium**  Numerator:  Number of admissions due to complications related with DM1 which occurred during pregnancy, childbirth or puerperium.  Denominator:  Number total admissions of women with DM1 after any attention related to pregnancy, childbirth or puerperium. | no | n.a. |  |  |  |  |
| ICSI pain 2016 | #_int_1 (P); rr: N.R.  Numerator:  Number of patients who have documentation of the following at the visits: pain status assessment and functional assessment  Denominator:  Number of patients with visits for pain. | yes^e^ | Annotation #1 | 145/003 2014 | # _S3_1 (P); rr: N.R.  Anzahl der Patienten mit somatoformer Schmerzstörung (F45.40), die eine Verschreibung/Behandlung mit opioidhaltigen Analgetika erhalten | yes^e^ | Schmerzen bei funktionellen/somatoformen Störungen (ICD 10 F45.x) sollen nicht mit opioidhaltigen Analgetika behandelt werden (consensus-based) |
|  | #_int_2 (P); rr: N.R.  Numerator:  Number of patients who have a reassessment of their functional status within 12 weeks of initiating physical therapy.  Denominator:  Number of patients with chronic pain diagnosis, undergoing physical therapy. | yes^e^ | Annotation #10 |  | # _S3_2 (P); rr: N.R.  Anzahl der Patienten mit Fibromyalgiesyndrom (M79.70), die eine Verschreibung/Behandlung mit opioidhaltigen Analgetika außer Tramadol erhalten | yes^e^ | 20. Fibromyalgiesyndrom (ICD 10 M79.7)  a. Opioidhaltige Analgetika sollten beim Fibromyalgiesyndrom nicht als Therapieoption angeboten werden (LoE: 4a)  b. Tramadol und Tramadol /Paracetamol können als eine zeitlich befristete Therapieoption (4-12 Wochen) erwogen werden. (LoE: 2b) |
|  | #_int_3 (P); rr: N.R.  Numerator:  Number of patients with documentation of the following:  • Patient provider agreement  • Urine drug testing once in the past 12 months  • Risk assessment  • Patient education on the risks, side effects, and disposal of opioids  • PMP check twice in the past 12 months since the last visit  • Follow-up visits at least once a quarter  • Opioid MME dose/d documentation  - If opioid prescription, < 100 MME/d  - If concurrent benzodiazepines prescription, < 50 MME/d  • Taper or discontinuation of opioids at intervals of six months  • Naloxone prescription offered or on file  Denominator:  Number of patients with chronic pain diagnosis who are prescribed opioids. | yes^e^ | Annotation #13.8 |  |  |  |  |
|  | #_int_4 (P); rr: N.R.  Numerator:  Number of patients with new opioid prescriptions that are <= 20 pills or three days supply of short-acting opioid.  Denominator:  Number of patients with chronic pain diagnosis with a new opioid prescription (no opioid prescription for at least 90 days). Exclude patients with an opioid prescription for cancer, migraine and end-of-life care. | yes^e^ | Annotation #13.5 |  |  |  |  |
|  | #_int_5 (P); rr: N.R.  Numerator:  Number of patients with an opioid prescription that is long-acting where the following criteria for prescribing were met:  • History of tolerance is checked  • Medication adherence is verified  Denominator:  Number of patients with chronic pain diagnosis with an opioid prescription with a longacting formulation. Exclude patients with an opioid prescription for cancer, migraine and end-of-life care. | yes^e^ | Annotation #13.4 |  |  |  |  |
|  | #_int_6 (P); rr: N.R.  Numerator:  Number of patients with new opioid prescriptions where PMP (prescription monitoring program) is checked prior to prescribing.  Denominator:  Number of patients with new opioid prescriptions in dental, ED and urgent care settings. Exclude patients with an opioid prescription for cancer, migraine and end-of-life care. | yes^e^ | Annotation #13.8 |  |  |  |  |
|  |  | ***Annotations***  **#1**  Use validated tools to assess and document the patient’s functional status, quality-of-life and pain intensity (strength of rec. N.R.)  **#10**  Exercise should be a component of the treatment for a patient with chronic pain (strength of rec. N.R.).  Passive modalities should be performed only as an adjunct to a concomitant active physical therapy or exercise program (strength of rec. N.R.).  Extending physical therapy beyond 8-12 weeks for chronic pain patients should be based on objective clinical improvement (strength of rec. N.R.).  **#13.4**  **Patient Education and Shared Decision-Making**  The first opioid prescription should include patient education, shared decision-making and assessment for related risks (strength of rec. N.R.).  **Safe Use, Storage and Disposal**  Patients newly on opioids, or having recently had their opioid dose increased, should be advised not to operate heavy machinery, including driving a car, or participate in other work or home activity that may be affected by the sedating effect of opioids (strength of rec. N.R.).  An individualized approach that weighs the risks and benefits of driving and other activities should be taken with patients chronically on stable opioids who have tolerance and do not show evidence of sedation (strength of rec. N.R.).  Clinicians should discuss storage and opioid disposal options with patients at the first opioid prescription and in follow-up visits as needed (strength of rec. N.R.).  **Opioid Formulation**  Long-acting opioids should be reserved for patients with established opioid tolerance and in whom the prescriber is confident of medication adherence.  Long-acting tamper-proof formulation for opioids is preferred (strength of rec. N.R.).  **Patient-Provider Agreement (PPA)**  Initiate a patient-provider agreement (PPA) at the time an opioid is prescribed for:  • High-risk patients  • Daily use of opioids > 30 days  • Patient transfers to a new clinic already on opioids  • Episodic use up to 90 days over the course of a year  • If none of the above, initiate a PPA after 90 days of opioids is prescribed (strength of rec. N.R.).  **Consider Offering Naloxone**  Clinicians should consider offering the patient and close contacts (family/friends/caretaker) a naloxone kit (strength of rec. N.R.).  **13.5**  **Acute or Acute on Chronic Pain**  • The first opioid prescription for acute pain should be no more than 20 low-dose, short-acting opioids or three days of medication, whichever is less. The total dose for acute pain should not exceed 100 MME.  • For patients presenting in acute pain, already on chronic opioids, opioid tolerant or on methadone, use the same pill and dose limits as for opioid-naïve patients (strength of rec. N.R.).  **#13.8**  **Prescription Monitoring Program (PMP)**  The prescription monitoring program (PMP) should be queried in the following situations:  • If opioids are prescribed in dental, emergency department and urgent care settings, and when doses are changed.  • In every instance where there are concerns of substance use disorder, overdose, diversion, indeterminate pain disorder or polypharmacy.  • For those patients with an established stable dose of opioids for a chronically painful condition and a history of compliance with the prescriber, PMP checks should be at least twice per year.  Consider querying the PMP when initiating opioid therapy (strength of rec. N.R.).  **Urine Drug Screening**  • Routine random urine drug screens (UDS) for all patients on chronic opioid therapy for pain should be done at least once per year.  • UDS should be done if there is concern of aberrant behavior based on a prescriber’s assessments and clinical judgment (strength of rec. N.R.).  **Visit Frequency**  When initiating an opioid prescription, patients should be monitored within a month to evaluate harms and benefits, and assess treatment goals.  Patients on stable opioid doses should be seen every three months (strength of rec. N.R.).  **Referrals for High-Risk Patients**  Opioid prescribers should have a referral source for psychiatric treatment, substance use disorder treatment, physical therapy and pain medicine available if needed (strength of rec. N.R.). | |  |  |  |  |
| SIGN pain 2013 | #_int_1 (O); rr: N.R.  the number of patients presenting with chronic pain | no | n.a. |  |  |  |  |
|  | #_int_2 (P); rr: N.R.  the number of patients using analgesics to manage chronic pain who receive an annual review | yes^i^ | Patients using analgesics to manage chronic pain should be reviewed at least annually, and more frequently if medication is being changed, or the pain syndrome and/or underlying comorbidities alter (GCP) |  |  |  |  |
|  | #_int_3 (P); rr: N.R.  the number of patients on strong opioids and gabapentinoids who receive an annual review of their medications | yes^i^ | Strong opioids should be considered as an option for pain relief for patients with chronic low back pain or osteoarthritis, and only continued if there is ongoing pain relief. Regular review is required. (GoR: B) |  |  |  |  |
|  | #_int_4 (P); rr: N.R.  the number of patients on >180 mg/day morphine or equivalent referred for specialist assessment | yes^i^ | Specialist referral or advice should be considered if there are concerns about rapid-dose escalation with continued unacceptable pain relief, or if >180 mg/day morphine equivalent dose is required (GoR: D) |  |  |  |  |
|  | #_int_5 (P); rr: N.R.  the number of patients referred for self management | yes^i^ | Self management resources should be considered to complement other therapies in the treatment of patients with chronic pain (GoR: C). |  |  |  |  |

QI: quality indicator; N.R.: not reported; n.a: not applicable; GoR: grade of recommendation; LoE: level of evidence; rec: recommendation; S: structure-indicator; P: process-indicator; O: outcome-indicator
