# Supplementary material for: Guideline-based quality indicators—a systematic comparison of German and international clinical practice guidelines
Source: Implement Sci. 2019 Jul 9;14:71. doi: 10.1186/s13012-019-0918-y (PMC6617919; doi:10.1186/s13012-019-0918-y)
Supplement: Supplementary file 6 — Comparison of topic-related QIs in international CPGs and German S3-CPGs. (DOCX 37 kb) [file 13012_2019_918_MOESM6_ESM.docx]

**Additional file 6: Comparison of topic-related QIs in international CPGs and German S3-CPGs**

| **International CPG and corresponding German S3-CPG**  **[acronyms]** | **QI match [QI# international CPG + QI# corresponding German S3-CPG]** | | **No match** | |
| --- | --- | --- | --- | --- |
|  | QIs not different / slightly different [QI_int_#, QI_S3_#] | QIs different [QI_int_#, QI_S3_#] | QI only in international CPG  (QI_int_#) | QI only in German S3-CPG  (QI_S3_#) |
| CTFPHC colorectal 2016 and 021/007OL 2013 | n.a | n.a | #_int_1 to #_int_6 | - |
| SIGN colorectal 2016 and 021/007OL 2013 | - | - | #_int_1 to #_int_6 | #_S3_1 to #_S3_10 |
| KCE gastrointest 2012 and 021/023OL 2014 | #_int_1, #_S3_4 (P)  #_int_5, #_S3_6 (O)  #_int_6, #_S3_11 (O)  #_int_9, #_S3_10 (O) | - | #_int_2 to #_int_4, #_int_7, #_int_8, #_int_10 to #_int_15 | #_S3_1 to #_S3_3, #_S3_5, #_S3_7 to #_S3_9, #_S3_12 |
| KCE gastrointest 2012 and 032/009OL 2012 | #_int_2, #_S3_1 (P)  #_int_5, #_S3_4 (O)  #_int_7, #_S3_6 (O) | - | #_int_1, #_int_3, #_int_4, #_int_6, #_int_8 to #_int_14 | #_S3_2, #_S3_3, #_S3_5, #_S3_7 to #_S3_13 |
| SIGN melanoma 2017 and 032/024OL 2016 | #_int_4, #_S3_3 (P)  #_int_5, #_S3_4 (P)  #_int_6a, 6b #_S3_1, 2 (P) | #_int_3, #_S3_10 (P) | #_int_1, #_int_2, #_int_7a, #_int_7b, #_int_8 to #_int_11 | #_S3_5 to #_S3_9 |
| SIGN ovar 2013 and 032/035OL 2013 | #_int_4a, #_S3_1 (P)  #_int_5a, #_S3_3 (O) | #_int_9, #_S3_10 (P) | #_int_1, #_int_2, #_int_3, #_int_4b, #_int_5b, #_int_6, #_int_7a, #_int_7b | #_S3_2, #_S3_4 to #_S3_7, #_S3_9, #_S3_11 |
| SIGN breast 2013 and 032/045OL 2012 | - | - | #_int_1 to #_int_4 | #_S3_1 to #_S3_9 |
| CTFPHC prostate 2014 and 043/022OL 2014 | n.a. | n.a. | #_int_1 to #_int_3 | - |
| ICSI palliative 2013 and 128/001OL 2015 | #_int_5 , #_S3_4 (P)  #_int_7, #_S3_9 (P) | - | #_int_1 to #_int_4, #_int_6, #_int_8 to #_int_10 | #_S3_1 to #_S3_3, #_S3_5 to #_S3_8, #_S3_10 |
| NICE diabtypeI 2015 / NICE diabtypeII 2016 and nvl/001d 2015 | #_int_3, #_S3_3 (P)  #_int_4, #_S3_4 (P)  (two times) | - | #_int_1, #_int_2  (two times) | #_S3_1 to #_S3_2  (two times) |
| SNS diabtypeI 2012 and nvl/001d 2015 | - | - | #_int_1 | #_S3_1 to #_S3_4 |
| NICE diabtypeI 2015 / NICE diabtypeII 2016 and nvl/001f 2015 | #_int_1, #_S3_1 (P)  (two times) | - | #_int_2 to #_int_5  (two times) | #_S3_2 to #_S3_3  (two times) |
| ICSI diabtypeII 2014 and nvl/001f 2015 | - | - | #_int_1 | #_S3_1 to #_S3_3 |
| SNS diabtypeI 2012 and nvl/001f 2015 | - | - | #_int_1 | #_S3_1 to #_S3_3 |
| ICSI backpain 2012 and nvl/007 2011 | #_int_3, #_S3_2 (P)  #_int_6, #_S3_3 (P) | - | #_int_1, #_int_2, #_int_4, #_int_5, #_int_7 to #_int_11 | #_S3_1, _S3_4 to #_S3_7 |
| ICSI hypo 2014 and 001/018 2013 | - | - | #_int_1 | #_S3_1 to #_S3_4 |
| SIGN VTEPrev 2014 and 003/001 2015 | - | - | #_int_1, #_int_2, #_int_3a, #_int_3b, #_int_4, #_int_5 | #_S3_1, #_S3_2 |
| CCHMC VTE 2014 and 003/001 2015 | - | - | #_int_1 to #_int_4 | #_S3_1, #_S3_2 |
| NICE menstrual bleeding 2016 and 015/070 2014 | - | - | #_int_1, #_int_2 | #_S3_1 to #_S3_3 |
| NICE bipolar 2016 and 038/019 2012 | #_int_2, #_S3_18 (P)  #_int_5, #_S3_13 (P)  #_int_6, #_S3_14 (P) | - | #_int_1, #_int_3, #_int_4, #_int_7 to #_int_11 | #_S3_1 to #_S3_12, #_S3_15 to #_S3_17, #_S3_19 to #_S3_37 |
| CTFPHC obesity 2015 and 050/001 2014 | #_int_2, #_S3_9 (P) | - | #_int_1, #_int_3 | #_S3_1 to #_S3_8, #_S3_10 to #_S3_12 |
| NICE obesity 2014 and 050/001 2014 | - | - | #_int_1 to #_int_3 | #_S3_1 to #_S3_12 |
| NICE weight 2014 and 050/001 2014 | - | - | #_int_1 | #_S3_1 to #_S3_12 |
| ICSI obesity 2013 and 050/001 2014 | - | - | #_int_1 to #_int_6 | #_S3_1 to #_S3_12 |
| NICE diabpreg 2015 and 057/023 2014 | - | - | #_int_1 | #_S3_1 to #_S3_3 |
| SNS diabtypeI 2012 and 057/023 2014 | - | - | #_int_1 | #_S3_1 to #_S3_3 |
| ICSI pain 2016 and 145/003 2014 | - | - | #_int_1 to #_int_6 | #_S3_1, _S3_2 |
| SIGN pain 2013 and 145/003 2014 | - | - | #_int_1 to #_int_5 | #_S3_1, #_S3_2 |

O: outcome-indicator, P: process-indicator
