# Supplementary material for: Guideline-based quality indicators—a systematic comparison of German and international clinical practice guidelines
Source: Implement Sci. 2019 Jul 9;14:71. doi: 10.1186/s13012-019-0918-y (PMC6617919; doi:10.1186/s13012-019-0918-y)
Supplement: Supplementary file 7 — Table a. Data-Extraction of German S3-CPGs - general and methodical aspects and Table b. Data-Extraction of international CPGs - general and methodical aspects. (DOCX 60 kb) [file 13012_2019_918_MOESM7_ESM.docx]

**Additional file 7**

**Table a: Data-Extraction of German S3-CPGs - general and methodical aspects**

| **Acronym** | **Terminology QI [term]** | **QI-develop-ment group [m/e: yes (*n/NR*) / no / NR,**  **c:yes (*n/NR*) / no / NR, p:yes (*n/NR)* / no / NR],**  **other** | **Number QI [n] / QI ratio/pro-portion [n]**  *(only topic-related)* | **Structure-, process-, or outcome quality [S(*n*)/P(*n*)/O(*n*)]**  *(own assignement)* | **Explicit link to recommendation [n/N]** | **Based on strong recommendations / statements, consensus* (c)-/ evidence (e)-based**  **[c(n)/e(n)]** | **reported intended purpose of QI [yes/no]** | **reported rationale for the QI [yes/no]** | **number of reported measurement properties of QI [n/N]** | **number of reported quality objective [n/N]** | **Methods** | | | | |
| --- | --- | --- | --- | --- | --- | --- | --- | --- | --- | --- | --- | --- | --- | --- | --- |
|  |  |  |  |  |  |  |  |  |  |  | **reported external data sources for QIs [yes/no]** | **searches for existing QIs**  **[yes/no/N.R.]** | **formal consensus methods [yes/no/N.R.]** | **assessment-tools/criteria**  **[yes (tool)/ no/N.R.]** | **Evaluation**  **[yes/no/planed/N.R.]** |
| 021/007OL 2013 | QI | m/e: yes (4)  c: yes (8  p: (1) | 10/10 | 0/8/2 | 8/10 | 6/2 | yes | no | 0/10 | 0/10 | yes | no | yes | yes | N.R. |
| 021/023OL 2014 | QI | m/e: yes (4)  c: yes (10)  p: yes (1) | 12/12 | 0/7/5 | 9/12 | 6/3 | yes | no | 0/12 | 0/12 | yes | yes | yes | yes | N.R. |
| 032/009OL 2012 | QI | m/e: yes (6)  c: yes (6)  p: no | 13/13 | 0/11/2 | 11/13 | 7/4 | yes | no | 0/13 | 0/13 | no | N.R. | yes | yes | N.R. |
| 032/024OL 2016 | QI | m/e: yes (N.R.)  c: yes (N.R.)  p: yes (1)  other: yes (1) | 10/10 | 0/10/0 | 10/10 | 1/9 | yes | no | 0/10 | 2/10 | yes | yes | yes | yes | planned |
| 032/035OL 2013 | QI | m/e: yes (N.R.)  c: yes (N.R.)  p: yes (1) | 11/11 | 0/9/2 | 10/11 | 4/6 | yes | no | 0/11 | 11/11 | yes | yes | yes | yes | N.R. |
| 032/045OL 2012 | QI | m/e: yes (N.R.)  c: yes (N.R.)  p: no | 9/9 | 0/8/1 | 9/9 | 1/8 | yes | no | 0/9 | 9/9 | yes | N.R. | yes | yes | N.R. |
| 043/022OL 2014 | QI | m/e: yes (4)  c: yes (8)  p: yes (2) | 0/0 | n.a. | n.a. | n.a. | yes | no | n.a. | n.a. | N.R. | yes | yes | yes | N.R. |
| 128/001OL 2015 | QI | m/e: yes (3)  c: yes (6)  p: yes (1).  other: yes (1)  Unknown: 1 | 10/10 | 0/8/2 | 10/10 | 8/2 | yes | no | 0/10 | 3/10 | yes | yes | yes | yes | N.R. |
| arstenvl/001d 2015 | QI | m/e: yes (N.R.)  c: yes (N.R.)  p: N.R. | 4/4 | 0/4/0 | 4/4 | 0/4 | yes | no | 0/4 | 0/4 | no | yes | yes | yes | N.R. |
| nvl/001f 2012 | QI | m/e: yes (2)  c: yes (6)  p: N.R. | 3/3 | 0/3/0 | 3/3 | 0/3 | yes | no | 0/3 | 0/3 | no | yes | yes | yes | N.R. |
| nvl/007 2015 | QI | m/e: yes (1)  c: yes (25)  p: yes (1) | 7/7 | 0/7/0 | 7/7 | 0/7 | no | no | 0/7 | 7/7 | no | yes | yes | yes | planned |
| 001/018 2013 | Quality objectives and clinical measures, QI | m/e: N.R.  c: yes (6)  p: N.R. | 4/1 | 0/1/3 | 1/4 | 1/0 | yes | no | 0/4 | 0/4 | no | N.R. | N.R. | N.R. | N.R. |
| 003/001 2015 | QI | m/e: yes (3)  c: yes (N.R.)  p: N.R. | 2/2 | 0/2/0 | 2/2 | 1/1 | yes | no | 0/2 | 2/2 | yes | yes | yes | yes | N.R. |
| 015/070 2014 | QI | N.R. | 3/0 | 0/1/2 | 0/3 | n.a. | yes | yes | 0/3 | 3/3 | yes | N.R. | N.R. | N.R. | N.R. |
| 038/019 2012 | QI | N.R. | 37/37 | 0/37/0 | 37/37 | 10/0 | no | no | 0/37 | 0/37 | yes | N.R. | N.R. | N.R. | no |
| 050/001 2014 | Quality criteria | m/e: N.R.  c: yes (3).  p: N.R. | 12/0 | 7/5/0 | 12/12 | 0/0 | no | no | 0/12 | 0/12 | no | N.R. | N.R. | N.R. | N.R. |
| 057/023 2014 | recommendations for structure quality | N.R. | 3/0 | 3/0/0 | 0/3 | n.a. | no | no | 0/3 | 0/3 | no | N.R. | N.R. | N.R. | N.R. |
| 145/003 2014 | QI | N.R. | 2/0 | 0/2/0 | 2/2 | 0/0 | no | no | 0/2 | 2/2 | no | N.R. | N.R. | N.R. | N.R. |

QI: quality indicator; N.R.: not reported; n.a: not applicable; GoR: grade of recommendation; LoE: level of evidence; m: methodologists; e: experts in measuring quality; c: clinicians; p: patient, n.a.: not applicable

***** also “good clinical practice” and similiar

**Table b: Data-Extraction of international CPGs - general and methodical aspects**

| **Acronym,**  **publication date** | **Corresponding German S3-CPG [reference number]** | **Terminology QI [term]** | **QI-develop-ment group [m/e: yes (*n/NR*) / no / NR,**  **c:yes (*n/NR*) / no / NR, p:yes (*n/NR)* / no / NR],**  **other** | **Number QI [n] / QI ratio/pro-portion [n]**  *(only topic-related)* | **Structure-, process-, or outcome quality [S(*n*)/P(*n*)/O(*n*)]**  *(own assignement)* | | **Explicit link to recommendation [n/N]** | **Based on strong recommendations / statements, consensus* (c)-/ evidence (e)-based**  **[c(n)/e(n)]** | **reported intended purpose of QI [yes/no]** | **reported rationale for the QI [yes/no]** | **number of reported measurement properties of QI [n/N]** | **number of reported quality objective [n/N]** | **Methods** | | | | |
| --- | --- | --- | --- | --- | --- | --- | --- | --- | --- | --- | --- | --- | --- | --- | --- | --- | --- |
|  |  |  |  |  |  |  |  |  |  |  |  |  | **reported external data sources for QIs [yes/no]** | **searches for existing QIs**  **[yes/no/N.R.]** | **formal consensus methods [yes / no / N.R.]** | **assessment-tools/criteria**  **[yes (tool) / no / N.R.]** | **Evaluation**  **[yes/no/planed/N.R.]** |
| CTFPHC colorectal 2016 | 021/007OL 2013 | performance measure | N.R. | 6/2 | | 0/3/3 | 0/6 | n.a. | no | no | 0/6 | 0/6 | no | N.R. | N.R. | N.R. | N.R. |
| SIGN colorectal 2016 |  | key points to audit | m/e: yes (3  c: yes (14)  p: yes (1) | 6/0 | | 0/5/1 | 0/6 | n.a. | yes | no | 0/6 | 0/6 | no | N.R. | N.R. | N.R. | N.R. |
| KCE gastrointest 2012 | 021/023OL 2014, | QI | m/e: yes (N.R.)  c: yes (16)  p: N.R. | 15/14 | | 0/9/6 | 8/14 | 0/8 | yes | yes | 0/15 | 0/15 | yes | yes | yes | yes | yes |
|  | 032/009OL 2012 |  |  | 14/13 | | 0/7/7 | 7/13 | 0/7 |  |  | 0/14 | 0/14 |  |  |  |  |  |
| SIGN melanoma 2017 | 032/024OL 2016 | quality performance indicator | N.R. | 13/13 | | 0/13/0 | 0/13 | n.a. | yes | no | 0/13 | 0/13 | yes | N.R. | N.R. | N.R. | N.R. |
| SIGN ovar 2013 | 032/035OL 2013 | quality performance indicator | N.R. | 13/13 | | 0/11/2 | 0/13 | n.a. | yes | no | 0/13 | 0/13 | yes | N.R. | N.R. | N.R. | N.R. |
| SIGN breast 2013 | 032/045OL 2012 | key points to audit | m/e: yes (2)  c: yes (12)  p: yes (1) | 4/3 | | 0/2/2 | 0/4 | n.a. | yes | no | 0/4 | 0/4 | no | N.R. | N.R. | N.R. | N.R. |
| CTFPHC prostate 2014 | 043/022OL 2014 | performance indicator | N.R. | 3/3 | | 0/3/0 | 0/3 | n.a. | no | no | 0/3 | 0/3 | no | N.R. | N.R. | N.R. | N.R. |
| ICSI palliative 2013 | 128/001OL 2015 | measure | N.R. | 10/10 | | 2/8/0 | 10/10 | 0/10 | yes | no | 0/10 | 10/10 | no | N.R. | N.R. | N.R. | N.R. |
| NICE diabtypeI 2015 | nvl/001d 2015 | (NICE) indicator | m/e: yes (N.R.)  c: yes (N.R.)  p: yes (N.R.). | 4/4 | | 0/4/0 | 4/4 | 0/4 | yes | no | 0/4 | 0/4 | no | N.R. | N.R. | yes | yes |
| NICE diabtypeII 2016 |  |  |  |  |  |  |  |  |  |  |  |  |  |  |  |  |  |
| SNS diabtypeI 2012 |  | indicator | N.R. | 1/1 | | 0/0/1 | 0/1 | n.a. | yes | no | 0/1 | 0/1 | no | N.R. | N.R. | N.R. | N.R. |
| NICE diabtypeI 2015 | nvl001f 2012 | (NICE) indicator | m/e: yes (N.R.)  c: yes (N.R.)  p: yes (N.R.) | 5/5 | | 0/5/0 | 5/5 | 0/5 | yes | no | 0/5 | 0/5 | no | N.R. | N.R. | yes | yes |
| NICE diabtypeII 2016 |  |  |  |  |  |  |  |  |  |  |  |  |  |  |  |  |  |
| ICSI diabtypeII 2014 |  | measure | N. R. | 1/1 | | 0/1/0 | 0/1 | n.a. | yes | no | 0/1 | 1/1 | no | N.R. | N.R. | N.R. | N.R. |
| SNS diabtypeI 2012 |  | indicator | N.R. | 1/0 | | 0/1/0 | 0/1 | n.a | yes | no | 0/1 | 0/1 | no | N.R. | N.R. | N.R. | N.R. |
| ICSI backpain 2012 | nvl/007 2011 | measure | N.R. | 11/11 | | 0/11/0 | 11/11 | 0/11 | no | no | 0/11 | 11/11 | no | N.R. | N.R. | N.R. | N.R. |
| ICSI hypo 2014 | 001/018 2013 | measure | N.R. | 1/1 | | 0/1/0 | 0/1 | n.a. | yes | no | 0/1 | 1/1 | yes | N.R. | N.R. | N.R. | N.R. |
| SIGN VTEPrev 2014 | 003/001 2015 | key points to audit | m/e: yes (3)  c: yes (12)  p: no  other: yes (2) | 6/3 | | 0/6/0 | 0/6 | n.a | yes | no | 0/6 | 0/6 | no | N.R. | N.R. | N.R. | N.R. |
| CCHMC VTE 2014 |  | outcome measure, process measure | N.R. | 4/0 | | 0/3/1 | 0/4 | n.a. | yes | no | no | 4/4 | no | N.R. | N.R. | N.R. | N.R. |
| NICE mens 2016 | 015/070 2014 | (NICE) indicator | m/e: yes (N.R.)  c: yes (N.R.)  p: yes (N.R.) | 2/2 | | 0/0/2 | 2/2 | 0/2 | yes | no | 0/2 | 0/2 | no | N.R. | N.R. | yes | yes |
| NICE bipolar 2016 | 038/019 2012 | (NICE) indicator | m/e: yes (N.R.)  c: yes (N.R.)  p: yes (N.R.) | 11/11 | | 0/11/0 | 9/11 | 0/9 | yes | no | 0/11 | 0/11 | no | N.R. | N.R. | yes | yes |
| CTFPHC obesity 2015 | 050/001 2014 | performance indicator | N.R. | 3/3 | | 0/2/1 | 0/3 | n.a. | no | no | 0/3 | 0/3 | no | N.R. | N.R. | N.R. | N.R. |
| NICE obesity 2014 |  | (NICE) indicator | m/e: yes (N.R.)  c: yes (N.R.)  p: yes (N.R.) | 3/2 | | 1/2/0 | 3/3 | 0/3 | yes | no | 0/3 | 0/3 | no | N.R. | N.R. | yes | yes |
| NICE weight 2014 |  | (NICE) indicator | m/e: yes (N.R.)  c: yes (N.R.)  p: yes (N.R.) | 1/1 | | 0/1/0 | 1/1 | 0/1 | yes | no | 0/1 | 0/1 | no | N.R. | N.R. | yes | yes |
| ICSI obesity 2013 |  | measure | N. R. | 6/6 | | 0/4/2 | 6/6 | 0/6 | yes | no | 0/6 | 6/6 | no | N.R. | N.R. | N.R. | N.R. |
| NICE diabpreg 2015 | 057/023 2014 | (NICE) indicator | m/e: yes (N.R.)  c: yes (N.R.)  p: yes (N.R.) | 1/1 | | 0/1/0 | 1/1 | 0/1 | yes | no | 0/1 | 0/1 | no | N.R. | N.R. | yes | yes |
| SNS diabtypeI 2012 |  | indicator | N.R. | 1/1 | | 0/0/1 | 0/1 | n.a. | yes | no | 0/1 | 0/1 | no | N.R. | N.R. | N.R. | N.R. |
| ICSI pain 2016 | 145/003 2014 | measure | N.R. | 6/6 | | 0/6/0 | 6/6 | 0/0** | yes | no | 0/6 | 6/6 | no | N.R. | N.R. | N.R. | N.R. |
| SIGN pain 2013 |  | key points to audit | m/e: yes (2)  c: yes (19)  p: yes (2) | 5/0 | | 0/4/1 | 0/5 | n.a. | yes | no | 0/5 | 0/5 | no | N.R. | N.R. | N.R. | N.R. |

QI: quality indicator; N.R.: not reported; n.a: not applicable; GoR: grade of recommendation; LoE: level of evidence; m: methodologists; e: experts in measuring quality; c: clinicians; p: patient, n.a.: not applicable

***** also “good clinical practice” or “expert opinion”

** GoR/ LoE N.R.
